# Accessible Chemical Benchmarking of Nanoparticle Growth Using Oxidation–Reduction Potential Sensors

**DOI:** 10.1021/acs.chemmater.6c01559

**Published:** 2026-08-27

**Authors:** Gabriel C. Halford, Sebastian Hertle, Katrina L. Obedin, Abigail M. Sublett, Kaelyn M. Harmon, Lauren E. VanGelder, Michelle L. Personick

**Affiliations:** † Department of Chemistry, 2358 University of Virginia, Charlottesville, Virginia 22904, United States; ‡ Department of Materials Science and Engineering, University of Virginia, Charlottesville, Virginia 22904, United States; § Department of Chemistry, 6041 Norfolk State University, Norfolk, Virginia 23504, United States

## Abstract

Open-circuit potential (OCP) measurements are an emerging technique for capturing the real time, in situ chemistry of metal nanoparticle syntheses to both (1) understand detailed chemical mechanisms of nanoparticle growth and (2) benchmark the chemistry of nanoparticle formation to improve reproducibility. In this work, we use oxidation–reduction potential (ORP) sensors as an experimentally facile alternative to potentiostat-based methods for acquiring benchmark OCP measurements, which increases the accessibility of this approach to the broader materials chemistry community. We also expand implementation of the OCP method to include non-noble metal materials and more complex reaction conditions by benchmarking copper (Cu) nanoparticle growth at elevated temperatures with both ORP sensors and potentiostat OCP measurements. Additionally, we demonstrate the application of ORP sensors in troubleshooting the detrimental or beneficial influence of impurities in the widely used surfactant cetyltrimethylammonium chloride (CTAC) to ensure reproducible, high quality nanoparticle synthesis. Overall, we establish that ORP sensors are a viable alternative to potentiostat-based OCP measurements for benchmarking and troubleshooting, while also providing important considerations their use in certain mechanistic studies and growth solution chemistries.

## Graphical abstract

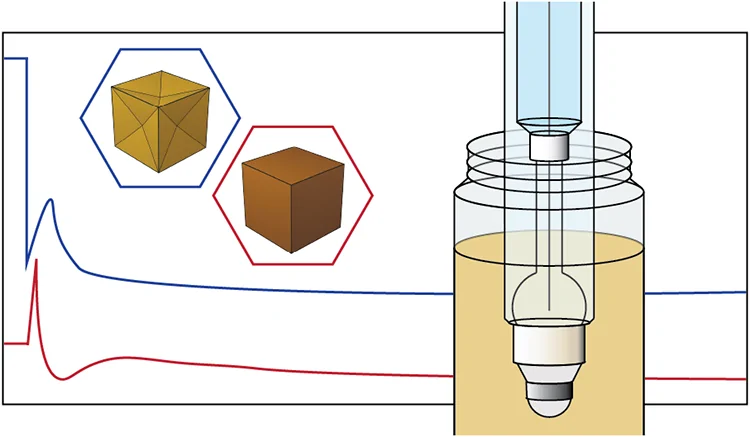

## 1 Introduction

Meeting growing demands for highly tailored nanomaterials for specialized applications, such as thermal heterogeneous catalysis and electrocatalysis,^1–5^ sensing,^6,7^ and nanomedicine,^8,9^ requires fine-tuning of morphological properties such as faceted structure, degree of anisotropy, and size. Development of reproducible protocols for the synthetic control of these properties is therefore paramount. Despite significant expansion in the variety of architectures and compositions of synthetically accessible nanomaterials, as well as advancements in the understanding of chemical principles underlying nanoparticle growth,^10–20^ synthetic reproducibility remains a particular challenge.^21–23^ Factors contributing to irreproducibility include the sensitivity of kinetically controlled syntheses to imprecisely defined procedural variables, such as swirling or ambient temperature, as well as trace chemical impurities in commercial reagents and deionized water that have been extensively described in the literature.^21–41^

Anticipating the presence and type of synthetically relevant impurities in reagents is challenging, as is the identification of specific impurities once a synthetic reproducibility problem occurs. Since morphology-directing impurities in common reagents for nanomaterials synthesis are known to range from halides^27,29,42^ to metal ions^31^ to small organic solvent molecules^24^ to organic isomeric impurities^34^ to differing polymer end groups or chain lengths,^36,38,39^ a variety of different techniques are required to successfully identify them. Given this challenge, an alternative approach for troubleshooting reproducibility issues during nanoparticle synthesis is to compare the chemical conditions during growth of an unsuccessful synthesis attempt with a corresponding “benchmark” of a successful synthesis. The difference in chemical environments between the unsuccessful synthesis attempt and the benchmark can then inform troubleshooting, via modification of synthetic parameters such as pH, temperature, or shape-directing additives.

Recently, our group has introduced open-circuit potential (OCP) measurements of metal nanoparticle growth solutions as a real time, in situ tool for measurement of the solution chemistry of colloidal noble metal nanoparticle growth.^21,24,43,44^ In an OCP measurement, the potential in an electrochemical cell is measured between a working and reference electrode in the absence of any netcurrent flow (and in the absence of applied potential or current).^45^ This potential reflects the sum of the “mixture” of cathodic and anodic partial currents at the working electrode (WE), arising from all chemical reactions (and physical processes affecting chemical reactions) at or near the WE. In metal nanoparticle growth solutions, which are not solely dominated by a single redox couple at equilibrium, the OCP does not follow the Nernstian equilibrium potential, and is referred to as a mixed solution potential.^45–47^ This mixed potential may include contributions from metal ion reduction, reducing agent oxidation, any changes in reagent speciation, changes in pH, the dissolved oxygen concentration, oxygen reduction, and other ionic species in solution (halides, ionic surfactants, acid/base additives, etc.).^45,47,48^ As the balance of these different contributions changes over the course of a nanoparticle growth reaction, the mixed solution potential (measured OCP) will change. Of note, the presence of any chemical impurities that significantly affect the kinetics of metal ion reduction/reducing agent oxidation during metal nanoparticle growth will cause a change in the measured OCP.

The responsive nature of OCP measurements to changes in growth solution chemistry and nanoparticle growth kinetics means that they can be used both to better understand the chemical environment in which nanoparticle growth occurs^43,44^ and to troubleshoot synthesis-to-synthesis variability.^24^ The OCP technique introduces advantages compared to other methods for time-resolved monitoring of nanoparticle growth chemistry, as other established methods are frequently some combination of costly, time-consuming, or limited to a specific subset of metal chemistries.^21^ OCP measurements combine a simple and comparatively inexpensive^21^ experimental setup with real-time, continuous data acquisition, enabling the generation of benchmark measurements of successful syntheses and streamlining synthetic troubleshooting.

In our previous work, we used OCP measurements to troubleshoot an irreproducibility issue in the synthesis of palladium (Pd) tetrahexahedra (THH)—arising from line-to-line and lot-to-lot differences in both small organic molecule and iodide contaminant concentrations in the cetyltrimethyl-ammonium bromide (CTAB) surfactant used in the synthesis—through comparison of OCP measurements of unsuccessful synthesis attempts with a benchmark measurement of a successful THH synthesis.^24^ The differences between these measurements and the benchmark were used to directly select optimal concentrations of kinetics-directing additives to achieve OCP traces in agreement with the THH benchmark and corresponding successful formation of THH. This process ultimately shortened the time scale of troubleshooting this synthesis with new CTAB lines from years to days.

While OCP measurements are commonly acquired using a potentiostat, a potentiostat is not strictly required because OCP is a passive measurement and does not involve the passage of a netcurrent. Commercially available oxidation–reduction potential (ORP) sensors can be used without a potentiostat to take measurements of the redox activity of solutions (Figure 1). This measurement is typically referred to as the “ORP” of the analyte solution but is conceptually identical to the OCP measured by a potentiostat: it is the mixed potential of all electroactive species in solution. ORP sensors are constructed with a reference electrode (RE, typically a silver/silver chloride (Ag/AgCl) RE in potassium chloride (KCl) electrolyte) and a WE, connected with high-impedance internal electronics (Figure 1). The WE is typically either a noble metal wire or band. There is no counter electrode (CE). When the ORP sensor is immersed into a solution of interest, the WE is in direct contact with the solution, while the RE is separated by a porous liquid junction from the solution, which prevents contamination but allows for ion exchange with the KCl electrolyte. Basic commercially available ORP sensors have a single junction between the RE and the analyte, but ORP sensors with multiple junctions (with the “intermediate” compartments also filled with KCl electrolyte) can also be purchased—similar to the use of bridge tubes in traditional electrochemistry experiments. Immersion of the ORP sensors in a solution creates a two-electrode electrochemical cell which can be used for open-circuit measurements, but not for the application of potential or current. ORP sensors are widely used in quality assurance, personal consumer, and academic contexts for study of water contaminants and water quality treatments.^49–53^ However, ORP sensors have not otherwise been utilized to study materials growth, with the exception of their use for comparison with a Pourbaix diagram to determine Cu ion speciation under various basic pH conditions in a synthesis of copper (Cu) nanoparticles.^54^

**Figure 1:**
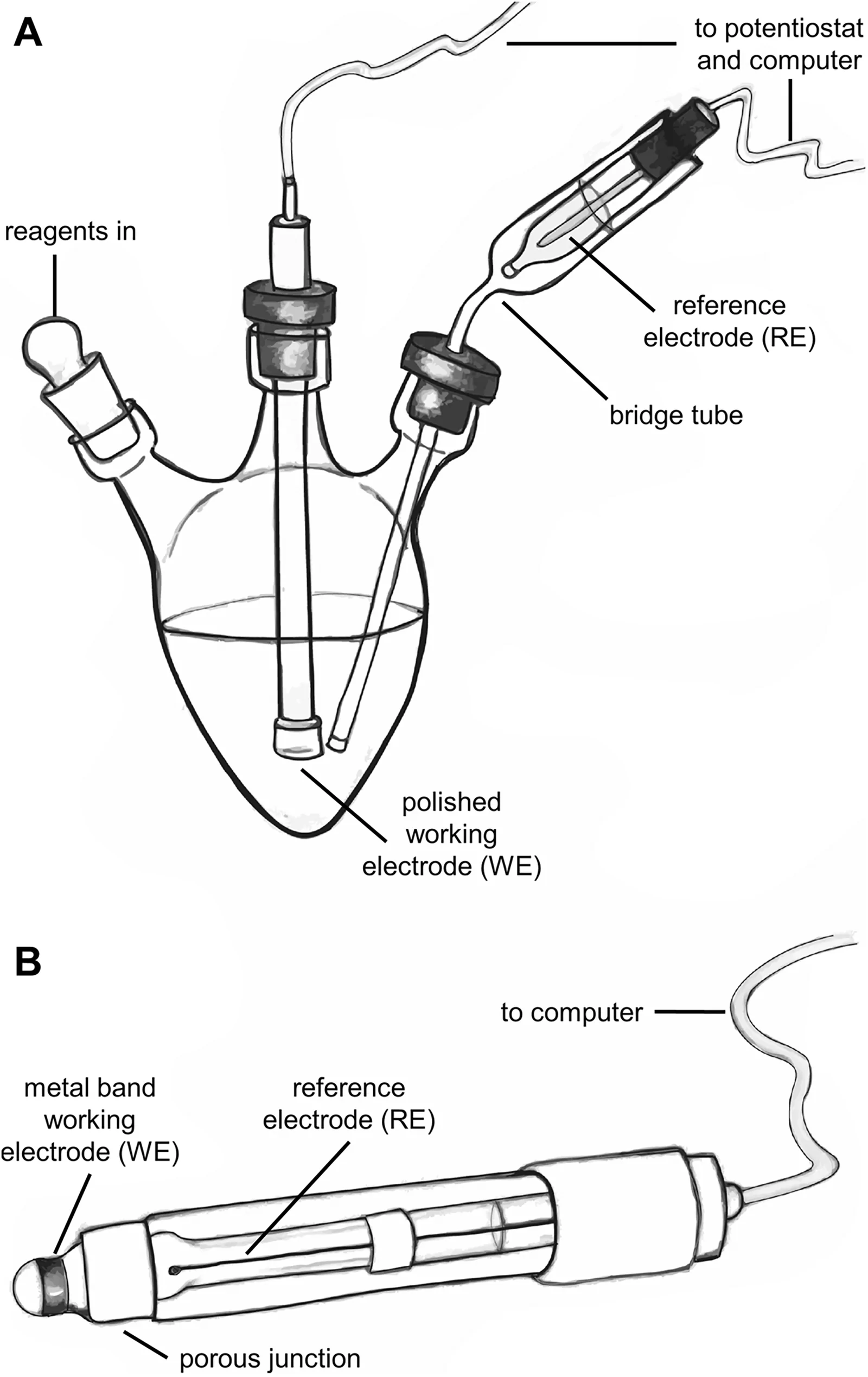
Scheme comparing (A) potentiostat OCP and (B) ORP sensor electrodes and glassware (double-junction ORP sensor shown).

ORP sensors present a variety of possible advantages as a tool for taking OCP measurements. While the potentiostat-based OCP technique is already facile and inexpensive compared to other available time-resolved techniques for characterizing metal nanoparticle growth,^21^ the very low cost and ease of use of ORP sensors could increase the accessibility of the technique across research groups. For OCP measurements to be useful in benchmarking, troubleshooting, and standardization of synthesis, access to a large data set comprising OCP measurements of “successful” and relevant “failed” synthesis attempts is needed. A decrease in the cost and technical barrier to entry would allow for a greater number of research groups to routinely collect benchmark measurements of their syntheses, accelerating the synthetic optimization process when troubleshooting is required. In this work, we present proof-of-concept for the use of ORP sensors to make chemically meaningful, time-resolved measurements of Pd, gold (Au), (Ag)Au (dilute surface coverage of Ag), and Cu nanoparticle syntheses. Additionally, we use ORP sensors to successfully troubleshoot a synthetic reproducibility issue involving impurity differences in cetyltrimethylammonium chloride (CTAC) between manufacturers. Overall, ORP sensors present a viable lower-cost alternative to potentiostat OCP for real-time monitoring of metal nanoparticle growth chemistry if electrode composition is appropriately considered.

## 2 Results and Discussion

### 2.1 Validation of ORP Sensor-Based Measurements in Palladium Nanoparticle Growth

To evaluate the feasibility of using an ORP sensor in place of a potentiostat to measure the real-time mixed potential in nanoparticle synthesis, we assessed the accuracy and reproducibility of ORP sensor measurements in comparison to our group’s previously reported OCP measurements of Pd nanoparticle syntheses.^24,43,44^ Measurements obtained using a two-electrode cell and a potentiostat (Gamry Instruments, Interface 1010E) will be referred to as “potentiostat OCP.” The standard potentiostat OCP setup used a glassy carbon electrode (GCE) as the WE and a Ag/AgCl RE (saturated KCl) or a Hg/Hg_2_SO_4_ RE (saturated K_2_SO_4_), separated from the reaction mixture by a bridge tube containing 0.1 M H_2_SO_4_ electrolyte. Measurements made using ORP sensors, obtained from multiple vendors, will be named according to sensor type. For example, the single-junction (SJ) epoxy-body ORP sensor from Vernier used for this set of experiments will be referred to as “SJ epoxy ORP.” The SJ epoxy ORP sensor is composed of a Pt band WE and an Ag/AgCl RE, with one porous junction separating the reference electrode from the analyte solution. An overview of the ORP sensors used in this work and their specifications can be found in Table S1. Detailed information on handling and care of the ORP sensors can be found in Section 3.4 of the SI. In some cases, appropriate selection of ORP sensor type is important for ensuring accurate and reproducible measurements (vide infra).

As an initial validation experiment, we measured a synthesis for twinned, corrugated Pd nanoparticles, as this synthesis is highly reproducible and mechanistically well-characterized.^43,55^ The synthesis is conducted in a mixture of CTAC and dilute CTAB surfactants. The CTAC was purchased as an aqueous 25 wt % solution from MilliporeSigma—referred to as “Sigma CTAC” throughout this work. Along with the surfactant mixture, the growth solution contains sodium tetrachloropalladate (Na_2_PdCl_4_), nitric acid (HNO_3_), ascorbic acid (AA), and pseudospherical Pd seed particles (<10 nm) diluted in a CTAB solution.^55^ The products of this synthesis are a mixed population of singly twinned right bipyramids, pentatwinned rods, and 20-fold twinned concave icosahedra, all with finned structures and surface corrugation. SJ epoxy ORP measurements of the OCP of the synthesis, measured in a scintillation vial, correspond well with the potentiostat OCP measurements (GC WE vs Ag/AgCl RE),^43^ with a solution potential of +355 ± 10 mV vs SHE (Figure 2A). SEM images confirmed that the ideal corrugated particle morphology was still produced in each of the measured reactions (Figures 2C–D and S1). These data show that there is minimal influence of the ORP sensor body and electrode materials, the different electrode geometries, or the different sizes and glass types of the two reaction vessels on either the product morphologies or the ORP/OCP measurements of this synthesis.

**Figure 2:**
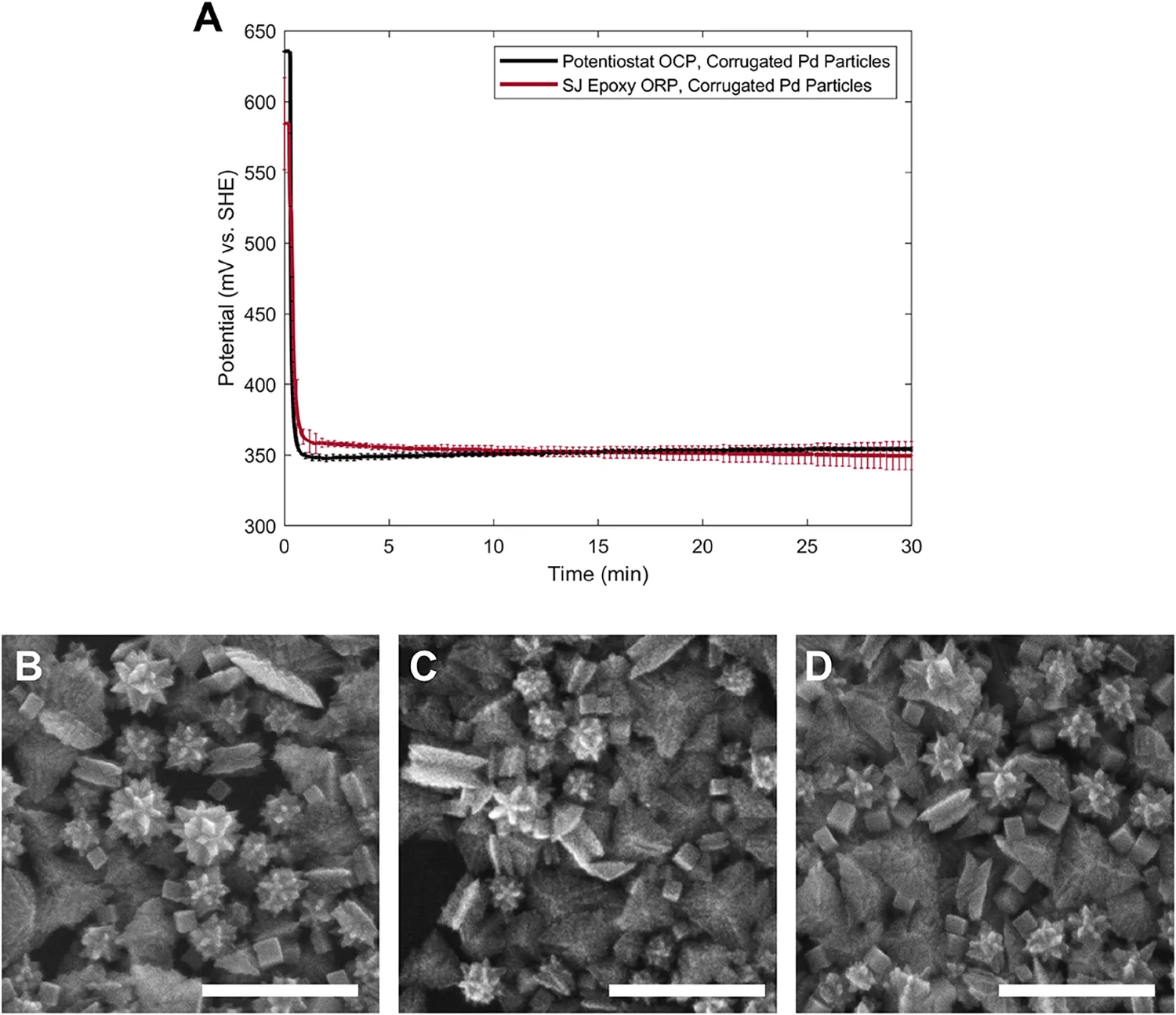
(A) Comparison between potentiostat OCP measurements using a GC WE vs a Ag/AgCl RE (black) and SJ epoxy ORP measurements (Pt band WE vs Ag/AgCl RE; red) of corrugated Pd nanoparticle growth. Error bars represent the standard deviation of three measurements. (B-D) SEM images of corrugated Pd nanoparticles, (B) unmeasured during synthesis, or measured during synthesis with (C) potentiostat OCP using a GC WE vs a Ag/AgCl RE, and (D) a SJ epoxy ORP sensor. Scale bars: 500 nm.

To investigate the sensitivity of ORP sensor measurements to changes in reaction chemistry during nanoparticle growth, measurements of the Pd THH synthesis described in the Introduction were also taken with a SJ epoxy ORP sensor and compared with our previously reported potentiostat OCP data, which used a GC WE vs a Hg/Hg_2_SO_4_ RE.^24,44^ Shape development in this synthesis is highly kinetically controlled,^24^ and early changes in the metal ion reduction rate are critical for directing the emergence of the desired THH shape hours later.^44^ This is observed in the potentiostat OCP data as a dip in potential during the first ∼10 min of reaction, which is associated with an initial fast rate of metal ion reduction and rapid depletion of reagents around seed particles, a second period of slow or stagnant growth while reagents diffuse to seed particles, and finally the development of a steady-state metal ion reduction rate for the remainder of the reaction.^44^

The SJ epoxy ORP measurements of this reaction do not show this initial dip in potential, but do match the potentiostat OCP measurements (±10 mV) after the first ∼12 min of measurement, during the time period of steady-state metal ion reduction rate (Figure 3).^44^ The THH shape morphology developed as expected during the ORP measurements, implying that the required change in solution potential associated with local reagent depletion does happen, but is not captured by the SJ epoxy ORP sensor (Figure 3 and Figure S2 and Table S2). To identify the cause of the loss of this dip feature, potentiostat OCP measurements of the Pd THH synthesis were performed with several Pt WEs for comparison to the Pt band WE of the SJ epoxy ORP sensor. When a planar, polished Pt disk WE was used for potentiostat OCP, this dip was appropriately captured. The initial drop in potential was more rapid than with the typical GC WE, which we attribute to the faster electron transfer (ET) between the WE and analytes when a pristine noble metal WE is used, as compared to GC or other electrode materials (Figure 3A–B).^56–59^ The faster ET in this case causes more rapid responsiveness to the initiation of particle growth with seeds and AA; this may be additionally enhanced by the particular affinity of Pt for AA.^60^ Meanwhile, when the Pt disk WE was allowed to oxidize in air prior to measurement or when an unpolished Pt wire was used as the WE—similar to the unpolished and likely oxidized Pt band WE of the SJ epoxy ORP sensor—the dip feature was not seen at all (Figure 3A–B). We hypothesize that slower ET and lower responsiveness of oxidized metal WE materials prevents the sensing of the initial dip in the Pd THH OCP, because this is related to rapid, subtle local changes in the depletion and re-establishment of reagent concentration gradients around seed particles. As the steady-state rate of metal ion reduction develops, in the period following this dip, OCP with a less-responsive, oxidized RE becomes sufficient to capture the solution chemistry, and the inherent ET rate difference between GC and Pt electrodes also ceases to affect the measured OCP.

**Figure 3:**
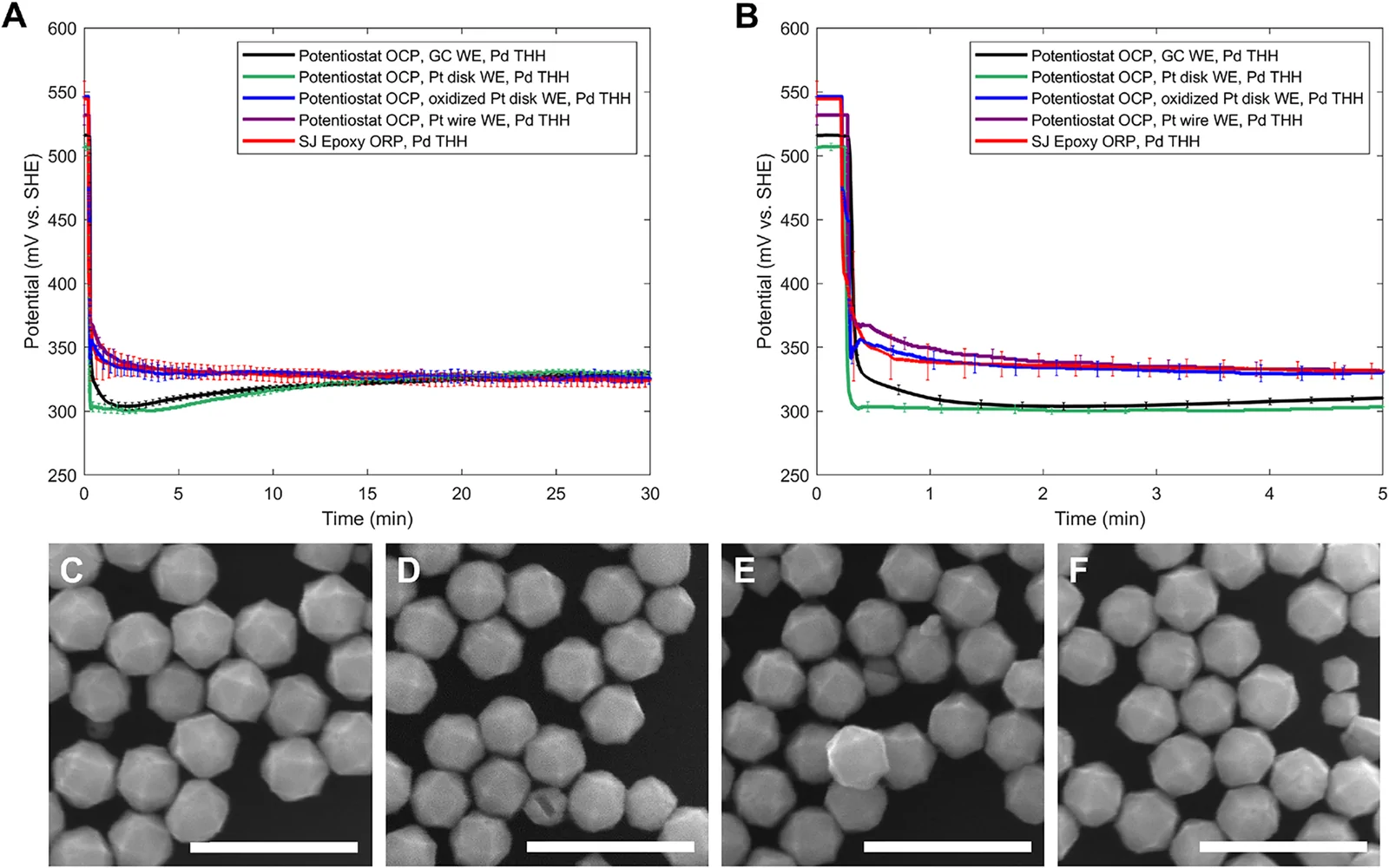
(A) Comparison between potentiostat OCP measurements using a GC WE vs a Hg/Hg_2_SO_4_ RE (black), a Pt disk WE vs a Hg/Hg_2_SO_4_ RE (green), an oxidized Pt disk WE vs a Hg/Hg_2_SO_4_ RE (blue), a Pt wire WE vs a Hg/Hg_2_SO_4_ RE (purple), and SJ epoxy ORP measurements (Pt band WE vs Ag/AgCl RE; red) of the first 30 min of Pd THH growth. Error bars represent the standard deviation of three measurements. (B) Zoomed-in view of the first 5 min of the OCP traces shown in panel (A). (C–F) SEM images of Pd THH, measured during synthesis with (C) potentiostat OCP using a Pt disk WE vs a Hg/Hg_2_SO_4_ RE (green trace), (D) potentiostat OCP using an oxidized Pt disk WE vs a Hg/Hg_2_SO_4_ RE (blue trace), (E) potentiostat OCP using a Pt wire WE vs a Hg/Hg_2_SO_4_ RE (purple trace), and (F) a SJ epoxy ORP sensor (Pt band WE vs Ag/AgCl RE; red trace). Scale bars: 500 nm. Black trace in (A) adapted with permission from ref 44. Available under a Creative Commons CC-BY 4.0 license. Copyright 2026 Halford, Sublett, and Personick.

This experiment identifies some limitations in the response rate of ORP sensors to dynamic changes in solution potential compared to OCP measurements, likely due to differences in electrode materials and treatment. Nevertheless, based on the good agreement between the steady-state solution potentials of the Pd corrugated particles and Pd THH with both measurement approaches, the SJ epoxy ORP sensor appears to be a viable option for accurate and reproducible OCP measurements of the steady-state growth of Pd nanoparticles, provided that resolution of subtle dynamics early in the reaction is not necessary.

### 2.2 Development of Protocols for ORP Sensor Measurements of Gold Nanoparticle Growth

Au nanoparticle syntheses are widely studied and of broad interest for a range of applications, therefore we also investigated Au nanoparticle growth using ORP sensor measurements. For simplicity in our initial experimental design, we considered a model Au nanoparticle synthesis, for which we recently reported potentiostat OCP measurements, with a growth solution containing only Sigma CTAC surfactant; chloroauric acid (HAuCl_4_) as a Au^3+^ ion source; hydrochloric acid (HCl) to tune pH (pH = ∼1.8); AA, in a 2-fold stoichiometric excess to HAuCl_4_, as the reducing agent; and dilute Au seeds (∼7 nm).^39^ In this synthesis, Au^3+^ is rapidly reduced to Au^+^ with the addition of AA, but the comparatively weak reducing strength of AA at the low pH of the growth solution and the stabilization of Au^+^ species by complexation with Cl^–^ ions prevent the further reduction of Au^+^ to Au^0^ until Au seeds are added to the growth solution.^11,61–65^ SEM images of the products of this simplified Au growth solution show a mixture of {111}-faceted octahedra, plates, and bitetrahedra, as well as a smaller population of nanorods (Figures S3 and S4). The mixed potential for this synthesis stabilized at around +350 ± 20 mV vs SHE when measured via potentiostat OCP using a Hg/Hg_2_SO_4_ RE (Figure 4).^44^

**Figure 4:**
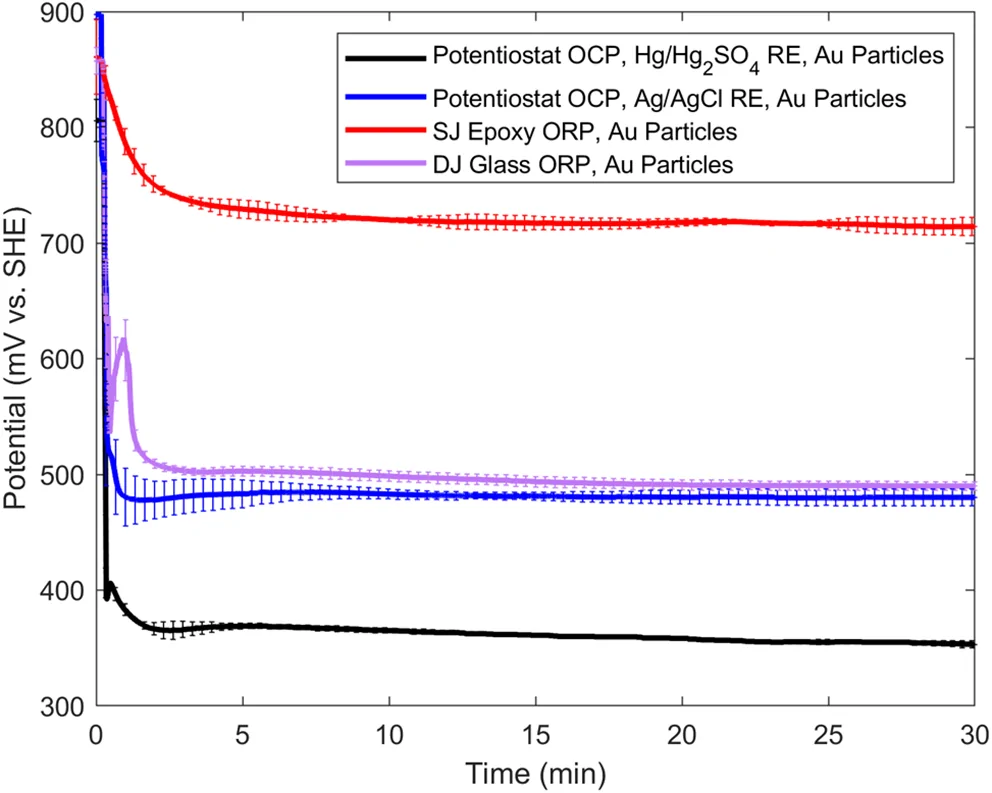
Comparison between measurements of monometallic Au nanoparticle synthesis in Sigma CTAC taken with potentiostat OCP using a GC WE vs a Hg/Hg_2_SO_4_ RE (black); potentiostat OCP using a GC WE vs a Ag/AgCl RE (blue); a SJ epoxy ORP sensor (Pt band WE vs Ag/AgCl RE; red); and a DJ glass ORP sensor (Pt band WE vs Ag/AgCl RE; purple). Error bars represent the standard deviation of three measurements. Black trace adapted with permission from ref 44. Available under a Creative Commons CC-BY 4.0 license. Copyright 2026 Halford, Sublett, and Personick.

Unlike for Pd syntheses, the potentials measured using a SJ epoxy ORP sensor for these Au nanoparticle syntheses did not align with the OCP measurements, despite the reactions producing approximately the same size and dispersity of particles (Figures 4, S3–S4, and Table S3). These results imply that the ORP sensor, its electrode materials, or the change in glassware and electrode geometry influence the electroanalytical measurement in a way that is different from the potentiostat OCP materials and setup, although the sensor does not significantly influence the development of the products themselves. This was expected, and in previous work we had intentionally used a Hg/Hg_2_SO_4_ RE for OCP measurements of Au nanoparticles instead of Ag/AgCl to avoid leaching of Ag^+^ ions.^44,66,67^ Surface passivation by Ag is known to significantly slow the Au ion reduction kinetics for this synthetic system with the addition of as little as ∼0.92 μM Ag^+^ (1 μL of 10 mM AgNO_3_) to the growth solution.^11,44^ Indeed, when the potentiostat OCP of the Au model synthesis was measured with a Ag/AgCl RE, the measured OCP was shifted ∼125 mV more positive (less strongly reducing) in potential than when it was measured vs a Hg/Hg_2_SO_4_ RE (all potentials converted to standard hydrogen electrode (SHE) scale)—closer to but still ∼225 mV less positive than measurements taken with a SJ epoxy ORP sensor (Figures 4 and S3D). When the Au nanoparticle solution was analyzed using ICP-OES following measurement with an Ag/AgCl reference electrode, [Ag] was below the ∼1 ppm limit of detection (equal to approximately 9 μM Ag^+^ in the growth solution). The concentration of the leached Ag^+^ contaminant was posited to be <0.9 μM (900 nM) because 0.9 μM Ag^+^ is known to yield octahedra (sometimes with a small subpopulation of single-twinned {111} bitetrahedra), rather than the polydisperse mixture of octahedra, bitetrahedra, plates and multiply twinned products observed from the Au-only growth solution.^11,62^ As predicted, the addition of ≤230 nM Ag^+^ to Au growth solutions led to a mixture of twinned {111}-faceted products and octahedra (Figure S5B–C), while addition of 230 nM < *x* ≤ 900 nM Ag^+^ produced primarily single-crystalline octahedra (Figure S5D). The potentiostat OCP of a Au growth solution with 230 nM added Ag^+^ measured vs a Hg/Hg_2_SO_4_ RE was within 10 mV of measurements of the growth reaction with no added Ag^+^ vs a Ag/AgCl RE, indicating similar reaction kinetics and confirming this level of Ag^+^ leaching from the RE (Figure S5A).

Since Ag^+^ leaching from the RE only explains around 120–125 mV of the ≥350 mV difference in potential between potentiostat OCP measurements with a Hg/Hg_2_SO_4_ RE and SJ epoxy ORP measurements of Au growth solutions, consideration of the different WE materials in the potentiostat OCP vs SJ epoxy ORP sensor measurements was also necessary. For potentiostat OCP measurements, we used a polished GCE (19.64 mm^2^ surface area; SA) as the WE, while the SJ epoxy ORP sensor has a small (∼6 mm^2^ SA) Pt band WE. Potentiostat OCP measurements of Au growth solutions with a Pt wire WE (∼24 mm^2^ SA in contact with the growth solution) and Ag/AgCl RE were therefore carried out to better interpret the effects of the WE size and material of the SJ epoxy ORP sensor. The potentiostat OCP with the Pt wire WE was 180 ± 25 mV more positive than the same measurement with a GCE as the WE (both vs Ag/AgCl) and significantly closer to the value measured by SJ epoxy ORP (Figure S6). Of note, the sharp increase in potential in the first minute of the potentiostat OCP measurements with Pt wire working electrodes is attributed to the presence of a Pt oxide species on the unpolished wire surface and the wire’s nonplanar geometry, which cause a nonideal electrode response to the significant [AA] change due to rapid Au^3+^ reduction to Au^+^ by AA. This feature does not appear as prominently in potentiostat OCP measurements taken with a GC WE or with a polished, planar Pt disk WE, but it is observed when the planar disk WE is allowed to oxidize in air for ≥2 h prior to use in potentiostat OCP measurements (Figure S7). More importantly for understanding the overall increase in measured potential, Au deposition on the Pt wire WE was visually apparent after approximately 90 s of measurement (Figure S6B–C). While some Au deposition on the SJ epoxy ORP sensor body following use was observed in ORP experiments, the geometry of the sensor and small size of the WE and the RE junction led to challenges in visually observing Au deposition on either component. A positive shift in the measured OCP due to Au deposition on the WE is supported by literature reports of similar shifts in the measured OCP of electroactive solutions when the WE material is modified.^56–59^ In addition, other literature reports mention electroless deposition of an Au adlayer onto Pt electrode substrates immersed in Au salts at open-circuit potential, either when Pt surface atoms are oxidized or when a chemical reductant is present.^68,69^

To directly investigate whether Au deposition on the Pt WE is a significant contributor to the mixed potential measurements, a potentiostat OCP measurement was taken using Pt mesh WE with a large (∼1000 mm^2^) surface area vs a Hg/Hg_2_SO_4_ RE. Following slightly different behavior in the first ∼5 min of measurement, where the OCP is dominated by reagent transport/diffusion^44^ and therefore is likely particularly sensitive to WE geometry, the measured potentials with a Pt mesh WE were within 10 mV of the standard potentiostat OCP measurement with a GC WE for the remainder of the standard 30 min measurement period (Figure S8A). No Au deposition was visually noted on the Pt mesh after 30 min (Figure S8C). When the measurement was continued for a longer time period, a nearly 100 mV increase in potential was observed over the period from 35 to 60 min (Figure S8A), in contrast to the roughly constant potential observed over the same period for a GC WE. Significant Au deposition was observed on the Pt mesh after 60 min (Figure S8D). The higher surface area of the Pt mesh, as opposed to a Pt wire WE, means that significant Au coverage of the electrode via deposition takes longer, and thus the measured steady state OCP initially matches the measurement with a GC WE. As Au eventually plates on the Pt mesh after ∼35 min of reaction, the potential starts to slowly and then rapidly shift more positive as the Au coverage of the WE increases. ORP sensors with glassy carbon WEs are not presently commercially available, but ORP sensors with a variety of sizes and geometries of noble metal WEs are sold. These data indicate that use of an ORP sensor with a larger Pt WE than the SJ epoxy ORP sensor could improve agreement between ORP sensor and potentiostat OCP measurements of Au growth solutions for reactions which only need to be measured for short time periods (30 min or less).

To address issues of both Ag^+^ ion leaching from the RE and rapid Au fouling of the Pt wire WE, an ORP sensor from Cole-Parmer with a glass body, a larger Pt band WE (∼97 mm^2^ SA) and a double junction (DJ) separating the Ag/AgCl reference electrode from solution was tested. This sensor will be referred to as “DJ glass ORP.” Measurements of Au nanoparticle growth with a DJ glass ORP sensor matched well with the potentiostat OCP measurements taken with an Ag/AgCl RE and GC WE (Figure 4) after the first 5 min of measurement and demonstrated ±10 mV measurement-to-measurement consistency. These measurements were still shifted ∼100 mV more positive in potential than potentiostat OCP measurements taken with an Hg/Hg_2_SO_4_ RE, indicative of some low level of Ag^+^ leakage from the DJ glass ORP sensor (likely ≤230 nM Ag^+^, Figure S5), despite the additional junction before the RE. SEM images also confirmed a similar appearance of the Au particles measured with a DJ glass ORP sensor to those measured with SJ epoxy ORP or potentiostat OCP (Figure S9 and Table S3). Although some minor Au deposition on the DJ glass ORP sensor body was visible following 30 min of measurement, the Pt band visually appeared mostly clean of Au (Figure S10). Measurement of Au particle growth for longer than 27 to 31 min with the DJ glass ORP sensor led to an eventual increase in potential, similar to potentiostat OCP measurements with a Pt mesh WE (Figure S11A), indicating that this ORP sensor type is prone to WE fouling after ∼30 min in Au growth solutions. A protocol for aqua regia cleaning (1:3 ratio of concentrated HNO_3_: concentrated HCl; **caution: strong acid**) of the DJ glass ORP sensor between each use to remove deposited Au from the Pt band was crucial for accurate measurement.

DJ glass ORP measurements of the corrugated Pd particle synthesis in Sigma CTAC were also taken to confirm a lack of influence of the ORP sensor type—due to the different body materials and different electrode geometries—on OCP measurements or morphological results of Pd particle synthesis. The DJ glass ORP measurements and corresponding product morphologies were equivalent to those measured with a SJ epoxy ORP sensor (Figure S12). The Pd THH synthesis was also measured with the DJ glass ORP sensor to probe its sensitivity and responsiveness at early time points (Figure S13 and Table S2). Like the SJ epoxy ORP sensor, the DJ glass ORP sensor failed to capture the initial dip in potential associated with reagent diffusion and a change in growth rate observed in potentiostat OCP measurements. This is unsurprising, since the working electrode in both cases is oxidized Pt.^44^ After ∼10 min of measurement, the DJ glass ORP measurements matched the potentiostat OCP benchmark well (±5 mV) and did not affect the formation of the THH particle shape (Figure S13).

Our group’s recent work has presented potentiostat OCP measurements as a method for facile deconvolution of the relative contributions of surface passivation vs reduction kinetics to shape control during metal nanoparticle growth.^44^ To establish the ability of ORP sensor measurements to distinguish between these mechanisms, we used a DJ glass ORP sensor to monitor Au growth solutions with varying concentrations of dilute Ag^+^ (0.92–92 μM). Addition of Ag^+^ to the Au growth solution induces shape control via an underpotential deposition (UPD) surface passivation mechanism to produce an [Ag^+^]-dependent shape gradient of (Ag)Au octahedra, rhombic dodecahedra, truncated ditetragonal prisms, and concave cubes.^61,62,70–72^ As shape is entirely controlled by Ag UPD surface passivation across the full range of Ag^+^ concentrations, these syntheses have identical metal ion reduction kinetics^11^ and, therefore, very similar (±10 mV) mixed solution potentials when measured by potentiostat OCP (GC WE vs Hg/Hg_2_SO_4_ RE).^44^ Indeed, when the OCP of the synthesis for each (Ag)Au shape was measured with a DJ glass ORP sensor, the potentials fell within a ±20 mV range, with no particular ordering of potentials related to Ag^+^ concentration (Figure 5A). For triplicate measurements of each Ag^+^ concentration, SEM images demonstrated formation of the correct shape (Figure 5B–E) in the presence of the DJ glass ORP sensor, indicating that the presence of the sensor does not perturb shape formation, despite the leakage of nanomolar concentrations of Ag^+^ ions from the sensor’s RE.

**Figure 5:**
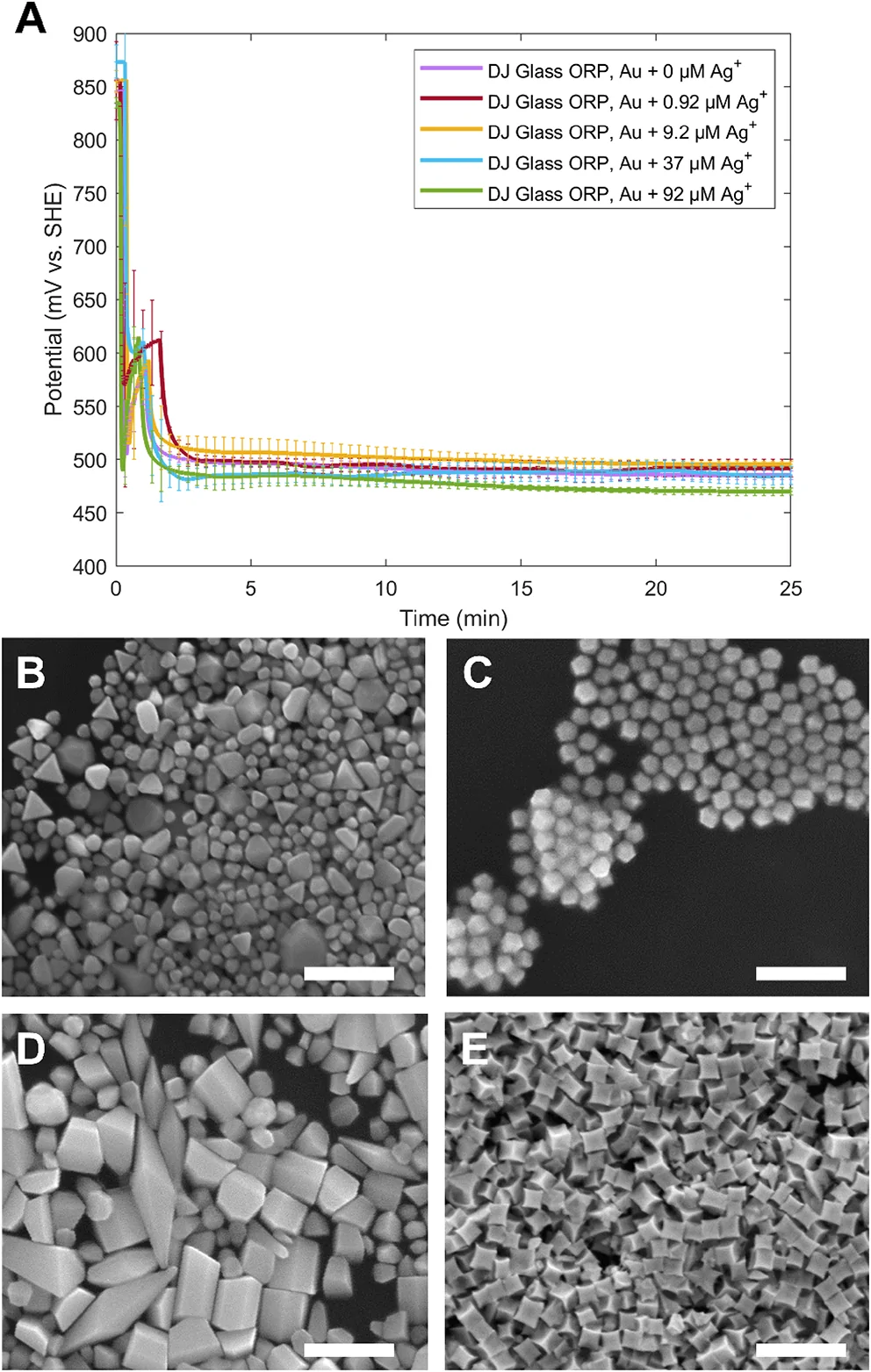
(A) DJ glass ORP sensor measurements (Pt band WE vs Ag/AgCl RE) of Au particle synthesis with different concentrations of Ag^+^ added for surface passivation-based shape direction. Error bars represent the standard deviation of three measurements. (B–E) SEM images of Au nanoparticle shape as a function of Ag^+^ concentration in the growth solution: (B) 0.92 μM (octahedra, red trace), (C) 9.2 μM (rhombic dodecahedra, yellow trace), (D) 37 μM (truncated ditetragonal prisms, blue trace), and (E) 92 μM (concave cubes, green trace). Scale bars: 500 nm.

The experiments in this section demonstrate that, with appropriate probe choice, ORP probes can be used as an alternative to potentiostat OCP for reliable and accurate measurements of monometallic Au and (Ag)Au bimetallic nanoparticle growth. Further discussion of best practices for choosing an ORP sensor for different reaction conditions is provided in the Supporting Information and in Table S1.

### 2.3 Development of Protocols for OCP and ORP Measurements of Copper Nanoparticle Growth

While Au and Pd historically have been the most studied elements in metal nanoparticle syntheses due to their relatively positive reduction potentials and resulting ease of nanoparticle synthesis at room temperature with weak reducing agents, there is an increased focus on the shape-selective synthesis of nanoparticles consisting of non-noble metals such as Cu, cobalt (Co) and nickel (Ni) owing to their abundance and prospective uses in energy and catalysis applications.^73–75^ Given the emerging nature of non-noble metal nanoparticle synthesis, there is a particular gap in synthetic troubleshooting or mechanistic information for these metal compositions. For this reason, we wanted to investigate the viability of measuring the OCP of an aqueous Cu nanoparticle synthesis, using a traditional potentiostat and/or ORP sensors.

In general, the less noble character of Cu demands a stronger reducing environment to induce metal nanoparticle growth, resulting in the need for increased temperature and/or higher concentrations of reducing agent in aqueous syntheses of Cu nanoparticles compared to conditions commonly found in the synthesis of Pd and Au nanoparticles.^21,76,77^ The increased temperature complicates in situ OCP measurements of Cu particle synthesis by introducing additional variables, including the initial temperature ramp of the reaction and temperature-related electrode stability issues. Slight fluctuations in the initial temperature ramp have been shown to influence the outcome of Cu nanoparticle growth.^78,79^ This warrants particular consideration, because adding the electrodes necessary to measure the OCP can alter this temperature ramp from the typical temperature ramp for the unmeasured synthesis. Furthermore, the potential of REs—and, therefore, the measured OCP itself—is temperature-dependent, which requires consideration when interpreting and comparing the OCP of different reaction conditions.^45,80^ Additionally, the increased temperatures pose a challenge for the use of ORP sensors, as the working range for a wide variety of commercially available ORP sensors lies below the ≥90 °C utilized in colloidal aqueous synthesis of Cu nanoparticles. Paired with the strongly reducing reaction environment, this presents concerns about the durability of the ORP sensors’ electrodes in measuring the Cu nanoparticle growth. With these considerations in mind, we sought to develop an appropriate and reproducible protocol for both the potentiostat OCP and ORP sensor measurements of typical high-temperature aqueous Cu nanoparticle synthesis conditions.

A synthesis for cubic Cu nanoparticles in CTAC with sodium ascorbate (NaAsc) as the reducing agent^75^ was chosen as a model system to test the feasibility of these in situ potentiostat OCP and ORP sensor measurements of growth due to the similarity of these reagents to those used in the Au and Pd syntheses described above. In the selected model system (referred to as the “CTAC/NaAsc” Cu nanocube synthesis), a growth solution of copper­(II) acetate (Cu­(OAc)_2_) in 15 mM Sigma CTAC was prepared. Growth was initiated with the addition of NaAsc, and the growth solution was stirred at 750 rpm at room temperature for 1 min before heating to 90 °C with minimal stirring for 40 min.^75^

Establishing a potentiostat OCP measurement of the CTAC/NaAsc Cu cube synthesis as a benchmark to compare to ORP sensor measurements immediately highlighted the challenges in adapting the OCP method to syntheses of non-noble metal nanoparticles. The potentiostat OCP measurement setup previously used for the measurements of Pd and Au nanoparticle growth, including a 5-neck electrochemistry flask and a glass bridge tube for the RE, resulted in the formation of Cu nanoparticles with undefined shapes due to detrimental glassware sensitivity. Attempts to troubleshoot the glassware sensitivity by taking potentiostat OCP measurements directly in the glass scintillation vials typically used for the Cu cube reaction, with a GCE as the WE and a Ag/AgCl RE in a PTFE bridge tube to remove additional sources of glass, still led to the formation of Cu nanoparticles with an undefined shape. This was hypothesized to be a result of the leaching of Ag^+^ from the Ag/AgCl RE into the growth solution, as was previously observed via OCP measurements during monometallic Au nanoparticle synthesis. This sensitivity to glassware and Ag^+^ ions was not specific to the CTAC/NaAsc Cu nanocube synthesis described above; another cubic Cu nanoparticle synthesis with a growth solution composed of hexadecylamine and glucose^81^ showed similar disruption of growth.

To overcome this challenge, a reversible hydrogen electrode (RHE) (HydroFlex, Gaskatel GmbH) was used as the RE for potentiostat OCP measurements. This electrode is Ag-free, has a PTFE body, and fits directly in the scintillation vials used for the colloidal synthesis, eliminating both detrimental factors (Ag^+^ leaching and glassware sensitivity) identified in the initial experiments. Because the RHE is used directly in the growth solution—rather than being separated by a bridge tube—and the measured potential of the RHE is pH- and temperature-dependent, the pH of the solution and the temperature profile of the reaction throughout the growth were monitored (Figure S14). To avoid the use of a pH probe with Ag-containing electrodes, the pH was monitored using an RHE-based pH sensor (pHydrunio, Gaskatel GmbH). Additionally, the temperature of the oil bath used to heat the reaction vial during the synthesis had to be increased from 90 to 100 °C when measuring the OCP to yield a higher portion of well-formed cubic Cu nanoparticles. We hypothesize that a temperature increase is necessary because the presence of the GCE and RHE electrodes decreases the overall growth solution temperature compared to the unmeasured control. With a GC WE and RHE RE, potentiostat OCP measurements of the CTAC/NaAsc Cu model system were reproducible (Figure 6A) and the particles formed while measuring the OCP were predominantly cubes with a subpopulation of planar twinned bipyramids (Figure 6B). The particle morphologies produced in the measured reaction were generally similar to products of the synthesis without OCP measurement (Figure S15), although there were an increased number of nanowires as a side product when the OCP was measured using the RHE (Figure S16A–B).

**Figure 6:**
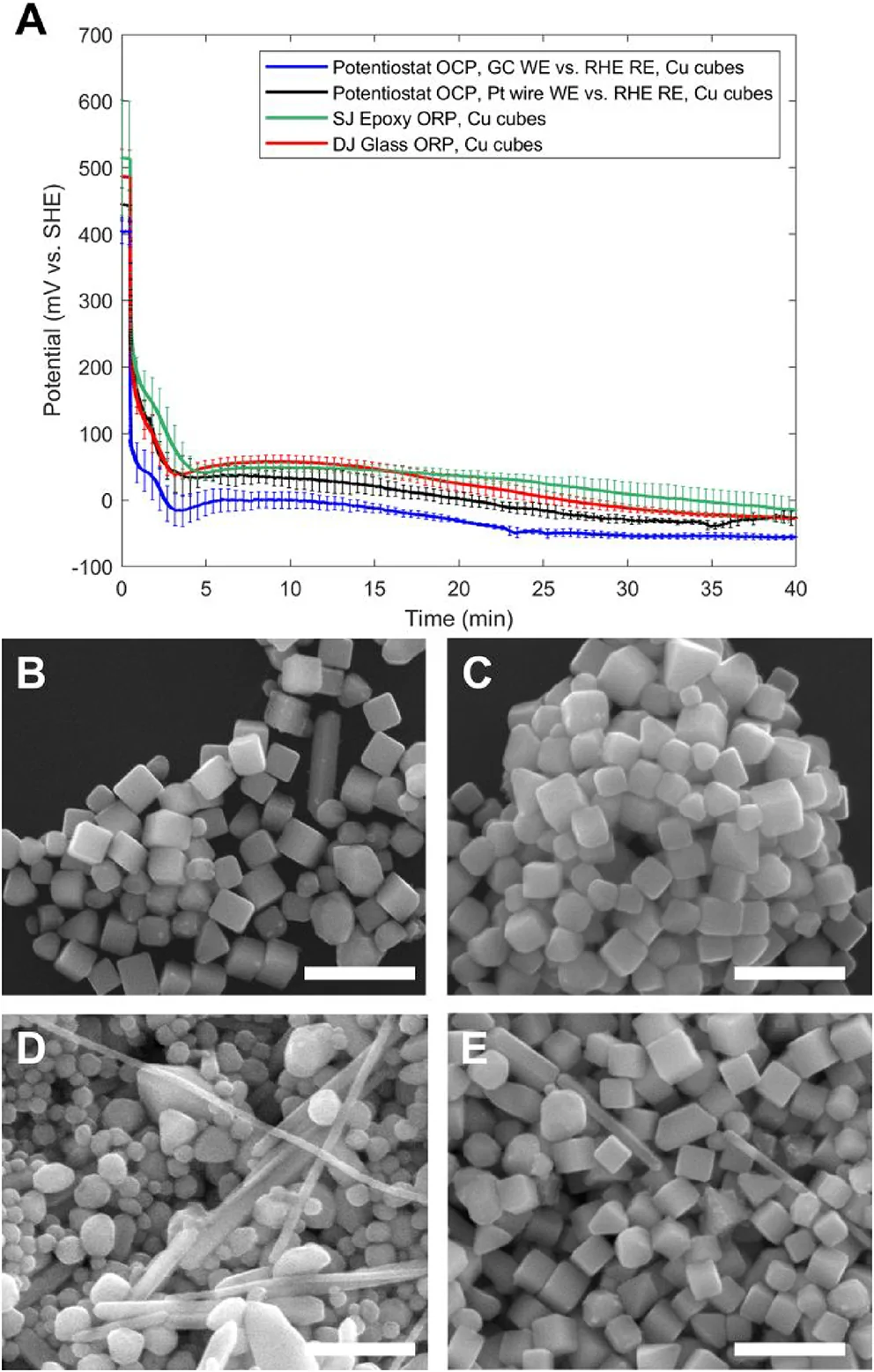
(A) Comparison between OCP measurements of the CTAC/NaAsc Cu nanocube synthesis taken with potentiostat OCP using a GC WE vs a RHE RE (blue), potentiostat OCP using a Pt wire vs a RHE RE (black), a SJ epoxy ORP sensor (Pt band WE vs Ag/AgCl RE; green), and a DJ glass ORP sensor (Pt band WE vs Ag/AgCl RE; red). Error bars represent the standard deviation of three measurements. (B-E) SEM images of CTAC/NaAsc Cu nanocubes synthesized during OCP measurement with (B) potentiostat OCP using a GC WE vs a RHE RE; (C) potentiostat OCP using a Pt wire WE vs a RHE RE; (D) a SJ epoxy ORP sensor (Pt band WE vs Ag/AgCl RE); and (E) a DJ glass ORP sensor (Pt band WE vs Ag/AgCl RE). Scale bars: 500 nm.

Of note, these potentiostat OCP measurements, reported vs SHE with temperature and pH correction, showed a drop in potential to around +40 mV vs SHE following the addition of the NaAsc, and then a further decrease to approximately −15 mV vs SHE when the reaction vial was heated to 100 °C (Figures 6A and S17), indicating the influence of the heating ramp on the measured OCP. In general, the potentiostat OCP traces measured against the RHE showed a slightly higher measurement-to-measurement variance than the potential traces measured for Pd and Au against the Ag/AgCl or Hg/Hg_2_SO_4_ electrodes. This may be attributable to slight differences in the temperature gradient between the replicate measurements of the CTAC/NaAsc Cu nanocube synthesis. Increased measurement-to-measurement variability could also be a factor of the RHE itself, since the reference potential of the RHE is based on flowing gas over an electrode, which is an inherently less stable system than traditional REs.

Since we had observed a dependence of the measured mixed potential on the WE material in OCP measurements of Au nanoparticle synthesis, a Pt wire was investigated as the WE for potentiostat OCP measurements of the Cu cube synthesis to facilitate later comparison to ORP sensors with a Pt WE. Similarly to the Au OCP measurements, the potentiostat OCP was shifted to more positive potentials (a 35–60 mV increase, depending on the progression of the reaction) when using a Pt wire as the WE instead of a GCE, after initially measuring at a similar potential prior to the addition of the reducing agent. Cu deposition was not visually notable on the Pt WE. The shift in potentiostat OCP when changing from a GCE to a Pt WE in the case of the Cu model system might be explained by the influence of WE material on the ease of ET, as previously discussed. While the exact species catalyzed by the Pt WE compared to the GC WE is nontrivial to determine, Pt nanoparticles have been shown to catalyze the oxidation of NaAsc,^60^ and thus facilitated ET between NaAsc and the Pt WE might cause the observed shift in the measured mixed solution potential when a Pt WE is used rather than a GC WE. A shift in the measured OCP on Pt-containing WE materials, as compared to other WE materials, has also been associated with catalysis of the oxygen reduction reaction (ORR) in the literature, which may be a significant contributor to the OCP due to the stronger reducing environment used in the Cu cube synthesis compared to the previously investigated Pd and Au nanoparticle syntheses.^59^

Interestingly, approximately 35 min after the addition of NaAsc, the measured potentiostat OCP with the Pt wire WE started to increase again until the end of the measurement was reached, a behavior not noted with the glassy carbon WE. The particles that formed, similarly to the measurement using a GC WE, were predominantly well-faceted cubes with subpopulations of bipyramids, nanowires, smaller rounded cubes and some bars (Figures 6C and S16C–D). The increase in the measured mixed solution potential after around ∼35 min is hypothesized to occur due to the contribution from oxidative degradation of NaAsc starting to dominate the mixed potential, as opposed to contributions from metal ion reduction—a phenomenon previously noted for AA in potentiostat OCP measurements of Pd particle synthesis.^44^ The OCP of a solution containing Sigma CTAC and NaAsc, similar to the growth solution used for Cu cube growth but without the Cu precursor, remained constant at around +335 mV vs RHE after 30 min of the reaction, when measured using a GC WE, which is comparable to the potential measured with the GC WE at the conclusion of the Cu nanoparticle growth reaction. On the other hand, measuring the Cu-free solution with a Pt-wire WE led to a constant potential of about +400 mV vs RHE starting 10 min into the reaction (Figure S18). This might explain the upward trend in the solution potential toward this value after conclusion of the Cu nanoparticle growth for the measurement of Cu nanoparticle growth using the Pt wire WE.

After establishing the benchmark potentiostat OCP measurements with a Pt WE, the use of ORP sensors to measure the OCP of the Cu model system was investigated. The use of ORP sensors allows for the omission of pH and temperature measurements of the solution during the OCP measurement while also avoiding glassware sensitivity issues, minimizing the experimental complexity and expense compared to the potentiostat OCP measurements with the RHE. As discussed previously, the reported working temperature range of most commercially available ORP sensors lies below the 90–100 °C temperatures of this Cu cube synthesis. Nonetheless, we initially investigated the viability of the SJ epoxy ORP sensor (working range of 0–60 °C) in measuring the OCP trace of the reaction. The measured potential trace is relatively similar in shape and potential to the potentiostat OCP trace with a Pt wire WE, although it shows a shoulder in the potential drop immediately after the addition of NaAsc (Figure 6A). The slightly increased potential between 5 and 20 min after the addition of the NaAsc can be attributed to the fact that the ORP sensor studies were carried out at 90 °C. While the measured potential trace for the SJ epoxy ORP sensor overall is relatively similar to the potentiostat OCP measurement, there is only a minimal formation of cubes with a majority of large, undefined shapes and rods (Figure 6D). The loss of shape selectivity is hypothesized to occur due to the leaching of Ag^+^ ions from the SJ epoxy ORP sensor, with the high reaction temperature likely increasing leaching of Ag^+^ into the growth solution. Ag^+^ leaching could not be detected via ICP-OES analysis of the nanoparticle growth solution following synthesis and measurement, indicating that the amount of leached Ag^+^ is beneath the 1 ppm detection limit of the ICP-OES instrument (equal to ≤9.3 μM Ag^+^; ≤930 nM Ag^+^). When low amounts of Ag^+^ were intentionally added to unmeasured growth solutions prior to the initiation of metal ion reduction, Ag^+^ concentrations as low as 0.01 ppm (equal to 92.7 nM Ag^+^) led to the formation of poorly defined particles (Figure S19), which were similar in shape to the particles produced during the SJ epoxy ORP measurements (Figure 6D).

The DJ glass ORP introduced in Section 2.2 was tested next, as it has an expanded working range of −5 to 100 °C and the DJ sufficiently minimized the leaching of Ag^+^ into Au growth solutions to prevent changes to product morphology. Initial use of the DJ glass ORP sensor during the reactions at 90 °C led to well-formed cubes, without nanowires as a side product, similar to the potentiostat OCP measurements (Figure 6A,E). While initially promising, the DJ glass ORP sensor began to lead to the formation of undefined Cu nanoparticles after ≥10 uses (Figure S20F). This again is hypothesized to be due to the leaching of Ag^+^ from the ORP sensor’s RE, possibly accelerated by repeated cleaning of the sensor using conc. HNO_3_ (**caution: strong acid**). The potential trace measured by the fresh DJ glass ORP sensor, with cubic Cu nanoparticles as the main product, agreed well with the potentiostat OCP measured with RHE and a Pt wire WE—only differing in potential between 5 and 20 min after the addition of the NaAsc, similar to the SJ epoxy ORP traces. These differences in potential were minimized if the temperature was increased to 100 °C for the DJ glass ORP (Figure S20A,B), indicating that the differences between the potentiostat OCP and ORP sensor potential traces can be likely attributed to a difference in solution temperature rather than a fundamental chemical difference. Of note, the DJ glass ORP measurements at 100 °C were conducted with a well-used ORP sensor, which led to the formation of noncubic nanoparticles due to Ag^+^ leaching.

Interestingly, the DJ glass ORP measurement at 100 °C, as well as the SJ epoxy ORP measurements, which both lead to a loss of shape selectivity, had a shoulder in the initial potential drop after the addition of the NaAsc (Figures 6 and S20A–B). This shoulder and loss of shape selectivity were also observed if the potentiostat OCP was measured with a Ag/AgCl RE (Figure S21). To probe whether this observed shoulder is connected to the hypothesized loss of shape selectivity introduced by Ag^+^ leaching, the potentiostat OCP of the reaction with deliberate addition of ∼100 nM Ag^+^ was measured using the Pt wire WE vs RHE RE. The resulting OCP trace showed a shoulder in the initial potential drop after the addition of the reducing agent (Figure S20A–B), similar to the OCP trace measured by the DJ glass ORP, and this Ag^+^ addition also led to a loss of shape selectivity of the particles (Figure S20D). This indicates that Ag^+^ leaching is indeed responsible for the loss of shape selectivity. From our previous studies on the OCP of Pd and (Ag)Au nanoparticle growth, we note that only kinetic changes in nanoparticle growth are detectable via differences in the measured OCP, while purely surface passivation effects—which do not alter growth kinetics—do not cause changes in the OCP.^44^ As a significant shoulder is visible in the measured potentiostat OCP with the deliberately added Ag^+^ (as well as in OCP measurements with a well-used DJ glass ORP, which is presumed to leach Ag^+^) this indicates an effect of the Ag^+^ on the growth kinetics immediately after the addition of the NaAsc to the solution. Prior studies of Cu nanoparticle growth have suggested an important role of the initial seeding step in determining the shape of the resulting Cu nanoparticles.^77,78,82^ This indicates that the mechanism by which the Ag^+^ introduces the loss of shape selectivity is likely through a change of the growth kinetics in the seeding step and early growth, leading to a change in the number and/or shape of the seeds produced and their initial development.

To evaluate the viability of using ORP sensors for the measurement of the OCP of Cu nanoparticle syntheses more broadly, and to isolate influence of Ag^+^ leaching on seed formation vs growth, we investigated a Pd cube-seeded synthesis for cubic Cu nanoparticles using AA as the reducing agent and hexadecylamine (HDA) as the capping agent (referred to as the “HDA/AA” Cu nanocube synthesis). In control experiments with deliberately added AgNO_3_, the reaction showed increased resistance to Ag^+^-induced shape change compared to the previously studied unseeded synthesis in CTAC (Figure S22). Establishing the potentiostat OCP benchmark in this case was possible with a Ag/AgCl RE directly inserted into the growth solution, eliminating the need for a bridge tube. The potentiostat OCP was measured using both a GCE and Pt wire, separately, as the WE. Using Pt as the WE resulted in a significantly more positive OCP than with a GC WE (between 90 and 35 mV more positive at different points in the reaction, with the difference decreasing in magnitude over time). The two OCP traces also had significantly different lineshapes early in the reaction (Figures 7A and S23). Regardless, in both cases, the resulting particles were well-formed cubes with minor populations of bipyramids and nanowires as a side product (Figure 7B–C). The general difference in the OCP traces can be explained by the difference in the ET, depending on the WE material. The initial jump in potential after the addition of the AA, only seen when using a Pt WE, is attributed to the nonideal electrode response of the Pt WE to the significant [AA] changes due to rapid reduction of Cu^2+^. This is caused by a Pt oxide species on the Pt wire and is not indicative of the nanoparticle growth chemistry.

**Figure 7:**
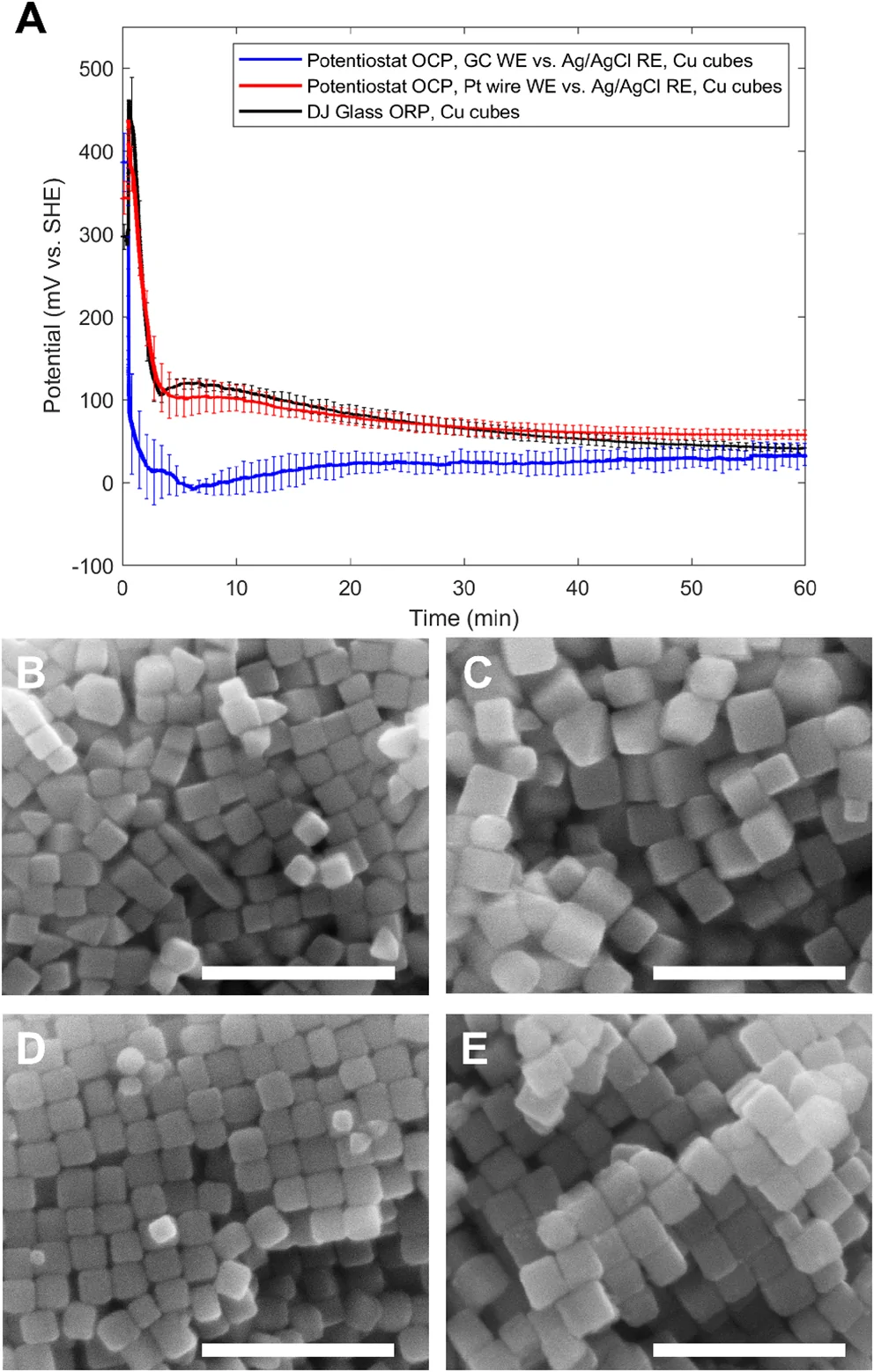
(A) Comparison between OCP measurements of the Pd-seeded HDA/AA Cu nanocube synthesis taken with potentiostat OCP using a GC WE vs a Ag/AgCl RE (blue), potentiostat OCP using a Pt wire WE vs a Ag/AgCl RE (red), and a DJ glass ORP sensor (Pt band WE vs Ag/AgCl RE; black). Error bars represent the standard deviation of three measurements. (B–E) SEM images of Pd-seeded HDA/AA Cu nanocubes measured during synthesis with (B) potentiostat OCP using a GC WE vs a Ag/AgCl RE, (C) potentiostat OCP using a Pt wire WE vs a Ag/AgCl RE, (D) a DJ glass ORP sensor (Pt band WE vs Ag/AgCl RE), and (E) unmeasured. Scale bars: 500 nm.

When OCP measurements of this Pd-cube-seeded Cu nanoparticle synthesis were taken with a DJ glass ORP sensor, the reaction produced well-formed cubic Cu nanoparticles (Figure 7D), similar to the particles formed in both the unmeasured control (Figure 7E) and the potentiostat OCP setup with the Ag/AgCl RE directly inserted into the growth solution (Figure 7B–C). The potential measured with the DJ glass ORP sensor matched the OCP measured with a potentiostat and Pt wire WE, except for ∼30 mV shifts before the addition of the reducing agent and ∼35 min after the addition of the AA (Figure 7A). These data support our hypothesis that the reason for the loss of shape selectivity during the CTAC/NaAsc synthesis was a change in the reduction kinetics during the seeding step of the synthesis, due to interference from Ag^+^ ions. The HDA/AA system is more resistant to the influence of Ag^+^ in the seed formation and early growth steps due to the use of preformed seeds. The increased concentrations of reducing agent and Cu^2+^ in the HDA/AA growth solution compared to the CTAC/NaAsc growth solution (20.5 mM AA vs 8.5 mM NaAsc; ∼5.2 mM vs ∼0.8 mM Cu^2+^) might also decrease the influence of changes in the initial growth kinetics introduced by Ag^+^ in the seeded reaction, as the relative impact on the growth kinetics introduced by low-concentration Ag^+^ leaching might be smaller.

Taken together, these data demonstrate proof-of-concept for the use of ORP sensors to measure the OCP of non-noble metal nanoparticle growth systems. Additional challenges of potentiostat OCP and ORP sensor measurements of non-noble metal syntheses, including the need to consider the effects of elevated temperature on both electrode stability and the electroanalytical measurements themselves, as well as the increased sensitivity to glassware and electrode materials, were also identified. Detailed information regarding best practices for ORP sensor measurements of non-noble metal nanoparticle synthesis is provided in the Supporting Information.

### 2.4 Using ORP Sensors to Troubleshoot the Presence of Impurities in CTAC

Recent work reported that CTAC from several different manufacturers and lines had different levels of iodide impurities—a previously known contaminant in CTAB^27–29,42^ —but also appeared to have an additional unknown contaminant that was responsible for the majority of Au particle shape direction arising from changes in the CTAC source.^83^ In addition, the (Ag)Au syntheses for octahedra, rhombic dodecahedra, truncated ditetragonal prisms, and concave cubes discussed in Section 2.2 are unsuccessful in CTAC purchased in a powdered form from Tokyo Chemical Industries (“TCI CTAC”; 98%). More generally, we have observed that many Pd and Au nanoparticle syntheses developed in Sigma CTAC are unsuccessful in TCI CTAC and vice versa. Building on our prior work using potentiostat OCP measurements to troubleshoot impurity variations across different CTAB manufacturers,^24^ we investigated whether ORP sensors can likewise be used to troubleshoot impurities in CTAC.

When growth solutions for bimetallic (Ag)Au particles were prepared in TCI CTAC with the standard amounts of Ag^+^ (0.92 μM, 9.2 μM, 37 μM, and 92 μM AgNO_3_), the products were shifted toward shapes that would be predicted to form at higher concentrations of added Ag^+^, and thus higher surface coverages of Ag (Figure 8). Addition of 0.92 μM Ag^+^ generated {110}-faceted rhombic dodecahedra and bipyramids rather than octahedra; 9.2 μM Ag^+^ led to formation of concave cubes of mixed sizes and poorly formed formed small particles rather than rhombic dodecahedra; 37 μM Ag^+^ led to well-formed concave cubes rather than truncated ditetragonal prisms; and 92 μM Ag^+^ generated mostly spiked particles with some planar twinned concave cube derivatives and a small number of triangular plates (Figure 8B–E). The solution potentials for all four Ag^+^ conditions in TCI CTAC, measured with a DJ glass ORP sensor, were 75 ± 25 mV more positive than measurements of otherwise identical growth solutions in Sigma CTAC (Figure 8A), yet remained within ±15 mV of one another, with no ordering of potentials based on [Ag^+^]. The shift to a more positive OCP (weaker reducing environment) for all growth conditions in TCI CTAC compared to Sigma CTAC indicates a difference in Au reduction kinetics, either overall or in the early minutes of metal ion reduction.^44^ The similarity of ORP measurements across all concentrations of Ag^+^, meanwhile, indicates that the mechanism of shape control in TCI CTAC remains surface passivation of Au by Ag UPD.

**Figure 8:**
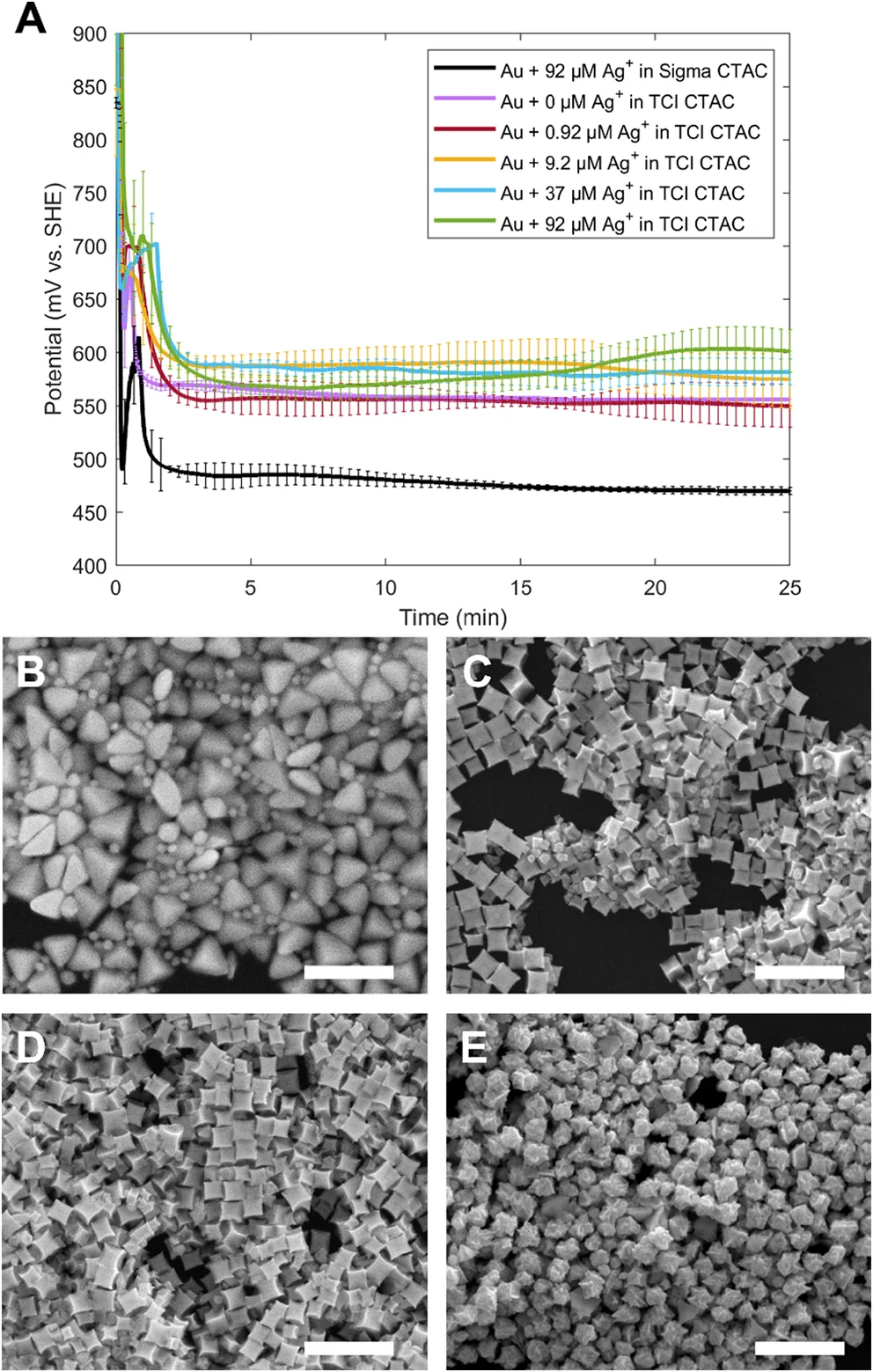
(A) Comparison between DJ glass ORP measurements (Pt band WE vs Ag/AgCl RE) of Au particle synthesis in as-received TCI CTAC with different concentrations of Ag^+^ added for surface passivation-based shape direction (purple, red, yellow, blue, green) and Au concave cube synthesis with 92 μM AgNO_3_ in as-received Sigma CTAC (black). Error bars represent the standard deviation of three measurements. (B–E) SEM images of Au nanoparticle shape as a function of Ag^+^ concentration: (B) 0.92 μM (rhombic dodecahedra and bipyramids, red trace), (C) 9.2 μM (concave cubes, yellow trace), (D) 37 μM (concave cubes, blue trace), and (E) 92 μM AgNO_3_ (spiked particles, green trace) in the growth solution. Scale bars: 500 nm.

The shifting of (Ag)Au nanoparticle morphology to shapes with higher expected surface coverages of Ag for a given [Ag^+^] in the growth solution has previously been observed in the presence of dilute halides, especially iodide, and can be attributed to an enhancement of the rate of reduction of Ag relative to Au and/or to the destabilization of the Ag UPD layer.^11,84^ The possibility of increased iodide contamination in TCI CTAC relative to Sigma CTAC is further supported by our prior observation that high nanomolar/low micromolar iodide concentrations—either as an intentional additive or a contaminant in CTAB—raised the measured solution potential in potentiostat OCP measurements of Pd particle synthesis.^24^ To estimate the difference in iodide concentration in TCI CTAC vs Sigma CTAC, sodium iodide (NaI) was added to Sigma CTAC for the synthesis with 92 μM Ag^+^. Addition of ≤0.15 μM NaI led to overgrown concave cubes, and higher concentrations of NaI led to the formation of an increasing percentage of stellated particles (Figure S24). The condition with 0.19 μM NaI added to Sigma CTAC most closely resembled the mixture of particles formed in TCI CTAC. The OCP for this condition taken with a DJ glass ORP sensor was quite similar to measurements of (Ag)Au particle synthesis in as-received TCI CTAC (within 10 mV), supporting the likelihood of this level of iodide contamination in the TCI CTAC.

To enable direct control of iodide and other inorganic ion contaminant concentrations in TCI CTAC, the powder was recrystallized and oven-dried. The purity of the recrystallized product was confirmed by NMR (^1^H NMR, 600 MHz, D_2_O; NMR data available in SI; Figures S25–S26). (Ag)Au particle growth solutions prepared in recrystallized TCI CTAC with 92 μM Ag^+^—which would yield concave cubes in Sigma CTAC—produced a mixture of truncated ditetragonal prisms, shard-like particles, and a few unusually large concave cube particles (Figure S27B), indicative of a lower Ag surface coverage on the Au particles than expected. DJ glass ORP measurements of this synthesis condition were even less positive than measurements of the same condition in as-received Sigma CTAC (Figure S27A). These results suggest that a low concentration of iodide is present in the Sigma CTAC and is required to match the Au reduction kinetics to the (Ag)Au concave cube OCP benchmark. However, addition of a gradient of NaI concentrations to Au particle growth solutions with 92 μM Ag^+^ in recrystallized TCI CTAC improved morphology but did not produce high-quality concave cubes at any concentration (Figure S28A–C), indicating that another critical shape-directing impurity is also present. Addition of 0.39 μM NaI, the only iodide concentration which caused production of some concave cubes, led to an ORP trace which was shifted 10–25 mV more positive (depending on the degree of reaction completion) compared to the optimal as-received Sigma CTAC benchmark (Figure S28D).

Based on our previous work troubleshooting line-to-line and lot-to-lot CTAB differences in the kinetically controlled synthesis of Pd THH, adventitious organic small molecules (acetone; short-chain alcohols) cocrystallized with powdered commercial surfactants have rate- and shape-directing properties which can be leveraged synergistically with iodide to provide finer synthetic control.^24^ Changes in metal ion reduction kinetics likely occur due to the intercalation of small organics in the surfactant bilayer at the growing particle surface—disturbing the packing of the bilayer.^24,85,86^ These changes in reduction kinetics are observable as shifts in the measured potentiostat OCP.^24,44^ Organic solvent contaminants were considered next as a synthetic handle, in combination with iodide, for troubleshooting the difference between Sigma and TCI CTAC. The presence of a possible organic solvent contaminant was further supported by an impurity peak at 2.90 ppm in the ^1^H NMR spectrum of as-received Sigma CTAC (600 MHz, D_2_O; NMR data available in SI; Figure S29) which does not appear in the ^1^H NMR spectrum of as-received TCI CTAC (Figure S25), though this peak could not be assigned conclusively.

To better capture possible kinetic changes resulting from low-concentration organic solvent impurities, we probed the Pd corrugated particle synthesis, which was also developed in as-received Sigma CTAC, and is a system where shape is more kinetically controlled than it is in the UPD-driven (Ag)Au particle syntheses. By modifying the CTAC source and additive concentrations in the Pd corrugated particle synthesis, we were able to note changes in the ORP sensor measurements more easily due to the stronger influence of additives on reaction kinetics and then apply that information to the (Ag)Au synthetic system. When Pd corrugated particles were synthesized in as-received TCI CTAC, their morphology was poorly defined and surface corrugation less pronounced, and they were generally larger than particles synthesized in Sigma CTAC (Figures 9B and S30A–C). SJ epoxy ORP measurements indicated that the mixed potential of corrugated particle growth in TCI CTAC is shifted 80 ± 10 mV more positive (less strongly reducing), compared to the benchmark measurements in Sigma CTAC (Figure 9A). The mixed potential when the recrystallized TCI CTAC was used instead was less positive than measurements in both as-received Sigma CTAC and as-received TCI CTAC (Figure 9A) which again suggests that a small amount of iodide is necessary in the growth solution to match the optimal reduction kinetics observed in as-received Sigma CTAC. TCI CTAC powder that was only oven-dried but not recrystallized before use in the Pd corrugated particle synthesis led to similar rounded, poorly faceted corrugated particle products and a similar solution potential to ORP sensor measurements in as-received TCI CTAC, indicating that as-received TCI CTAC powder likely contains little or no solvent contamination (Figure S31). From this set of experiments, we can conclude that (1) some low threshold concentration of iodide is necessary for the growth kinetics in this synthesis; (2) this level of iodide is present in as-received Sigma CTAC; and (3) if an organic solvent is indeed the secondary contaminant, it is beneficial to shape formation in this synthesis and present in higher concentrations in Sigma CTAC than in TCI CTAC.

**Figure 9:**
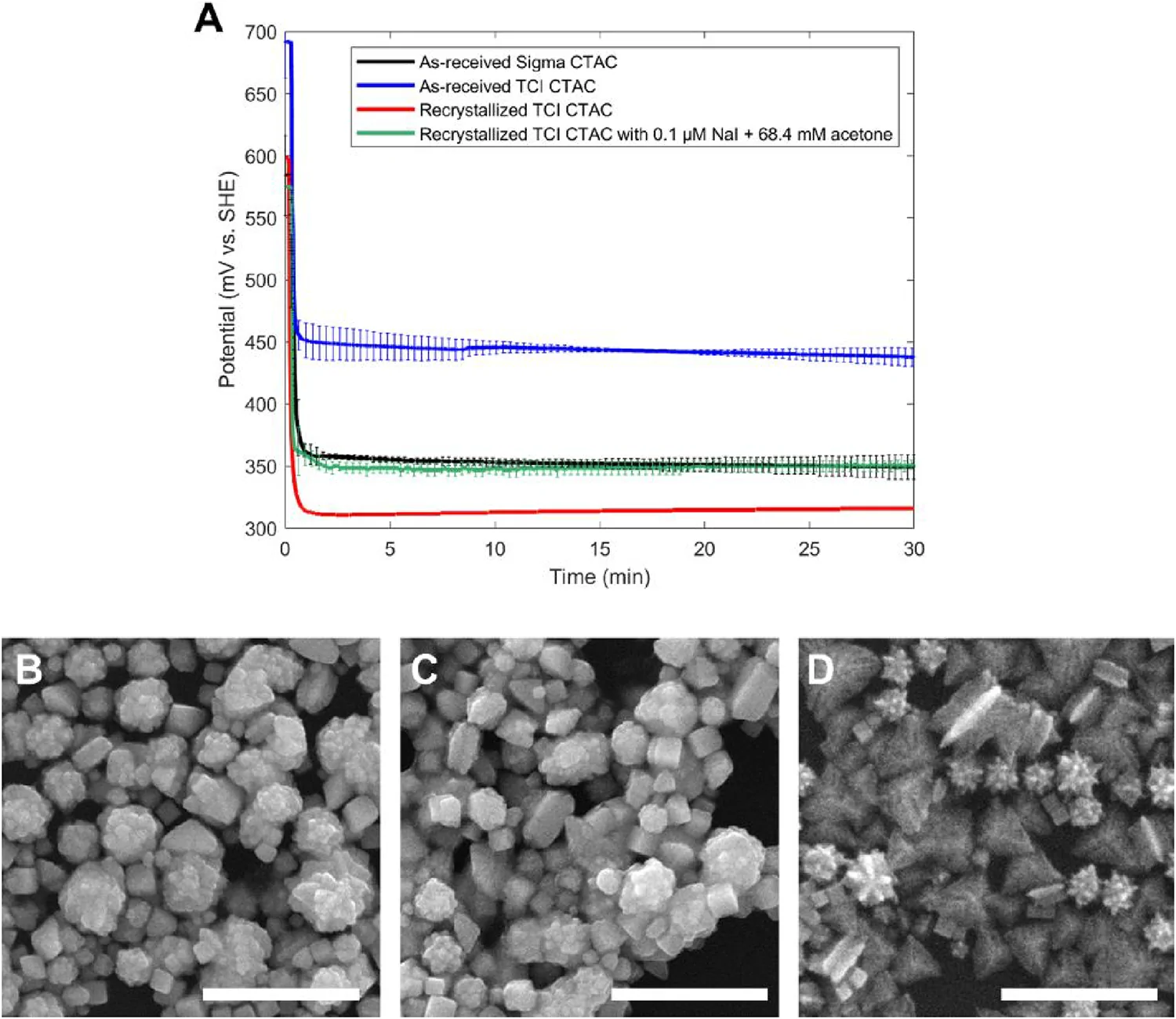
(A) Comparison between SJ epoxy ORP measurements (Pt band WE vs Ag/AgCl RE) of Pd corrugated particles in synthesized in as-received Sigma CTAC (black), as-received TCI CTAC (blue), recrystallized TCI CTAC (red), and recrystallized, oven-dried TCI CTAC with 0.10 μM NaI and 50 μL neat acetone additives (equal to 68.4 mM acetone in the growth solution; green). Error bars represent the standard deviation of three measurements. (B–D) SEM images of Pd corrugated particles synthesized in (B) as-received TCI CTAC, (C) recrystallized TCI CTAC, and (D) recrystallized, oven-dried TCI CTAC with 0.10 μM NaI and 50 μL neat acetone (equal to 68.4 mM acetone in the growth solution). Scale bars: 500 nm.

To investigate adventitious small, polar organics as a secondary handle for shape direction, gradients of low concentrations of acetone and iodide were added to Pd corrugated particle growth solutions in recrystallized TCI CTAC and the products were characterized by SEM (Figure S32). Acetone was chosen due to its prior use as a shape-directing additive in our synthesis of Pd THH in CTAB.^24^ Generally, as concentrations of both iodide and acetone additives were increased, the morphology of Pd particles grew less rounded and they decreased in size. Of the additive combinations tested, a growth solution with 0.10 μM NaI and 50 μL neat acetone (68.4 mM acetone in the growth solution) in recrystallized TCI CTAC produced the closest match of particle morphology to typical results in Sigma CTAC, in terms of particle size and the sharpness of corrugated surfaces (Figures 9D, S32C, and S33). SJ epoxy ORP sensor measurement of Pd corrugated particle growth in this condition corresponded well with the as-received Sigma CTAC benchmark (±5 mV difference in potentials), confirming that this modification condition produces both the correct Pd^2+^ reduction kinetics (and thus OCP) and the correct surface effects (Figure 9A).

These optimized additive concentrations for recrystallized TCI CTAC (0.10 μM NaI and 68.4 mM acetone) were then tested for (Ag)Au growth. Products were well-formed octahedra, rhombic dodecahedra and {110}-faceted bipyramids, truncated ditetragonal prisms and shard-like particles, and concave cube particles at the expected Ag^+^ concentrations (Figure 10). DJ glass ORP measurements of recrystallized TCI CTAC growth solutions with iodide and acetone modification for all four Ag^+^ conditions corresponded well with ORP sensor and potentiostat OCP benchmark measurements of the (Ag)Au particle series in as-received, unmodified Sigma CTAC.

**Figure 10:**
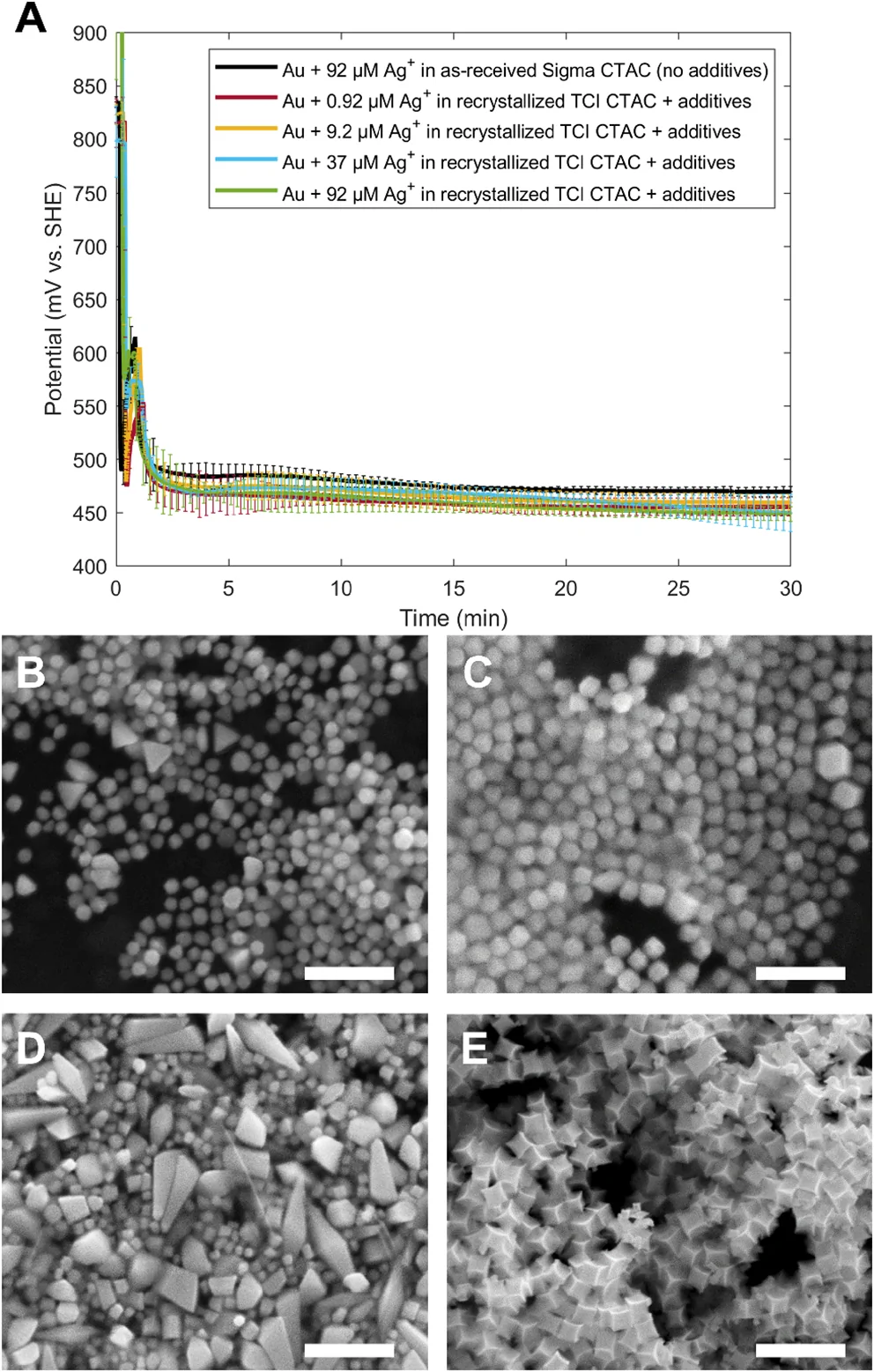
(A)­DJ glass ORP sensor measurements (Pt band WE vs Ag/AgCl RE) of Au particle synthesis in recrystallized, oven-dried TCI CTAC, with 0.10 μM NaI and 68.4 mM acetone additives in the growth solution to control growth kinetics and different concentrations of Ag^+^ added for surface passivation-based shape direction (red, yellow, blue, green); as well as the benchmark DJ glass ORP measurement of Au concave cube synthesis with 92 μM AgNO_3_ in as-received Sigma CTAC (black). Error bars represent the standard deviation of three measurements. (B–E) SEM images of Au nanoparticle shape in recrystallized, oven-dried TCI CTAC with NaI and acetone additives as a function of Ag^+^ concentration: (B) 0.92 μM (octahedra, red trace), (C) 9.2 μM (rhombic dodecahedra, yellow trace), (D) 37 μM (truncated ditetragonal prisms and bipyramids, blue trace), and (E) 92 μM AgNO_3_ (concave cubes, green trace) in the growth solution. Scale bars: 500 nm.

Additional confirmation of the role of organic solvent additives in the (Ag)Au synthesis was provided by lyophilizing and drying the as-received aqueous Sigma CTAC solution to obtain a powder that was free of solvent contaminants. The lyophilized Sigma CTAC solids were divided; half were used without further purification and half were recrystallized and oven-dried again. The lyophilized Sigma CTAC and lyophilized, recrystallized Sigma CTAC were characterized via ^1^H NMR (Figures S34–S35) and both were found to be free of impurities. Au particles synthesized with 92 μM Ag^+^ in lyophilized Sigma CTAC (not recrystallized) were a mixture of large concave cubes, truncated ditetragonal prisms, and shard-like particles, indicative of a slightly low Ag surface coverage, and a DJ glass ORP sensor measurement of these conditions was shifted about 80 mV less positive in potential than the benchmark OCP in as-received Sigma CTAC (Figure S36). The lyophilized Sigma CTAC is assumed to have retained the same iodide concentration as as-received Sigma CTAC, therefore the decrease in solution potential due to drying of the CTAC is attributable to the removal of a solvent impurity—likely the impurity noted in the ^1^H NMR of as-received Sigma CTAC (Figure S29). Indeed, addition of 50 μL of neat acetone (68.4 mM) to a Au nanoparticle growth solution with 92 μM Ag^+^ in lyophilized Sigma CTAC (not recrystallized) led to an ORP trace that matched the as-received Sigma CTAC benchmark for (Ag)­Au concave cubes within ±5 mV, as well as producing high-quality concave cubes (Figure S37).

Further confirming the synergistic role of beneficial iodide and solvent impurities in CTAC, Au growth solutions prepared in lyophilized, recrystallized Sigma CTAC and modified with 0.10 μM NaI and 50 μL of neat acetone (68.4 mM) produced the expected rounded octahedra, rhombic dodecahedra, rounded truncated ditetragonal prisms and shard-like particles, and concave cubes with 0.92 μM, 9.2 μM, 37 μM, and 92 μM Ag^+^, respectively (Figure 11B–E). DJ glass ORP sensor measurements of the four growth solutions were consistent with one another (±10 mV) and with the as-received Sigma CTAC benchmark measurements (±10 mV; Figure 11A), further confirming that these modifications create both the correct kinetic minimum for Au ion reduction and conditions for optimal Ag UPD shape direction.

**Figure 11:**
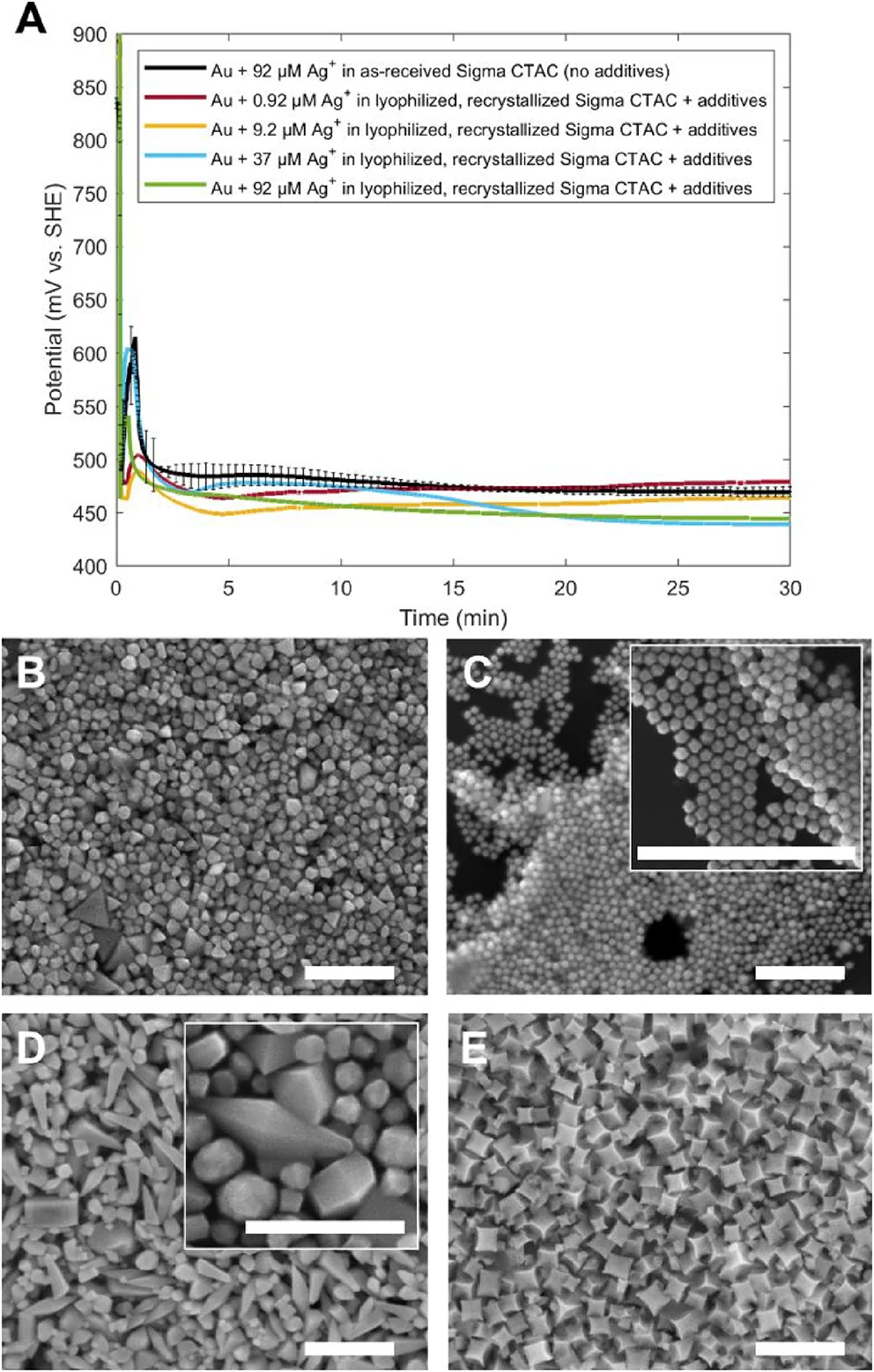
(A) DJ glass ORP sensor measurements (Pt band WE vs Ag/AgCl RE) of Au particle synthesis in lyophilized, recrystallized Sigma CTAC, with 0.10 μM NaI and 68.4 mM acetone additives in the growth solution to control growth kinetics and different concentrations of Ag^+^ added for surface passivation-based shape direction (red, yellow, blue, green), and DJ glass ORP benchmark measurement of the Au concave cube synthesis with 92 μM AgNO_3_ in as-received Sigma CTAC (black). Error bars represent the standard deviation of three measurements. (B–E) SEM images of Au nanoparticle shape in lyophilized, recrystallized Sigma CTAC with NaI and acetone additives as a function of Ag^+^ concentration: (B) 0.92 μM (octahedra, red trace), (C) 9.2 μM (rhombic dodecahedra, yellow trace), (D) 37 μM (truncated ditetragonal prisms and bipyramids, blue trace), and (E) 92 μM AgNO_3_ (concave cubes, green trace) in the growth solution. Scale bars: 500 nm.

As alternatives to acetone, addition of equimolar (∼68.4 mM) amounts of ethanol (EtOH), methanol (MeOH), isopropanol (IPA), or chloroform to growth solutions prepared in recrystallized, oven-dried TCI CTAC with 0.10 μM NaI and 92 μM Ag^+^ also led to the benchmark mixed potential (+470 ± 10 mV) when measured with a DJ glass ORP sensor. The products were monodisperse concave cube particles with addition of EtOH or MeOH and concave cube particles with small, poorly formed side products with addition of IPA or chloroform (Figure S38). This indicates that the mechanism by which small organic contaminants in CTAC affect (Ag)Au particle growth kinetics is not specific to acetone or any other solvent tested, supporting a mechanism of intercalation of the solvent molecules between CTA^+^ molecules in the surfactant bilayer,^24^ rather than reactivity of the organic contaminants with any other reagent and/or specific binding to the particle surface. Given the ubiquity of 25 wt % Sigma CTAC as a reagent in literature syntheses of metal nanoparticles, adventitious organic solvent impurities may represent a critical, previously unidentified contributor to a variety of mechanisms of metal nanoparticle growth, and the kinetic effects of such solvent impurities can be readily captured by in situ ORP sensor measurements these syntheses.

## 3 Conclusions

Overall, our studies of Pd, Au, Ag­(Au), and Cu model systems indicate that ORP sensors present an accurate, viable alternative to potentiostat measurements of the OCP of metal nanoparticle growth in many situations, and, importantly, can be used for benchmarking and troubleshooting analogously to potentiostat OCP measurements, with a significant decrease in the cost of use. The commercial availability and low cost of ORP sensors—even as compared to the already low cost of a potentiostat when considered alongside other instruments for time-resolved characterization of metal nanoparticle growth—opens the possibility of their routine use for synthetic benchmarking and troubleshooting. The development of a commercial ORP sensor with a Ag-free RE, such as the Hg-based REs already commonly used in electrochemical applications (e.g., Hg/Hg_2_SO_4_; saturated calomel, SCE), and/or with a more inert WE than Pt or other noble metals (e.g., glassy carbon or graphite), would further expand the range of growth chemistries whose OCP can be benchmarked by use of ORP sensors. Likewise, an ORP sensor with a polishable WE to enable facile removal of oxide layers would bypass present limitations on the use of these sensors for detailed mechanistic studies in the subset of nanoparticle syntheses where early changes in reaction chemistry are critical to the growth trajectory.

Due to the demand for higher-throughput methods for time-resolved metal nanoparticle characterization, as well as the need to better describe synthetic reproducibility issues, ORP sensors could be employed for facile “benchmark” OCP measurements of many syntheses, without requiring an individual potentiostat to make each measurement. Generation of a data set of such measurements could be combined with the increasing number of tools incorporating artificial intelligence or machine learning approaches to extract insights about relationships between solution chemistry and time-resolved solution potential, with these relationships then informing future synthetic design. The low cost and ease of use of ORP sensors could also better allow for the use of OCP measurements of nanoparticle synthesis in a teaching setting, or for researchers to check their work easily without use of electron microscopy or other advanced and/or expensive characterization methods.

## 4 Experimental Section

### 4.1 Reagents

Cetyltrimethylammonium bromide (CTAB, BioXtra Lot No. SLCJ8356, ≥99%), cetyltrimethylammonium chloride solution (CTAC, Lot No. STBL3571, 25 wt % solution in water), hexadecylamine (HDA, 98%), l-ascorbic acid (AA, ACS reagent grade, ≥99%), potassium iodide (KI, ≥99.99% trace metals basis), sodium borohydride (NaBH_4_, 99.99% trace metal basis), sodium tetrachloropalladate­(II) (Na_2_PdCl_4_, ≥99.99% trace metal basis), gold­(III) chloride trihydrate (chloroauric acid, HAuCl_4_·3H_2_O, ≥99.9% trace metals basis), and silver nitrate (AgNO_3_, 99.9999% trace metal basis) were purchased from MilliporeSigma. Hydrochloric acid (HCl, 1 M solution) was purchased from VWR. Nitric acid solution (HNO_3_, 1 M solution), copper­(II) chloride dihydrate (CuCl_2_·2 H_2_O, ACS reagent grade, ≥99%), l-ascorbic acid sodium salt (NaAsc, 99%), acetone (ACS grade, ≥99.5%), methanol (ACS grade, ≥99.8%), isopropanol (ACS grade, ≥99.8%), chloroform (ACS reagent grade, ≥99.8%, stabilized with ethanol), sodium iodide (NaI, molecular biology grade, ≥99%), and palladium­(II) chloride (PdCl_2_, 99.9% metals basis) were purchased from Thermo Fisher Scientific. Copper­(II) acetate monohydrate (Cu­(OAc)_2_·H_2_O, ≥98%) was purchased from Alfa Aesar. Cetyltrimethylammonium chloride powder (CTAC, Lot No. CN6VE-OK, >95.0%) was purchased from Tokyo Chemical Industries. Ethanol (190 proof) was purchased from Decon Laboratories. An extended list of reagents is available in the Supporting Information. All chemicals were used without further purification (with the exception of oven drying of CTAB and CTAC, recrystallization of CTAC, and lyophilization of CTAC, as described in the Supporting Information) and all solutions were prepared with ultrapure deionized (DI) water (18.2 MΩ resistivity, Milli-Q IQ 7000).

### 4.2 Nanoparticle Synthesis

#### 4.2.1 Corrugated Pd Nanoparticles

Corrugated Pd nanoparticles were synthesized using a previously reported method.^55^ A growth solution was prepared by adding CTAC (Sigma CTAC or TCI CTAC or recrystallized TCI CTAC, 10 mL, 12.5 mM), Na_2_PdCl_4_ (500 μL, 10 mM), and HNO_3_ (30 μL, 1 M) to a 20 mL scintillation vial, which was then swirled. AA (100 μL, 100 mM) was then added to the growth solution, followed by a 100 μL aliquot of 10,000× diluted small pseudospherical Pd seeds. The growth solution was swirled vigorously and left undisturbed for ≥30 min.

#### 4.2.2 Pd THH Nanoparticles

Pd THH nanoparticles were synthesized according to a previously reported method.^24,44^ A growth solution was prepared by adding CTAB (oven-dried BioXtra, 10 mL, 50 mM), Na_2_PdCl_4_ (200 μL, 10 mM), HNO_3_ (100 μL, 100 mM), NaI (60 μL, 0.1 mM), and neat acetone (230 μL) to a 20 mL scintillation vial in a 40 °C water bath. The solution was allowed to equilibrate in the heated bath for at least 10 min before addition of a 100 μL aliquot of 10× diluted Pd nanocube seeds. The solution was swirled vigorously. AA (100 μL, 100 mM) was then added, and the solution was swirled again. The reaction was allowed to proceed at 40 °C for at least 15 h.

#### 4.2.3 Monometallic Au Nanoparticles

Au nanoparticles were synthesized via modification of a previously reported method.^44,61,62^ A growth solution was prepared by adding Sigma CTAC (10 mL, 100 mM), HAuCl_4_ (500 μL, 10 mM), and HCl (200 μL, 1 M) to a 20 mL scintillation vial. AA (100 μL, 100 mM) was added, and the vial was swirled again. An aliquot of 100 μL 1000× diluted Au seed particles was added to the growth solution, and the vial was swirled vigorously and left undisturbed at room temperature for at least 30 min.

#### 4.2.4 (Ag)Au Nanoparticles

(Ag)Au nanoparticles were synthesized using a previously reported method.^61,62^ A growth solution was prepared by adding CTAC (Sigma CTAC or TCI CTAC or recrystallized TCI CTAC or lyophilized, recrystallized Sigma CTAC, 10 mL, 100 mM), HAuCl_4_ (500 μL, 10 mM), HCl (200 μL, 1 M, and *x* μL) of freshly prepared AgNO_3_ (*x* = 1 μL for octahedra, 10 μL for rhombic dodecahedra, 40 μL for truncated ditetragonal prisms, and 100 μL for concave cubes, 10 mM) to a 20 mL scintillation vial and swirling vigorously. AA (100 μL, 100 mM), was then added and the vial was swirled vigorously before a 100 μL aliquot of 1000× diluted Au seed particles was added. The reaction was swirled immediately after the seed addition and then left undisturbed at room temperature for ∼3 h.

#### 4.2.5 Homogeneously Nucleated Cu Nanocubes

Homogeneously nucleated Cu cubic nanoparticles were synthesized via modification of a previously reported method.^75^ In a 20 mL scintillation vial, Sigma CTAC (9.775 mL, 15 mM) and Cu­(OAc)_2_ (75 μL, 100 mM) were added and the solution was stirred at 750 rpm at room temperature. NaAsc (170 μL, 500 mM) was added, and the mixture was stirred for 1 min at room temperature before being heated to 90 °C under minimal stirring for 40 min.

#### 4.2.6 Pd-Seeded Cu Nanocubes

Pd-seeded Cu nanocubes were synthesized via modification of a previously reported method.^87^ HDA (60 mg), ultrapure DI water (9.1 mL), and CuCl_2_ (500 μL, 100 mM) were added in a scintillation vial, sonicated, and vigorously stirred overnight. While stirring at 600 rpm, a 120 μL aliquot of undiluted Pd nanocube seeds was added to the mixture and stirred for 5 min before AA (200 μL, 1 M) was added. The mixture was immediately heated to 100 °C for 1 h under continuous stirring.

Refer to the Supporting Information for a more detailed description of nanoparticle synthesis procedures, description of the syntheses of the seeds used in each reaction, and detailed procedures for the recrystallization, drying, and lyophilization of CTAC.

### 4.3 Electrochemical Measurements

#### 4.3.1 Potentiostat OCP Measurements

Potentiostat OCP measurements of the nanoparticle syntheses were performed in a five-neck electrochemistry flask (Gamry Dr. Bob’s Cell; Pd, Au, Ag­(Au)) or directly in the scintillation vial (Cu). The measurements used one of: a Ag/AgCl RE (Koslow Scientific or Pine Research or eDAQ), a Hg/Hg_2_SO_4_ RE (Koslow Scientific), or a RHE RE (Gaskatel, Hydroflex), depending on the synthesis measured. The RE was either inserted in a bridge tube containing 100 mM H_2_SO_4_ or directly inserted into the growth solution. The WE was a freshly polished disk insert GCE (Pine Research), freshly polished fixed disk GCE (Pine Research), Pt wire (Gamry), Pt gauze (Thermo Scientific), or a freshly polished or “oxidized” fixed disk Pt WE (Gamry). No CE was used. Refer to the Supporting Information for details on electrode storage and preparation and a detailed description of the WE and RE electrode pair used for each specific reaction condition.

For a standard OCP measurement, the nanoparticle growth solution was prepared in the same way as in the unmeasured colloidal synthesis, adding all chemicals except for the seeds and reducing agent. Before the addition of seeds and/or reducing agent, the electrodes were placed into the growth solution and the leads connected to the potentiostat (Gamry Instruments 1010E). After waiting for ∼5 min, the OCP measurement was started. Growth was initiated via the injection of the seeds and/or reducing agent, analogously to unmeasured colloidal synthesis. After the measurements were completed, electrodes were removed, rinsed with ultrapure DI water, and cleaned.

#### 4.3.2 ORP Sensor Measurements

ORP sensor measurements were collected using Vernier Graphical Analysis software. The ORP measurements were carried out analogously to the potentiostat measurements; however, instead of an RE and a WE, the ORP sensor (Cole-Parmer, ORP electrode, double-junction, platinum band, 10-ft cable, EE-27006–21 (“DJ glass ORP”) or Vernier Science Education, ORP Sensor, ORP-BTA (“SJ Epoxy ORP”)) was inserted into the growth solution. All ORP measurements were conducted in 20 mL scintillation vials. Prior to use, the ORP sensor was calibrated (see Supporting Information for details).

For a standard ORP measurement, the nanoparticle growth solution was prepared in the same way as in the unmeasured colloidal synthesis with all chemicals except for seeds and reducing agent. Before the addition of seeds and or reducing agent, the ORP sensor was placed into the growth solution and, after waiting for ∼5 min, the ORP measurement was started. Growth was initiated via the injection of the seeds and/or reducing agent, analogously to unmeasured colloidal synthesis. After the measurements were completed, electrodes were removed, rinsed with ultrapure DI water, and cleaned.

For detailed information on the duration of the OCP/ORP measurements, electrode/ORP sensor cleaning procedures, and RE potential transformation to the SHE convention, refer to the Supporting Information.

### 4.4 Characterization Methods

Nanoparticle morphology was characterized using a FEI Quanta 650 field emission SEM. SEM samples were prepared by washing the nanoparticles with either ultrapure DI water or EtOH and dropcasting the samples onto a silicon wafer, which was then allowed to dry. NMR spectra were recorded on a Bruker Avance III 600 spectrometer (600 MHz, ^1^H). Samples were dissolved in either a 9:1 mixture of ultrapure DI water and D_2_O or pure D_2_O. NMR data were processed using MestReNova v14.3.3–33362. Inductively coupled plasma optical emission spectroscopy (ICP-OES) measurements of digested nanoparticle growth solutions were carried out in triplicate using a PerkinElmer Avio 200 ICP-OES spectrometer. For detailed sample preparation procedures and further details on the characterization methods used, refer to the Supporting Information.

## Supplementary Material



## Notes

Detailed experimental materials and methods, discussion of guidelines for applicability and interpretation of ORP measurements, NMR data, additional SEM images, quantification of particle size and morphology distributions, additional graphs of OCP profiles, photographs of electrode materials and ORP sensors, and NMR spectra of surfactant solutions (PDF)
