# Supplementary material for: Accessible Chemical Benchmarking of Nanoparticle Growth Using Oxidation–Reduction Potential Sensors
Source: Chem Mater. 2026 Aug 27;38(17):8968–86. doi: 10.1021/acs.chemmater.6c01559 (PMC13560467; doi:10.1021/acs.chemmater.6c01559)
Supplement: Supplementary file 1 [file cm6c01559_si_001.pdf]

Supporting Information for

Accessible Chemical Benchmarking of Nanoparticle Growth using Oxidation-Reduction Potential Sensors

Gabriel C. Halford,[a,†] Sebastian Hertle,[a,†] Katrina L. Obedin,[a] Abigail M. Sublett,[a] Kaelyn M. Harmon,[b] Lauren E. VanGelder,[c] Michelle L. Personick*[a]

[a] Department of Chemistry, University of Virginia, Charlottesville, Virginia 22904, United States
[b] Department of Materials Science and Engineering, University of Virginia, Charlottesville, Virginia 22904, United States
[c] Department of Chemistry, Norfolk State University, Norfolk, Virginia 23504, United States

* Email: mpersonick@virginia.edu
[†]G.C.H. and S.H. contributed equally to this work.

Replication Data

Halford, Gabriel C.; Hertle, Sebastian; Obedin, Katrina L.; Sublett, Abigail M.; Harmon, Kaelyn M.; VanGelder, Lauren E.; Personick, Michelle L., 2026, "Replication Data for: "Accessible Chemical Benchmarking of Nanoparticle Growth using Oxidation-Reduction Potential Sensors," https://doi.org/10.18130/V3/5NDGAM, University of Virginia Dataverse.

Table of Contents:

I. Experimental Methods S5
1. Materials S5
2. Synthesis Procedures S8
   - 2.1. Oven-Drying Procedure for CTAB and CTAC Powders S8
   - 2.2. Recrystallization of CTAC Powder S8
   - 2.3. Lyophilization and Vacuum Oven Drying of Sigma CTAC S9
   - 2.4. Synthesis of Small Pseudospherical Pd Seeds S9
   - 2.5. Synthesis of Pd Nanocube Seeds for Pd THH Growth S10
   - 2.6. Synthesis of Pd Nanocube Seeds for HDA/AA Cu Nanocube Growth S10
   - 2.7. Synthesis of Au Seeds S11
   - 2.8. Colloidal Synthesis of Corrugated Pd Nanoparticles in Sigma CTAC S11
   - 2.9. Colloidal Synthesis of Pd THH Nanoparticles in Dried BioXtra CTAB S12

2.10. Colloidal Synthesis of Monometallic Au Nanoparticles S12

2.11. Colloidal Synthesis of Shape-Controlled (Ag)Au Nanoparticles in As-Received Sigma CTAC S13

2.12. CTAC/NaAsc Synthesis of Homogeneously Nucleated Cu Nanocubes S13

2.13. HDA/AA Synthesis of Pd-Seeded Cu Nanocubes S14

2.14. Colloidal Synthesis of Shape-Controlled (Ag)Au Nanoparticles in As-Received TCI CTAC S14

2.15. Colloidal Synthesis of Shape-Controlled (Ag)Au Nanoparticles in Recrystallized TCI CTAC S14

2.16. Colloidal Synthesis of Shape-Controlled (Ag)Au Nanoparticles in Lyophilized, Recrystallized Sigma CTAC S15

3. Electroanalytical Methods S16

3.1. Electrode Preparation and Reference Electrode Benchmarking for Potentiostat OCP S16

3.2. Working Electrode Surface Area Calculations S20

3.3. Cleaning of Electrochemistry Glassware S21

3.4. Calibration and Cleaning of ORP Sensors S22

3.5. Potentiostat Open-Circuit Potential Measurements of Colloidal Pd Corrugated Particle Synthesis S23

3.6. ORP Sensor Open-Circuit Potential Measurements of Colloidal Pd Corrugated Particle Synthesis S24

3.7. Potentiostat Open-Circuit Potential Measurements of Colloidal Pd THH Synthesis S25

3.8. ORP Sensor Open-Circuit Potential Measurements of Colloidal Pd THH Synthesis S25

3.9. Potentiostat Open-Circuit Potential Measurements of Colloidal Au and (Ag)Au Particle Synthesis S26

3.10. ORP Sensor Open-Circuit Potential Measurements of Colloidal Au and (Ag)Au Particle Synthesis S27

3.11. Potentiostat Open-Circuit Potential Measurements of CTAC/NaAsc Homogeneously Nucleated Cu Nanocubes S28

3.12. ORP Sensor Open-Circuit Potential Measurements of CTAC/NaAsc Homogeneously Nucleated Cu Nanocubes S29

3.13. Potentiostat Open-Circuit Potential Measurements of HDA/AA Pd-Seeded Cu Nanocubes S30

3.14. ORP Sensor Open-Circuit Potential Measurements of HDA/AA Pd-Seeded Cu Nanocubes S31
4. Other Methods S32
4.1. Electron Microscopy Characterization S32
4.2. ^{1}H-Nuclear Magnetic Resonance (NMR) Characterization of CTAC S32
4.3. ICP-OES Sample Preparation and Analysis S33
II. Best Practices for ORP Sensor Measurements of Metal Nanoparticle Synthesis S34
1. Considerations for Choosing an ORP Sensor Type S34
1.1. Possibility for Introduction of Contaminants During ORP Measurement S34
1.2. Working Electrode Material and Area S34
1.3. Working Temperature Range of the ORP Sensor S35
2. Considerations for Interpretation of ORP Sensor Measurements S35
2.1. Influence of the ORP Sensor on the Reaction Temperature S35
2.2. ORP Sensor Sensitivity and Measurement of Diffusion Effects S36
III. NMR Data for Characterization of CTAC S37
IV. Supplementary Figures and Tables S38
Table S1 .... S38
Figure S1. .... S38
Figure S2. .... S39
Table S2. .... S39
Figure S3. .... S40
Figure S4. .... S40
Table S3. .... S40
Figure S5. .... S41
Figure S6. .... S42
Figure S7. .... S43
Figure S8. .... S44
Figure S9. .... S44
Figure S10. .... S45
Figure S11. .... S46
Figure S12. .... S47
Figure S13. .... S48

Figure S14. .... S49

Figure S15. .... S50

Figure S16. .... S50

Figure S17. .... S51

Figure S18. .... S52

Figure S19. .... S52

Figure S20. .... S53

Figure S21. .... S54

Figure S22. .... S55

Figure S23. .... S55

Figure S24. .... S56

Figure S25. .... S57

Figure S26. .... S58

Figure S27. .... S59

Figure S28. .... S60

Figure S29. .... S61

Figure S30. .... S62

Figure S31 .... S62

Figure S32. .... S63

Figure S33. .... S64

Figure S34. .... S64

Figure S35. .... S65

Figure S36. .... S66

Figure S37. .... S67

Figure S38. .... S68

References. S69

I. Experimental Methods

1. Materials

Cetyltrimethylammonium bromide (CTAB, BioXtra Lot No. SLCJ8356, ≥99%), cetyltrimethylammonium chloride solution (CTAC, Lot No. STBL3571, 25 wt. % solution in water), ethyl acetate (HPLC-grade, ≥99.7%), hexadecylamine (HDA, 98%), L-ascorbic acid (AA, ACS reagent grade, ≥99%), potassium iodide (KI, ≥99.99% trace metals basis), sodium borohydride ($NaBH_4$, 99.99% trace metal basis), sodium tetrachloropalladate(II) (Na_2PdCl_4, ≥99.99% trace metal basis), gold(III) chloride trihydrate (chloroauric acid, $HAuCl_4 \cdot 3H_2O$, ≥99.9% trace metals basis), and silver nitrate ($AgNO_3$, 99.9999% trace metal basis) were purchased from MilliporeSigma. Hydrochloric acid (HCl, 1M solution) was purchased from VWR. Nitric acid solution (HNO_3, 1M solution), concentrated hydrochloric acid (HCl, ACS Plus grade), concentrated nitric acid (HNO_3, ACS Plus grade), copper (II) chloride dihydrate ($CuCl_2 \cdot 2H_2O$, ACS reagent grade, ≥99%), L-ascorbic acid sodium salt (NaAsc, 99%), sulfuric acid solution (H_2SO_4, 0.5 M solution), acetone (ACS grade, ≥99.5%), methanol (ACS grade, ≥99.8%), isopropanol (ACS grade, ≥99.8%), chloroform (ACS reagent grade, ≥99.8%, stabilized with ethanol), acetone (HPLC grade, 99.8%), sodium iodide (NaI, molecular biology grade, ≥99%), palladium (II) chloride ($PdCl_2$, 99.9% metals basis), and saturated potassium chloride electrode storage solution (KCl, solution in water with potassium hydrogen phthalate stabilizer) were purchased from Thermo Fisher Scientific. Copper (II) acetate monohydrate ($Cu(OAc)_2 \cdot H_2O$, ≥98%) was purchased from Alfa Aesar. Cetyltrimethylammonium chloride powder (CTAC, Lot No. CN6VE-OK, >95.0%) was purchased from Tokyo Chemical Industries. Alumina powder (0.05 μm and 0.3 μm MicroPolish) was purchased from Buehler. Ethanol (190 proof) was purchased from Decon Laboratories. Saturated potassium sulfate electrode storage solution

(K_2SO_4, in water) was purchased from Koslow Scientific Co. 200 mV ORP test solution (potassium ferricyanide/$K_3[Fe(CN)_6]$, in water) and 600 mV ORP test solution (mixture of iron(III) ammonium sulfate/$(NH_4)Fe(SO_4)_2$ and iron(II) sulfate/$FeSO_4$, in water) were purchased from GLI International/Hach. Silver (Ag) ICP standard solution (999 µg/mL; 5% (v/v) HNO_3) was purchased from Inorganic Ventures.

Sodium tetrachloropalladate(II) (Na_2PdCl_4, 50 mM solution) was prepared by combining 0.294 g Na_2PdCl_4 and 20 mL of ultrapure deionized (DI) water (18.2 MΩ resistivity, MilliQ IQ 7000). The solution was capped and stirred for 3 hours until fully dissolved. The reported Pd THH synthesis is highly sensitive to the concentration of Pd^{2+} in the growth solution; for that reason, all synthesis reactions for this material were conducted by diluting a more stable 50 mM stock solution of Na_2PdCl_4 to 10 mM. This 10 mM solution was then used the same day. When a new stock solution (50 mM) was created, a tight gradient (0.200-0.300 mL of 10 mM Na_2PdCl_4 in the growth solutions) was conducted to establish a consistent, high-quality THH product due to apparent solubility issues between prepared Pd stock solutions.[1]

Tetrachloropalladic acid (H_2PdCl_4, 100 mM solution) was prepared by combining 0.354 g $PdCl_2$ and 20 mL of 0.2 M HCl in a 30 mL scintillation vial. The solution was capped and stirred for 3 hours until the solid fully dissolved and the solution became a clear dark orange. This solution did not suffer from the solubility issues that the sodium salt did, so calibration was not required.

All chemicals were used without further purification (with the exception of oven drying of CTAB and CTAC, recrystallization of CTAC, and lyophilization of CTAC, as described below) and all solutions were prepared with ultrapure DI water. All sodium iodide and potassium iodide solutions were prepared fresh daily. All ascorbic acid, sodium borohydride, and silver nitrate solutions, as well as diluted seed solutions, were prepared immediately before use and re-made

fresh on the half-hour as needed. Organic solvent additives were not added to surfactant solutions until ~10 minutes before synthesis/measurement and solutions were kept capped, to avoid evaporation.

The syringe filters used to prepare samples for inductively coupled plasma spectroscopy were GE Healthcare Life Sciences Whatman GD/X 25 mm syringe filters, 0.2 µm pore, PTFE membrane. Glass vials used for synthesis were (1) DWK Life Sciences 986560; Wheaton™ glass 20 mL scintillation vials: polypropylene caps, foamed polyethylene liner ("20 mL scintillation vial") and (2) Fisherbrand™ Class A clear glass threaded vials with attached caps, PE Poly-Seal™ Cone Liner ("30 mL scintillation vial"), as specified for individual synthesis procedures. The glass flasks used for potentiostat OCP measurements of Pd and Au nanoparticle synthesis were Gamry Dr. Bob's Cells. The following working electrode (WE) materials were used: (1) glassy carbon disk insert electrode (GCE), 5 mm OD x 4 mm thick, Pine Research; (2) fixed disk glassy carbon electrode (GCE), 6.35 mm OD, Pine Research; (3) Pt wire, 0.406 mm diameter x 15 cm (6 in) length, Gamry (sold as 'counter electrode'); (4) Pt gauze, 100 mesh woven from 0.0762 mm (0.003 in) diameter wire, 25 x 25 mm, 0.53 g, 99.9% (metals basis), Thermo Scientific #010282.FF; and (5) fixed disk Pt electrode, 3 mm OD, Gamry. The following reference electrodes (RE) were used: (1) large silver/silver chloride (Ag/AgCl), saturated KCl electrolyte, 9 mm borosilicate glass body, Koslow Scientific Co.; (2) low-profile Ag/AgCl, 4 M KCl gel, Pine Research; (3) mercury/mercurous sulfate (Hg/Hg_2SO_4), saturated K_2SO_4 electrolyte, 9 mm borosilicate glass body, Koslow Scientific Co.; and (4) reversible hydrogen electrode (RHE), HydroFlex, Gaskatel. The following ORP sensors were used: (1) Vernier Science Education, ORP Sensor, ORP-BTA ("SJ Epoxy ORP") and (2) Cole-Parmer, ORP electrode, double-junction, platinum band, 10-ft cable, EE-27006-21 ("DJ glass ORP").

2. *Synthesis Procedures*

2.1. Oven-Drying Procedure for CTAB and CTAC Powders

CTAB or CTAC powder was placed into a tall 30 mL scintillation vial. The vial was placed uncapped in an oven at 80 °C for at least 24 hours. CTAB was dried in ~10 g batches. The CTAB was stirred with a new Pasteur pipette after ~12 hours to ensure appropriate drying of powder at the bottom of the vial. Vials of dried CTAB were then stored capped on the benchtop without further purification.

2.2. Recrystallization of CTAC Powder

The procedure for recrystallization of CTAC powder was adapted from a literature procedure[2] and a procedure from the Skrabalak Group at Indiana University.[3] 6.0 to 6.3 g of TCI CTAC powder was dissolved in 6 mL of ACS reagent grade chloroform in a 30 mL scintillation vial. In a 500 mL Erlenmeyer flask, previously cleaned with aqua regia (1:3 ratio of concentrated HNO_3 concentrated HCl; **caution: strong acid**), ~200 mL of HPLC-grade ethyl acetate was combined with ~50 mL ACS reagent grade chloroform and heated to 40 °C while stirring at vortex. The CTAC/chloroform mixture was added slowly with a Pasteur pipette. If turbidity or precipitation was observed as CTAC was added, ~1 mL of chloroform was added repeatedly until the mixture became clear again. Once all of the CTAC/chloroform mixture was added to the Erlenmeyer flask, the flask was removed from heat and covered with a watch glass for 15 minutes at ambient temperature. If crystallization had visibly begun in 15 minutes, the flask was left undisturbed for 30 more minutes at room temperature; if crystallization had not begun, the covered flask was moved to an ice bath or refrigerator for 40 minutes. If the flask was chilled, it was allowed to return to room temperature for 20 minutes before proceeding further. The contents of the flask were transferred to a vacuum filtration apparatus; a small amount (< 2 mL) of HPLC-grade acetone was

used to transfer any stuck crystals from the flask for filtration. A layer of cold HPLC-grade ethyl acetate was poured over the crystals, and they were vacuum filtered for 30 minutes. CTAC crystals were collected in a fresh 30 mL scintillation vial and the procedure was repeated for a total of three recrystallizations. After the third vacuum filtration, CTAC crystals were collected in a 20 mL vial and dried for at least 24 hours in an 80 °C oven. Following oven drying, recrystallized CTAC was stored on the benchtop.

2.3. Lyophilization and Vacuum Oven Drying of Sigma CTAC

40 mL of Sigma CTAC (25 wt. % solution in water) was frozen to -80 °C in a 50 mL polypropylene Falcon tube and dried via lyophilization (~100 hours). The lyophilized sample formed a hydrated gel phase, which was transferred via spatula to a crystallizing dish and subsequently dried again by vacuum oven (100 °C, ~125 torr, ~100 hours) to form a crystalline powder. The lyophilized Sigma CTAC was characterized by ^{1}H-NMR. Half was used without further purification. Half was recrystallized using the procedure previously described for recrystallization of powdered TCI CTAC and then oven-dried (not under vacuum) at 80 °C for 24 hours.

2.4. Synthesis of Small Pseudospherical Pd Seeds

<10 nm Pd seeds were synthesized according to our previously reported method.[4] 10 mL of 12.5 mM BioXtra CTAB and 250 μL of 10 mM Na_2PdCl_4 were added to a 20 mL scintillation vial and stirred to vortex (3 x 10 mm stir bar). Once the solution was thoroughly mixed, 600 μL of freshly prepared 10 mM $NaBH_4$ was rapidly injected under continued vortex stirring, inducing an instantaneous darkening of the solution from beige to dark brown. The seed solution was allowed to stir for an additional minute to ensure uniformity in the dispersion of the reducing agent. The

stirring was then halted and the seed solution was aged on the benchtop for two hours to ensure degradation of excess $NaBH_4$. Seeds were used within 24 hours of synthesis.

2.5. Synthesis of Pd Nanocube Seeds for Pd THH Growth

22 nm nanocube seeds were synthesized according to a previously reported method.[5] 10 mL of 12.5 mM BioXtra CTAB, 500 µL of 10 mM H_2PdCl_4, and a stir bar (3 x 10 mm) were added into a tall 30 mL scintillation vial. The vial was then placed in 95 °C silicone oil bath with 600 RPM stirring and allowed to equilibrate for ten minutes. 80 µL of 100 mM AA was injected, and the solution was stirred in the oil bath for 20 minutes. After that, it was transferred to a 30 °C water bath and the seeds were allowed to age for ≥ 2 hours. They maintained their quality for up to 24 hours after synthesis before developing rounded corners and/or aggregating. 10-fold diluted solutions of seeds in 12.5 mM BioXtra CTAB only maintained quality for about 15 minutes at room temperature before beginning to aggregate, so all seed dilutions were prepared immediately before use.

2.6. Synthesis of Pd Nanocube Seeds for HDA/AA Cu Nanocube Growth

~21 nm nanocube seeds were synthesized via adaptation of a previously reported method.[6] In a 30 mL scintillation vial, 100 µL of 100 mM H_2PdCl_4, a stir bar (3 x 10 mm) and 10 mL of 12.5 mM BioXtra CTAB were added and placed in a 90 °C silicone oil bath. While stirring at 600 rpm the solution was allowed to equilibrate for 10 min. Then 50 µL of 10 mM KI was added and the mixture was stirred for an additional 10 min. 80 µL of 100 mM AA was added and the reaction was stirred at 600 rpm for 1 hour at 90 °C. The seeds were stored in a 40 °C water bath and used without further purification or dilution. The seeds were used up to 24 hours after synthesis.

2.7. Synthesis of Au Seeds

~7 nm Au seeds were synthesized according to a previously reported method.[7] 10 mL of 100 mM as-received Sigma CTAC and 250 μL of 10 mM $HAuCl_4$ were added to a 20 mL scintillation vial and stirred to vortex (3 x 10 mm stir bar). Once the solution was thoroughly mixed, 600 μL of freshly prepared 10 mM $NaBH_4$ was rapidly injected under continued vortex stirring, inducing an instantaneous color change of the solution from yellow to red. The seed solution was allowed to stir for an additional minute to ensure uniformity in the dispersion of the reducing agent. The stirring was then halted, and the seed solution was aged on the benchtop for two hours to ensure degradation of excess $NaBH_4$. Seeds were used within 24 hours of synthesis. Au seeds were prepared in as-received Sigma CTAC (25 wt% solution) for all reaction conditions, and then diluted in the same CTAC source used for the growth solution.

2.8. Colloidal Synthesis of Corrugated Pd Nanoparticles in Sigma CTAC

Corrugated Pd nanoparticles were synthesized using our previously reported method.[4] A growth solution was prepared by sequentially adding 10 mL of 12.5 mM Sigma CTAC, 500 μL of 10 mM Na_2PdCl_4, and 30 μL of 1 M HNO_3 to a 20 mL scintillation vial. 100 μL of 100 mM AA was then added to the growth solution. A 100 μL aliquot of solution of small pseudospherical Pd seeds, diluted 10,000x in 12.5 mM BioXtra CTAB, was injected last. The growth solution was swirled vigorously again and left undisturbed for ≥ 30 minutes. This procedure was followed for syntheses with OCP measurements, with the following two exceptions: (1) for both potentiostat OCP and ORP sensor measurements, swirling of the growth solution was replaced by 200 RPM magnetic stirring and (2) potentiostat OCP measurements only were conducted in a five-neck flask (Gamry Dr. Bob's Cell), rather than a 20 mL vial.

2.9. Colloidal Synthesis of Pd THH Nanoparticles in Dried BioXtra CTAB

Pd THH nanoparticles were synthesized according to our previously-reported method.[1,8] A growth solution was prepared by adding 10 mL of 50 mM CTAB (oven-dried BioXtra), 200 µL of 10 mM Na_2PdCl_4 (subject to equilibration as described in previous section), 100 µL of 100 mM HNO_3, 60 µL of 0.1 mM freshly-prepared NaI, and 230 µL neat acetone to a 20 mL scintillation vial. The vial was placed in a 40 °C water bath and allowed to come to temperature for at least 10 minutes. To this warm solution, 100 µL of 10x diluted nanocube seeds (seeds diluted in 12.5 mM BioXtra CTAB) was added, and the solution swirled vigorously. 100 µL of fresh 100 mM AA was injected, and the solution was swirled again. The vial was then put back into the 40 °C water bath for at least 15 hours. This procedure was followed for syntheses with OCP measurements, with the following two exceptions: (1) for both potentiostat OCP and ORP sensor measurements, swirling of the growth solution was replaced by 200 RPM magnetic stirring and (2) potentiostat OCP measurements only were conducted in a five-neck flask (Gamry Dr. Bob's Cell), rather than a 20 mL vial.

2.10. Colloidal Synthesis of Monometallic Au Nanoparticles

Au nanoparticles were synthesized via modification of a previously reported method.[7–9] A growth solution was prepared by adding 10 mL of 100 mM room-temperature CTAC (Sigma 25 wt. % solution), 500 µL of 10 mM $HAuCl_4$, and 200 µL of 1 M HCl to a 20 mL scintillation vial and swirling vigorously. 100 µL of 100 mM fresh AA was added and the vial was swirled again. 100 µL of 1000x diluted Au seed particles (in 100 mM Sigma 25 wt. % CTAC) were added to the growth solution. The reaction was swirled immediately after the addition of the seeds and then left undisturbed on the bench top for at least 30 minutes. This procedure was followed for syntheses with OCP measurements, with the following two exceptions: (1) for both potentiostat OCP and

ORP sensor measurements, swirling of the growth solution was replaced by 200 RPM magnetic stirring and (2) potentiostat OCP measurements only were conducted in a five-neck flask (Gamry Dr. Bob's Cell), rather than a 20 mL vial.

2.11. Colloidal Synthesis of Shape-Controlled (Ag)Au Nanoparticles in As-Received Sigma CTAC

(Ag)Au nanoparticles were synthesized using a previously reported method.[7,9] A growth solution was prepared by adding 10 mL of 100 mM room-temperature CTAC (Sigma 25 wt. % solution), 500 µL of 10 mM $HAuCl_4$, 200 µL of 1 M HCl, and x µL of 10 mM freshly-prepared $AgNO_3$ (x = 1 µL for octahedra, 10 µL for rhombic dodecahedra, 40 µL for truncated ditetragonal prisms, and 100 µL for concave cubes) to a 20 mL scintillation vial and swirling vigorously. 100 µL of 100 mM fresh AA was added and the vial was swirled again. 100 µL of 1000x diluted Au seed particles (in 100 mM Sigma 25 wt. % CTAC) were added to the growth solution. The reaction was swirled immediately after the addition of the seeds and then left undisturbed on the bench top for ~180 minutes. This procedure was followed for syntheses with OCP measurements, with the following two exceptions: (1) for both potentiostat OCP and ORP sensor measurements, swirling of the growth solution was replaced by 200 RPM magnetic stirring and (2) potentiostat OCP measurements only were conducted in a five-neck flask (Gamry Dr. Bob's Cell), rather than a 20 mL vial.

2.12. CTAC/NaAsc Synthesis of Homogeneously Nucleated Cu Nanocubes

Homogeneously nucleated Cu cubic nanoparticles were synthesized via modification of a previously reported method.[10] In a 20 mL scintillation vial, a growth solution was prepared by adding 9.775 mL of 15 mM CTAC (Sigma 25 wt% solution), 75 µL of 100 mM $Cu(OAc)_2$ and a stir bar (3 x 10 mm). The solution was stirred at 750 rpm above a 90 °C silicone oil bath. 170 µL of a 500 mM NaAsc solution was added, and the mixture was stirred at 750 rpm for 1 min at room

temperature. Thereafter, the vial was lowered into the silicone oil bath and the solution was stirred at 60 rpm for 40 min. An analogous procedure was followed for syntheses with OCP measurements, but 3D printed vial caps with openings for insertion of either the WE and RE (potentiostat OCP) or ORP sensor were used rather than standard vial caps.

2.13. HDA/AA Synthesis of Pd-Seeded Cu Nanocubes

Seeded Cu nanocubes were synthesized via modification of a previously reported method.[6] 60 mg HDA, 9.1 mL ultrapure DI water, 0.5 mL of 100 mM $CuCl_2$ and a stir bar (3 x 10 mm) were added in a 20 mL scintillation vial, sonicated, and vigorously stirred overnight. Afterwards, the solution was stirred at 600 rpm at room temperature, and 120 µL of undiluted ~21 nm Pd nanocube seed solution was added to the mixture and further stirred for 5 min before 200 µL of 1 M AA was added. The mixture was immediately placed in a 100 °C silicone oil bath for 60 min under continuous stirring at 600 rpm. An analogous procedure was followed for syntheses with OCP measurements, but 3D printed vial caps with openings for insertion of either the WE and RE (potentiostat OCP) or ORP sensor were used rather than standard vial caps.

2.14. Colloidal Synthesis of Shape-Controlled (Ag)Au Nanoparticles in As-Received TCI CTAC

*(*Ag)Au nanoparticles were synthesized similarly to the (Ag)Au nanoparticles in as-received Sigma CTAC (see 2.11). However, 10 mL of 100 mM room-temperature as-received TCI CTAC (TCI powder in ultrapure DI water) was used instead of 10 mL of 100 mM room-temperature Sigma CTAC for the growth solutions and seed dilutions.

2.15. Colloidal Synthesis of Shape-Controlled (Ag)Au Nanoparticles in Recrystallized TCI CTAC

*(*Ag)Au nanoparticles were synthesized by adapting a previously reported method.[7,9] A growth solution was prepared by adding 10 mL of 100 mM room-temperature CTAC (recrystallized, oven-

dried TCI), 500 μL of 10 mM $HAuCl_4$, 200 μL of 1 M HCl, 50 μL of neat acetone (or 44 μL of neat ethanol, 30 μL of neat methanol, 58 μL of isopropanol, or 60 μL of neat chloroform. 10 μL of 0.1 mM NaI and x μL of 10 mM freshly-prepared $AgNO_3$ (x = 1 μL for octahedra, 10 μL for rhombic dodecahedra, 40 μL for truncated ditetragonal prisms, and 100 μL for concave cubes) to a 20 mL scintillation vial and swirling vigorously. The vial was kept capped whenever reagents were not being injected to avoid evaporation of the organic solvent additive prior to synthesis. 100 μL of 100 mM fresh AA was added and the vial was swirled again. 100 μL of 1000x diluted Au seed particles (diluted in 100 mM recrystallized, dried TCI CTAC) were added to the growth solution. The reaction was swirled immediately after the addition of the seeds and then left undisturbed on the bench top for ~180 minutes. This procedure was followed for syntheses with OCP measurements, with the following two exceptions: (1) for both potentiostat OCP and ORP sensor measurements, swirling of the growth solution was replaced by 200 RPM magnetic stirring and (2) potentiostat OCP measurements only were conducted in a five-neck flask (Gamry Dr. Bob's Cell), rather than a 20 mL vial.

2.16. Colloidal Synthesis of Shape-Controlled (Ag)Au Nanoparticles in Lyophilized, Recrystallized Sigma CTAC

(Ag)Au nanoparticles in lyophilized, recrystallized Sigma CTAC were synthesized similarly to the (Ag)Au nanoparticles in recrystallized TCI CTAC (see 2.15). However, 10 mL of 100 mM room-temperature lyophilized, recrystallized Sigma CTAC (Sigma 25 wt. %; lyophilized and vacuum oven-dried, then recrystallized and oven-dried; in ultrapure DI water) was used instead of 100 mM room-temperature recrystallized TCI CTAC (recrystallized, oven-dried TCI powder in ultrapure DI water) for the growth solutions and seed dilutions.

3. *Electroanalytical Methods*

3.1. Electrode Preparation and Reference Electrode Benchmarking for Potentiostat OCP

Potentiostat open-circuit potential (OCP) measurements were taken using a polished GCE (disk insert or fixed disk), a polished Pt fixed disk (unoxidized or oxidized in air), or other Pt electrode (wire or mesh) as the WE.

GCEs (5 mm OD x 4 mm thick disk insert, Pine Research) used for measurements of Pd and Au nanoparticle growth were prepared by polishing on MasterTex Buehler polishing pad with a slurry of 0.3 µM alumina polishing powder (Buehler MicroPolish Alumina), rinsed with ultrapure DI water, polished again with 0.05 µm alumina polishing powder (Buehler MicroPolish Alumina) on a different polishing pad and rinsed again. Polished electrodes were then sonicated in a 20 mL scintillation vial in ultrapure DI water for 15 minutes three times (with water changed after each sonication step to remove alumina) and rinsed with ultrapure DI water before being inserted into a Teflon electrode tip holder (Pine Research). Once assembled, the electrode in the Teflon tip holder was rinsed and dried again. For the OCP measurements of the Cu nanoparticle growth, fixed GCEs (6.35 mm OD, Pine Research) were used. These were polished similarly to the disk insert WEs. However, the sonication step at the end was substituted with a vigorous ultrapure DI washing.

Fixed disk Pt electrodes (3 mm OD, Gamry) used as WEs for measurement of Pd or Au particle synthesis were polished identically to fixed disk GCEs. They were either immediately introduced to the growth solution for OCP measurement following polishing and washing (standard procedure) or set on the benchtop in ambient atmosphere for ≥ 2 hours to promote the formation of a Pt oxide layer ("oxidized Pt disk WE"). Pt wires (0.406 mm diameter x 15 cm (6 in) length, Gamry) and Pt mesh (100 mesh woven from 0.0762 mm (0.003 in) diameter wire, 25 x 25 mm,

Thermo) used as WEs for measurement of Pd or Au particle synthesis were used as received and not subject to cleaning procedures other than rinsing between uses and dipping rapidly in aqua regia (1:3 ratio of concentrated HNO_3: concentrated HCl; **caution: strong acid**) after use in Au syntheses to remove Au plating. Pt wires used as WEs in the measurement of Cu nanoparticle growth were dipped in concentrated HNO_3 (**caution: strong acid**) between uses.

The following REs were used depending on the reaction: Ag/AgCl (saturated KCl electrolyte, Koslow Scientific Co.), low-profile Ag/AgCl (4 M KCl gel, Pine Research), leakless Ag/AgCl (3.4 M KCl, eDAQ), Hg/Hg_2SO_4 (saturated K_2SO_4 electrolyte, Koslow Scientific Co.) or a reversible hydrogen electrode (RHE, HydroFlex, Gaskatel). Ag/AgCl REs were used for measurements of the Pd corrugated particle synthesis. Hg/Hg_2SO_4 REs were generally used to measure the Pd THH synthesis, to avoid formation of a AgI layer on Ag/AgCl REs.[1] Hg/Hg_2SO_4 REs were used for all measurements of Au particle synthesis, except when otherwise noted, to avoid leakage of Ag^+ ions,[11] which affect Au particle synthesis at very dilute concentrations.[9] Low-profile Ag/AgCl REs were used for measurements of the HDA/AA seeded Cu particle synthesis. RHE REs were used for the measurement of the CTAC/NaAsc homogeneously nucleated Cu nanocube synthesis.

For both Pd and Au particle synthesis, the RE was isolated from the reaction solution by a bridge tube with glass frit containing 100 mM H_2SO_4 to stop leakage of Hg or Ag ions and to prevent damage to the RE. Low-profile and leakless Ag/AgCl REs were directly inserted into the Cu nanoparticle growth solution, to avoid the glassware sensitivities of the reaction. RHE REs were directly inserted into the growth solution.

REs were stored at room temperature, away from direct sunlight. Ag/AgCl electrodes were kept in saturated potassium chloride storage solution (KCl, solution in water with potassium

hydrogen phthalate, Thermo Fisher Scientific Accumet Electrode Storage Solution), and Hg/Hg_2SO_4 REs were kept in saturated K_2SO_4 storage solution at all times when not in use. All REs were labeled and each electrode was stored in a corresponding dedicated vial of storage solution. Saturated Hg/Hg_2SO_4 REs were prone to the formation of salt crystals near the porous frit, preventing appropriate ion exchange. These crystals were periodically eliminated by placing a drop of ~50 °C ultrapure DI water in the K_2SO_4 storage solution container and/or placing the RE (in the storage solution) in a 30 °C water bath for 30-60 minutes. RHEs were stored at room temperature in ultrapure DI water. After each use, RHEs were cleaned by dipping in conc. HNO_3 (**Caution: strong acid)** for ~1 s and washing with ultrapure DI water.

Ag/AgCl and Hg/Hg_2SO_4 REs were benchmarked (in bridge tubes, in 0.1 M H_2SO_4 electrolyte solution) against a "master" Ag/AgCl RE. These tests showed a variance between all Ag/AgCl REs tested of ± 0.008 V and between all Hg/Hg_2SO_4 REs tested of ± 0.003 V across an 1800-second benchmarking measurement. RHEs were benchmarked against a Hg/Hg_2SO_4 RE in 1 M HCl, with a variance of the measured electrodes tested of ± 0.004 V across 1200-second benchmarking measurements. To ensure an absence of a reference shift of the low-profile Ag/AgCl electrode after repeated use without a bridge tube (and to avoid contamination of any of the bridge tubes or master electrodes) the low-profile Ag/AgCl REs were tested by measuring the OCP of a 200 mV ORP test solution (GLI International/Hach) with a Pt wire as the WE after every two uses.

Measured solution potentials were converted to the standard hydrogen electrode (SHE) scale to streamline comparison between measurements by adding 0.197 V to all Ag/AgCl electrode measurements and adding 0.640 V to all Hg/Hg_2SO_4 electrode measurements of ambient temperature and 40 °C syntheses. To account for the increased temperature at which the low-profile Ag/AgCl REs were used, the RE potential in the seeded Cu particle growth experiments

was converted by adding 0.162 V based on the following equation for the temperature dependence of Ag/AgCl REs vs SHE:[12]

$$E^{\circ}_{\mathrm{Ag/AgCl}}(V)\ \mathrm{vs\ SHE} = 0.23695 - 4.8564 * 10^{-4} * T(℃) - 3.4205 * 10^{-6} * T^{2}(℃) - 5.869 * 10^{-9} * T^{3}(℃)$$

The growth solution temperature was approximated as 90 °C throughout the whole reaction.

The measured potentials vs RHE potentials were transformed to potentials vs SHE with the following formula:[13]

$$E_{\mathrm{vs\ SHE}} = E_{\mathrm{vs\ RHE}} - 2.303 \cdot \left(\frac{R \cdot T}{F}\right) \cdot \mathrm{pH}$$

$E_{\mathrm{vs\ SHE}}$: Measured potential reported vs SHE

$E_{\mathrm{vs\ RHE}}$: Measured potential vs RHE

R: Universal gas constant

T: Temperature in Kelvin

F: Faraday constant

The pH during the synthesis was measured similarly to the OCP and ORP measurements of the reaction using a RHE-based pH electrode (pHydrunio, Gaskatel). The measured potential was transformed to a pH value via the following equation:

$$\mathrm{pH} = 7 - \frac{E_{\mathrm{meas}}}{2.303 \cdot \frac{R \cdot T}{F}}$$

E_{meas}: Measured potential with pHydrunio electrode

R: Universal gas constant

T: Temperature in Kelvin

F: Faraday constant

The temperature used for both the pH as well as the RHE electrode was measured by inserting a handheld thermometer (VWR, Traceable Alarm Thermometer/Alarm Timer) while measuring the OCP of the homogeneously nucleated Cu cube synthesis using a GCE and RHE with a 100 °C silicone oil bath. After 15 min of measurement, the temperature was assumed to be constant and the last measured temperature value was used for the remainder of the reaction.

3.2. Working Electrode Surface Area Calculations

WE dimensions (disk diameter, wire diameter and length, or band height and diameter) were measured with calipers (Johnson 6" digital caliper, Model No. 1889-0600; 0.01 mm resolution). Of note, the measured length of the Pt wire WE was based on the portion of the wire immersed in growth solution during measurement, not the total length of the wire. The following formulas were used to calculate WE surface areas:

Disk WE (potentiostat OCP with GCE):

$$SA = \pi r_{disk}^2$$

Wire WE (potentiostat OCP with Pt wire WE; DJ epoxy ORP):

$$SA = 2\pi r_{wire} l_{wire} + \pi r_{wire}^2$$

Band WE (SJ epoxy ORP; DJ glass ORP):

$$SA = 2\pi r_{band} h_{band}$$

Mesh WE (potentiostat OCP with Pt mesh WE; square piece of mesh):

(1) $ap = \frac{1}{mesh} - d_{in}$

(2) $A = lh$

(3) $n_{wire} = \frac{A}{ap}$

(4) $SA = n_{wire}^2(2\pi r_{wire} ap)$

SA: surface area (mm^2)

d: diameter (mm)

d_{in}: diameter in inches (in)

r: radius (equal to $\frac{1}{2}$ of measured diameter; mm)

l_{wire}: measured length of wire WE (mm)

h_{band}: measured height of band WE (mm)

ap: aperture size (opening size) for mesh (inches; converted to mm for further calculations; equal to length of individual wire segment in mesh)

$mesh$: mesh count (threads per linear inch)

A: outer dimensions of mesh (area of a rectangle; mm^2)

n_{wire}: number of wire segments in one linear direction in mesh

n_{wire}^2: total number of wire segments in a square piece of mesh

3.3. Cleaning of Electrochemistry Glassware

Electrochemistry glassware, including 5-neck heart-shaped flasks (Gamry Dr. Bob's Cell), glass bridge tubes for REs (Gamry), miniature stir bars (3 x 10 mm), glass stoppers, and Teflon holders (Pine Research) for glassy carbon disk electrode inserts were cleaned with aqua regia (1:3 ratio of concentrated HNO_3: concentrated HCl; **caution: strong acid**) between each use. Electro-porous KT glass frits (Gamry or Koslow Scientific) on bridge tubes (Gamry) were replaced every ~8 weeks or whenever they became permanently discolored. Bridge tubes were used with only one type of metal ion in the growth solution and one type of RE between cleanings with aqua regia and/or frit replacement, to avoid contamination. Bridge tubes were stored with the frits immersed

in 100 mM H_2SO_4, which was changed every time the bridge tubes were cleaned with aqua regia. Electrochemistry flasks that were used for syntheses that contain chloride only; chloride and bromide; or chloride, bromide, and iodide were kept separate, as adsorbed halides were challenging to remove from the glassware. Glassware that had contained bromide or iodide was washed in 50 °C ultrapure DI water, which removed halide residues more successfully than room-temperature water.

3.4. Calibration, Cleaning and General Care of ORP Sensors

For calibration of the ORP sensor, the two-point linear calibration function in the Vernier Graphical Analysis program was used. The ORP sensor was washed with ultrapure DI water, dried, and inserted first into the 600 mV calibration solution (ORP test solution, 600 mV, GLI International/Hach) until the potential stabilized. Afterwards, the ORP sensor was thoroughly washed with ultrapure DI water and inserted into the 200 mV calibration solution (ORP test solution, 200 mV, GLI International/Hach) until the potential stabilized and then washed again.

After use for the measurement of Pd nanoparticle growth, the ORP sensor was triple rinsed with ultrapure DI water and wiped with a Kimwipe. After use for the measurement of Au or (Ag)Au nanoparticle growth at the end of the reaction, the ORP sensor was rinsed with ultrapure DI water and was cleaned by quickly dipping in aqua regia (1:3 ratio of concentrated HNO_3: concentrated HCl; **caution: strong acid**) three times for ~five seconds and rinsing with ultrapure DI water. Before the next use the ORP sensor was allowed to equilibrate in storage solution for at least 30 min. Following use for the measurement of Cu nanoparticle growth, the ORP sensor was rinsed with ultrapure DI water and was cleaned by quickly dipping in conc. HNO_3 (**caution: strong acid**) three times for ~five seconds and rinsing with DI. ORP sensors were cleaned after each use, as shifts in the measured potential of the calibration solutions (and the subsequent measured

growth reaction) could be observed after a single use (Au) or after multiple consecutive uses (Pd, Cu) without cleaning.

Following cleaning, the ORP sensor was allowed to equilibrate in storage solution for at least 30 min. Equilibration of the ORP sensors after cleaning was needed to ensure that the potential reading during the calibration step was in the expected range.

For all experiments with replicates, at least two different ORP sensors of the same model were used to ensure reproducibility.

Criteria used to determine the reliability of the ORP sensors included their response time and the measured potential of the calibration solutions when calibrating the sensor, with slow response time (delay in change when the sensor was introduced to a new calibration solution) and unusual potential readings indicating unreliability. The reliable lifetime of each ORP sensor depended heavily on the measured growth system, and a general recommendation on the maximum number of uses can't be given, but ORP sensors used in higher temperature reactions (i.e. Cu) generally had decreased lifetimes. Immersion in concentrated acid for extended periods (rather than quickly dipping in aqua regia to clean) also decreased the reliability and lifetime of the sensor.

3.5. Potentiostat Open-Circuit Potential Measurements of Colloidal Pd Corrugated Particle Synthesis

Potentiostat OCP measurements of the Pd corrugated particle synthesis were performed in a five-neck electrochemistry flask (Gamry Dr. Bob's Cell). The measurements used an Ag/AgCl RE (Koslow) in a bridge tube containing 100 mM H_2SO_4 and a disk insert GCE (Pine). No CE was used. A growth solution of 10 mL of 12.5 mM Sigma CTAC, 500 µL of 10 mM Na_2PdCl_4, and 30 µL of 1 M HNO_3 was prepared in the flask. A 3 x 10 mm stir bar was added and 200 RPM stirring was initiated. Before the addition of seeds and or reducing agent, the electrodes were placed into the growth solution and the leads connected to the potentiostat (Gamry Instruments 1010E). After

waiting for ~five minutes, the OCP measurement was started using the Gamry Framework software interface.

Growth was initiated via the injection of 100 µL of 100 mM AA, followed by the injection of a 100 µL aliquot of a solution of small pseudospherical Pd seeds (seeds diluted 10000x in 12.5 mM as-received BioXtra CTAB). Stirring was turned off 10 seconds after the addition of Pd seeds. The OCP of the growth solution was measured for 30 minutes. Electrodes were removed and rinsed with ultrapure DI water.

3.6. ORP Sensor Open-Circuit Potential Measurements of Colloidal Pd Corrugated Particle Synthesis

ORP sensor measurements were collected using Vernier Graphical Analysis software. ORP measurements of the colloidal Pd corrugated particle synthesis were carried out analogously to the potentiostat measurements; however, instead of an RE and a WE, the ORP sensor (Vernier Science Education, ORP Sensor, ORP-BTA ("SJ Epoxy ORP") or Cole-Parmer, ORP electrode, double-junction, platinum band, 10-ft cable, EE-27006-21 ("DJ glass ORP")) was inserted directly into the growth solution. The ORP sensor and electrode amplifier pair were connected to the instrument computer using a Go!Link USB interface (Vernier). Prior to use, the ORP sensor and electrode amplifier pair was calibrated, as previously described. After successful calibration, the ORP sensor was inserted into the growth solution, containing 10 mL of 12.5 mM Sigma CTAC, 500 µL of 10 mM Na_2PdCl_4, 30 µL of 1 M HNO_3 and a 3 x 10 mm stir bar. 200 rpm stirring was initiated. After the measured potential stabilized, the ORP measurement was started. 100 µL of 100 mM AA was added, followed by the injection of 100 µL of small pseudospherical Pd seed suspension (seeds 10000x diluted in 12.5 mM as-received BioXtra CTAB). Stirring was turned off 10 seconds after the addition of diluted seeds, and the measurement was continued for 30 minutes. At the end of the reaction, the ORP sensor was removed and rinsed thoroughly with ultrapure DI water.

3.7. Potentiostat Open-Circuit Potential Measurements of Colloidal Pd THH Synthesis

Potentiostat OCP measurements of the Pd THH synthesis were performed in a five-neck electrochemistry flask (Gamry Dr. Bob's Cell). The measurements used an Ag/AgCl RE (Koslow) in a bridge tube containing 100 mM H_2SO_4 and a disk insert GCE (Pine), a Pt wire (Gamry), or a Pt fixed disk (Gamry) as the WE. No CE was used in the experiments. A growth solution of 10 mL of 50 mM CTAB (oven-dried BioXtra), 200 µL of 10 mM Na_2PdCl_4 (subject to equilibration as previously described), 100 µL of 0.1 M HNO_3, 60 µL of 0.1 mM freshly prepared NaI, and 230 µL neat acetone was prepared in the flask in a 40 °C water bath. A 3 x 10 mm stir bar was added and 200 RPM stirring was initiated. Before the addition of seeds and or reducing agent, the electrodes were placed into the growth solution and the leads connected to the potentiostat (Gamry Instruments 1010E). After waiting for ten minutes, the OCP measurement was started.

Growth was initiated via the injection of 100 µL of Pd nanocube seed suspension (seeds 10x diluted in 12.5 mM BioXtra CTAB), followed by the injection of 100 µL of 100 mM AA. Stirring was turned off 10 seconds after the addition of AA. The OCP of the growth solution was measured for 30+ minutes. Electrodes were removed and rinsed with ultrapure DI water.

3.8. ORP Sensor Open-Circuit Potential Measurements of Colloidal Pd THH Synthesis

ORP sensor measurements were collected using Vernier Graphical Analysis software. ORP measurements of Pd THH nanoparticle synthesis were carried out analogously to the potentiostat measurements; however, instead of an RE and a WE, the ORP sensor (Vernier Science Education, ORP Sensor, ORP-BTA ("SJ Epoxy ORP"), Cole-Parmer, ORP electrode, double-junction, platinum band, 10-ft cable, EE-27006-21 ("DJ glass ORP")) was inserted into the growth solution. A growth solution was prepared in a scintillation vial, containing 10 mL of 50 mM oven-dried BioXtra CTAB, 200 µL of 10 mM Na_2PdCl_4 (subject to equilibration as previously described), 100

μL of 0.1 M HNO_3, 60 μL of 0.1 mM freshly-prepared NaI, 230 μL neat acetone, and a 3 x 10 mm stir bar. The vial was clamped in a 40 °C water bath and 200 RPM stirring was initiated. The ORP sensor and electrode amplifier pair were connected to the instrument computer using a Go!Link USB interface (Vernier). Prior to use, the ORP sensor and electrode amplifier pair was calibrated, as previously described. After successful calibration, the ORP sensor was directly inserted into the growth solution. After the measured potential stabilized, the ORP measurement was started, while the solution was stirred at 200 rpm. Growth was initiated via the injection of 100 μL of Pd nanocube seed suspension (seeds 10x diluted in 12.5 mM BioXtra CTAB), followed by the injection of 100 μL of 100 mM AA. Stirring was turned off 10 seconds after the addition of AA, and the measurement was continued for 30 minutes. At the end of the reaction, the ORP sensor was removed and rinsed thoroughly with ultrapure DI water.

3.9. Potentiostat Open-Circuit Potential Measurements of Colloidal Au and (Ag)Au Particle Synthesis

Potentiostat OCP measurements of Au and (Ag)Au particle syntheses were performed in a five-neck electrochemistry flask (Gamry Dr. Bob's Cell). The measurements used a Hg/Hg_2SO_4 RE (Koslow) in a bridge tube containing 100 mM H_2SO_4, unless otherwise noted, and a disk insert GCE (Pine), a Pt wire (Gamry), a Pt fixed disk (Gamry), Pt mesh (Thermo Scientific) as the WE. No CE was used in the experiments. A growth solution was prepared in the five-neck flask, containing 10 mL of 100 mM CTAC (see synthesis section for discussion of CTAC sources), 500 μL of 10 mM $HAuCl_4$, 200 μL of 1 M HCl, NaI if applicable (see synthesis section), acetone or other organic solvent if applicable (see synthesis section) and x μL of 10 mM freshly prepared $AgNO_3$ (at standard conditions, x = 0 μL for monometallic Au particles, 1 μL for octahedra, 10 μL for rhombic dodecahedra, 40 μL for truncated ditetragonal prisms, and 100 μL for concave cubes). A 3 x 10 mm stir bar was added and 200 RPM stirring was initiated. Before the addition

of seeds and or reducing agent, the electrodes were placed into the growth solution and the leads connected to the potentiostat (Gamry Instruments 1010E). After waiting for ~5 minutes, the OCP measurement was started using the Gamry Framework software interface.

Growth was initiated via the injection of 100 μL of 100 mM AA, followed by the injection of 100 μL of Au seed suspension (seeds 1000x diluted in 100 mM Sigma CTAC). Stirring was turned off 10 seconds after the addition of Au seeds. The OCP of the growth solution was measured for 30 minutes unless otherwise noted. Electrodes were removed and rinsed with ultrapure DI water.

3.10. ORP Sensor Open-Circuit Potential Measurements of Colloidal Au and (Ag)Au Particle Synthesis

ORP sensor measurements were collected using Vernier Graphical Analysis software. ORP measurements of Au nanoparticle synthesis were carried out analogously to the potentiostat measurements; however, instead of an RE and a WE, the ORP sensor (Vernier Science Education, ORP Sensor, ORP-BTA ("SJ Epoxy ORP") or Cole-Parmer, ORP electrode, double-junction, platinum band, 10-ft cable, EE-27006-21 ("DJ glass ORP") was inserted into the growth solution. The ORP sensor and electrode amplifier pair were connected to the instrument computer using a Go!Link USB interface (Vernier). Prior to use, the ORP sensor and electrode amplifier pair was calibrated, as previously described. A growth solution was prepared in a scintillation vial , containing a 3 x 10 mm stir bar, 10 mL of 100 mM CTAC (see synthesis section for discussion of CTAC sources), 500 μL of 10 mM $HAuCl_4$, 200 μL of 1 M HCl, NaI if applicable (see synthesis section), acetone or other organic solvent if applicable (see synthesis section) and x μL of 10 mM freshly prepared $AgNO_3$ (at standard conditions, x = 0 μL for monometallic Au particles, 1 μL for octahedra, 10 μL for rhombic dodecahedra, 40 μL for truncated ditetragonal prisms, and 100 μL for concave cubes). After successful calibration, the ORP sensor was directly inserted into the

growth solution. After the measured potential stabilized, the ORP measurement was started, while the solution was stirred at 200 rpm. 100 µL of 100 mM AA was added, followed by the injection of 100 µL of Au seed suspension (seeds 1000x diluted in 100 mM Sigma CTAC). Stirring was turned off 10 seconds after the addition of diluted seeds, and the measurement was continued for 30 minutes. At the end of the reaction, the ORP sensor was removed, rinsed with ultrapure DI water, and cleaned by quickly dipping in aqua regia (1:3 ratio of concentrated HNO_3: concentrated HCl; **caution: strong acid**) and rinsing with ultrapure DI water.

3.11. Potentiostat Open-Circuit Potential Measurements of CTAC/NaAsc Homogeneously Nucleated Cu Nanocubes

Potentiostat OCP measurements of the homogeneously nucleated Cu cubic nanoparticles were performed directly in the 20 mL scintillation vials used for the unmeasured colloidal synthesis. A growth solution was prepared, consisting of 9.775 mL of 15 mM CTAC (Sigma 25 w% solution), 75 µL of 100 mM $Cu(OAc)_2$ and a stir bar (3 x 10 mm), through openings in a 3D-printed vial cap. A reversible hydrogen electrode (RHE, HydroFlex, Gaskatel) or a leakless Ag/AgCl RE (3.4 M KCl, eDAQ) was used as the RE, and directly inserted into the growth solution through openings in the 3D-printed vial cap. Either the fixed disk GCE or a Pt wire was used as the WE. Before the addition of reducing agent, the electrodes were placed into the growth solution and the leads connected to the potentiostat (Gamry Instruments 1010E). When the potential stabilized (after ~5 minutes), the OCP measurement was started using the Gamry Instruments Software interface, while the solution was stirred at 750 rpm above a 90 °C silicone oil bath. 170 µL of a 500 mM NaAsc solution was added, and the mixture was stirred at 750 rpm for 1 min at room temperature. After this, the vial was lowered into the silicone oil bath and the solution was stirred at 60 rpm for 40 min. At the end of the reaction, the electrodes were removed and rinsed with ultrapure DI water and cleaned. To ensure reproducible results, the first measurement after not using the RHE for ≥15

hours was discarded, as it did not show the same responsiveness to potential changes at early stages of the reactions. After the first run, all the following measurements were in good agreement with each other. The measured potentials vs RHE were transformed to potentials vs SHE as described previously.

3.12. ORP Sensor Open-Circuit Potential Measurements of CTAC/NaAsc Homogeneously Nucleated Cu Nanocubes

ORP sensor measurements were collected using Vernier Graphical Analysis software. The ORP measurements were carried out analogously to the potentiostat measurements; however, instead of an RE and a WE, the ORP sensor (Cole-Parmer, ORP electrode, double-junction, platinum band, 10-ft cable, EE-27006-21 ("DJ glass ORP") or Vernier Science Education, ORP Sensor, ORP-BTA ("SJ Epoxy ORP")) was inserted into the growth solution. The ORP sensor and electrode amplifier pair were connected to the instrument computer using a Go!Link USB interface (Vernier). Prior to use the ORP sensor and electrode amplifier pair was calibrated, as previously described. After successful calibration, the ORP sensor was directly inserted into the growth solution, consisting of 9.775 mL of 15 mM CTAC (Sigma 25 w% solution), 75 µL of 100 mM $Cu(OAc)_2$ and a stir bar (3 x 10 mm), through openings in a 3D-printed vial cap. After the measured potential stabilized, the ORP measurement was started, while the solution was stirred at 750 rpm above a 90 °C (standard condition) or 100 °C silicone oil bath. 170 µL of a 500 mM NaAsc solution was added, and the mixture was stirred at 750 rpm for 1 min at room temperature. After this, the vial was lowered into the silicone oil bath and the solution was stirred at 60 rpm for 40 min. At the end of the reaction, the ORP sensor was removed, rinsed with ultrapure DI water, and cleaned by quickly dipping in conc. HNO_3 (**caution: strong acid**) and rinsing with ultrapure DI water.

The RE potential of the ORP sensor's Ag/AgCl RE was corrected for the temperature and adjusted to SHE as described previously. The growth solution temperature was approximated as 90 °C throughout the whole reaction for all conditions.

3.13. Potentiostat Open-Circuit Potential Measurements of HDA/AA Pd-Seeded Cu Nanocubes

Potentiostat OCP measurements of the Pd-seeded Cu cube synthesis were performed directly in the 20 mL scintillation vials used for the unmeasured colloidal synthesis. A low-profile Ag/AgCl RE (Silver/Silver Chloride, Low-Profile, 60 mm long, Pine Research) was directly inserted into the growth solution through openings in a 3D-printed vial cap. Either the fixed disk GCE (Pine) or a Pt wire (Gamry) was used as the WE. No CE was used in the experiments. Before the addition of seeds and or reducing agent, the electrodes were placed into the growth solution and the leads connected to the potentiostat (Gamry Instruments 1010E). After waiting for ~five minutes until the potential stabilized, the OCP measurement was started using the Gamry Instruments Software interface.

The synthesis was started by adding 120 μL of undiluted 21 nm Pd cube seed solution to the growth solution, and the mixture was stirred at 600 rpm for 5 min before adding 200 μL of 1 M AA. Immediately, the vial was lowered into a 100 °C silicone oil bath and the solution was stirred at 600 rpm for 1 hour. At the end of the reaction, the electrodes were removed and rinsed with DI. The Pt wire and the low-profile Ag/AgCl RE were cleaned by quickly dipping in conc. HNO_3 (**caution: strong acid**) and rinsing with DI. The RE potential of the Ag/AgCl RE was corrected for the temperature and adjusted to SHE as previously described.

3.14. ORP Sensor Open-Circuit Potential Measurements of HDA/AA Pd-Seeded Cu Nanocubes

ORP sensor measurements were collected using Vernier Graphical Analysis software. The ORP measurements were carried out analogously to the potentiostat measurements; however, instead of an RE and a WE, the ORP sensor (Cole-Parmer, ORP electrode, double-junction, platinum band, 10-ft cable, EE-27006-21 ("DJ glass ORP")) was inserted into the growth solution. The ORP sensor and electrode amplifier pair were connected to the instrument computer using a Go!Link USB interface (Vernier). Prior to use the ORP sensor and electrode amplifier pair was calibrated, as previously described. After successful calibration, the ORP sensor was directly inserted into the growth solution through openings in a 3D-printed vial cap. After the measured potential stabilized, the ORP measurement was started, while the growth solution remained at ambient temperature. The synthesis was started by adding 120 µL of undiluted 21 nm Pd cube seed solution to the growth solution. The mixture was stirred at 600 rpm for 5 min, before adding 200 uL of 1 M AA. Immediately, the vial was lowered into a 100 °C silicone oil bath and the solution was stirred at 600 rpm for 1 hour. At the end of the reaction, the ORP sensor was removed, rinsed with ultrapure DI water, and cleaned by quickly dipping in conc. HNO_3 (**caution: strong acid**) and rinsing with ultrapure DI water.

The RE potential of the Ag/AgCl RE was corrected for the temperature and adjusted to SHE scale as described previously. The growth solution temperature was approximated as 90 °C throughout the whole reaction.

4. ***Other Methods***

4.1. Electron Microscopy Characterization

To prepare colloidal samples for characterization via scanning electron microscopy (SEM), the completed reaction vial was vortexed, sonicated for ~30 seconds, and vortexed again. From this solution, 1 mL was removed and transferred to a microcentrifuge tube. The solution was then centrifuged (VWR Micro Star 21 or Eppendorf 5430) at 6000 rpm for four minutes to spin down a pellet. The supernatant was removed with a Pasteur pipette, and the microcentrifuge tube refilled to 1 mL with ultrapure DI water, vortexed, sonicated, vortexed, and then centrifuged again at 6000 rpm for four minutes. The supernatant was removed again, and a minimal amount of ultrapure DI water (200-400 µL) was added, and the tube was sonicated and vortexed to disperse the particles. 1 µL of this solution was then dropcast onto a prepared silicon wafer and allowed to dry for at least 45 minutes in a desiccator before characterization by SEM (FEI Quanta 650 FE-SEM). The Pd-seeded Cu nanocubes were washed by refilling with 1 mL of ethanol instead of DI water and the washing step was repeated three times overall instead of only once. Au, (Ag)Au, and Cu nanoparticles were imaged within 96 hours of sample preparation due to oxidative etching of dried particles; etching of Pd nanoparticle SEM samples was not observed. Average particle size and shape yield were determined using >100 particles using ImageJ. Brightness and contrast of electron microscopy images were normalized using the Curves adjustment tool in Adobe Photoshop and Adobe Photoshop Elements 2024.

4.2. ^{1}H-Nuclear Magnetic Resonance (NMR) Characterization of CTAC

NMR spectra were recorded on a Bruker Avance III 600 spectrometer (600 MHz, ^{1}H). The spectrum of as-received Sigma CTAC (25 wt% solution in water) was taken in a 9:1 mixture of ultrapure DI water and D_2O. All other samples were dissolved in D_2O to a 100 mM concentration,

and all were vortexed prior to spectra being taken. All spectra were obtained at room temperature. All NMR tubes were cleaned thoroughly with ultrapure DI water and methanol, and oven-dried at 100 °C overnight before use. NMR data was processed using MestReNova v14.3.3-33362.

4.3. ICP-OES Sample Preparation and Analysis

For Au growth solutions, samples were prepared by removing a 1 mL aliquot from relevant solutions with suspected Ag^+ contamination following the completion of the growth reaction. Samples were syringe filtered to remove excess surfactant prior to analysis using a filter with pore size ≤ 0.2 µm (GE Healthcare Life Sciences Whatman GD/X 25 mm syringe filter, 0.2 µm pore, PTFE membrane; new filter and syringe used for each step). After filtering, samples were transferred to a 15 mL conical tube. The solution in the 15 mL tube was then digested with 2 mL of aqua regia (1:3 HNO_3 to HCl) made from concentrated trace metals grade acids (**caution: strong acid**). The solution was allowed to digest overnight and was then diluted to 5 mL total volume with ~2 mL ultrapure DI water and vortexed to mix. For Cu solutions, the ICP samples were prepared after exposing the grown particles to air atmosphere for at least a week, resulting in the complete dissolution of the particles. Samples were syringe filtered to remove excess surfactant prior to analysis using a filter with pore size ≤ 0.2 µm (GE Healthcare Life Sciences Whatman GD/X 25 mm syringe filter, 0.2 µm pore, PTFE membrane; new filter and syringe used for each step) and used without further dilution. All samples were prepared in triplicate. Inductively coupled plasma optical emission spectroscopy (ICP-OES) measurements were carried out using a PerkinElmer Avio 200 ICP-OES spectrometer. An Ag standard calibration curve was obtained each day that samples were run. Concentrations obtained from the raw ICP intensities and calibration curve were multiplied by 5 for the Au solutions to account for the 5-fold sample dilution factor.

II. Best Practices for ORP Sensor Measurements of Metal Nanoparticle Synthesis

Though we were able to determine appropriate protocols for meaningful measurements of Pd, Au, (Ag)Au, and Cu nanoparticle growth using ORP sensors, not all ORP sensors are equally appropriate for measuring all growth reactions. In this section, we summarize considerations for identifying combinations of synthetic protocols and ORP sensor types that have a high likelihood of allowing for a successful measurement. Many of these factors are also summarized in Table S1.

1. Considerations for Choosing an ORP Sensor Type

1.1. Possibility for Introduction of Contaminants During ORP Measurement

Leaching of Ag^+ ions from the ORP sensor's RE can poison Au and Cu growth solutions. This Ag^+ leaching can be attenuated sufficiently for accurate measurement of Au or (Ag)Au nanoparticle growth—but not completely eliminated—through the use of a DJ ORP sensor, and it is accelerated at increased temperatures, limiting the use of ORP sensor measurements in growth systems that are strongly susceptible to Ag^+ contamination.

1.2. Working Electrode Material and Area

Plating of metal ions from the growth solution onto the ORP sensor's metal WE is possible under open circuit conditions. Differences in analyte-to-WE electron transfer (ET) kinetics can change the anodic and cathodic partial currents at the WE and, therefore, the measured OCP. We particularly observed these changes for Pt WEs immersed in growth solutions containing Au ions, though it is predicted for both Au ions[14,15] and ruthenium (Ru) ions.[16–20] Complete plating of Au onto the metal WE and a commensurate upshift in the OCP are avoidable at short timescales by maximizing the SA of the WE. A best practice approach is to use the same WE material in all measurement-to-measurement comparisons, and to use a WE with a large exposed SA for measurements where spontaneous plating is expected. When comparing ORP sensor

measurements to potentiostat OCP measurements taken with a polished planar WE, it is important to note that an oxide layer on the ORP sensor's metal WE can decrease its responsiveness to changes in local chemistry and/or at fast timescales.

1.3. Working Temperature Range of the ORP Sensor

For the measurement of colloidal growth solutions at elevated reaction temperatures, selection of an ORP sensor with an expanded working temperature range facilitates improved reliability of OCP measurements and the long-term durability of the ORP sensor. While we were able to measure the OCP of Cu nanoparticle synthesis at 100 °C using the SJ epoxy ORP sensor outside of its official working temperature range, we observed differences in OCP traces taken using the SJ epoxy ORP compared to the DJ glass ORP and potentiostat OCP after multiple uses.

2. Considerations for Interpretation of ORP Sensor Measurements

While choosing the right ORP sensor is important to allow for accurate OCP measurements of colloidal growth reactions without influencing shape development, the design and materials of the ORP sensor can also influence features of the OCP measurement itself. Specific influences from different types of ORP sensors need to be taken into account when interpreting the resulting OCP traces or comparing them to benchmark measurements taken using other sensor types and/or potentiostat OCP.

2.1. Influence of the ORP Sensor on the Reaction Temperature

At reaction temperatures >40 °C, such as those used for Cu nanocube syntheses, we observe that the varied size and geometry of different commercial ORP sensors and/or electrodes used in potentiostat OCP measurements may differently influence heating gradients and/or maximum reaction temperature. Changes to the reaction temperature can result in significant differences (> 20 mV) in the OCP traces of otherwise identical colloidal growth reactions. A best practice for

OCP measurements at elevated temperatures would be to measure and report the temperature profile of the solution during the measurement, allowing researchers who are utilizing benchmark OCP measurements to troubleshoot reproducibility issues to exclude temperature effects as the source of the difference between OCP traces.

2.2. ORP Sensor Sensitivity and Measurement of Diffusion Effects

ORP sensor measurements taken with all of the sensor types used in this work show decreased responsiveness at early reaction timepoints, as compared to potentiostat OCP measurements, as we particularly noted for measurements of the Pd THH synthesis (Figure 3). This is likely due to formation of an oxide layer on the ORP sensor's Pt WE, which decreases the responsiveness of the WE to local chemical changes and/or changes at fast timescales, and is typically eliminated in potentiostat OCP measurements by mechanical polishing of planar WEs. ORP sensor measurements, therefore, should not be used to *rule out* effects of reagent diffusion, mixing, or other phenomena related to seeding and early growth, and for capturing fast ($\leq$ 5 minutes to completion) particle synthesis, as can be done more conclusively using a potentiostat OCP measurement.

III. NMR Data for Characterization of CTAC

All spectra were referenced with respect to water exchange peak at 4.79 ppm during data processing. Spectra were processed using MestReNova version 14.3.3-33362.

As-Received TCI CTAC:

^{1}H NMR (600 MHz, D_2O) δ 3.42 (t, J = 8.6 Hz, 2H), 3.18 (s, 9H), 1.79 (h, *J* = 6.8 Hz, 2H), 1.42 – 1.37 (m, 2H), 1.33 (s, 24H), 0.94 (t, *J* = 6.4 Hz, 3H).

Recrystallized, Oven-Dried TCI CTAC:

^{1}H NMR (600 MHz, D_2O) δ 3.42 – 3.36 (m, 1H), 3.19 (s, 9H), 1.80 (dq, *J* = 14.5, 7.2 Hz, 2H), 1.40 (d, *J* = 5.6 Hz, 2H), 1.33 (s, 24H), 0.91 (t, *J* = 6.9 Hz, 1H).

As-Received Sigma CTAC:

^{1}H NMR (600 MHz, 90% H_2O + 10% D_2O) δ 3.37 (t, *J* = 8.1 Hz, 2H), 3.17 (s, 9H), 2.90 (s, 0.2H), 1.79 (m, 2H), 1.39 (s, 2H), 1.32 (s, 24H), 0.93 – 0.88 (m, 3H).

Lyophilized, Oven-Dried Sigma CTAC:

^{1}H NMR (600 MHz, D_2O) δ 3.42 – 3.37 (m, 2H), 3.19 (s, 9H), 1.80 (dd, *J* = 11.1, 6.0 Hz, 2H), 1.45 – 1.37 (m, 2H), 1.33 (s, 24H), 0.91 (t, *J* = 6.8 Hz, 3H).

Lyophilized, Recrystallized, Oven-Dried Sigma CTAC:

^{1}H NMR (600 MHz, D_2O) δ 3.42 – 3.36 (m, 2H), 3.18 (s, 9H), 1.84 – 1.76 (m, 2H), 1.40 (d, *J* = 5.4 Hz, 2H), 1.32 (s, 24H), 0.91 (t, *J* = 6.9 Hz, 3H).

IV. Supplementary Figures and Tables

Table S1. Overview of Characteristics and Proposed Applications of ORP Sensor Types Used in this Publication, with a Comparison to the Potentiostat OCP Method.

	"SJ epoxy ORP"	"DJ glass ORP"	Potentiostat OCP
**WE material**	Pt	Pt	Variable (GC, Pt, Au, other conductive materials)
**WE geometry**	band	band	Variable<br>Disk, wire, mesh
**WE SA**	~ 6 mm^2	~97 mm^2	Variable
**Reference electrode**	Ag/AgCl	Ag/AgCl	Variable (Ag/AgCl, MSE, RHE, SCE[a])
**# of junctions**	1	2	Variable (0 to 2)
**Shaft material**	Epoxy	Glass	N/A
**Temperature range**	0-60 °C	-5 °C-100 °C	Variable, dependent on RE
**Best used in…**	Reactions tolerant to Ag^+ contamination (<1 µM).<br>Au- and Rh-free growth solutions<br>Reaction time of >5 min	Reactions tolerant to low levels of Ag^+ contamination (<230 nM).<br>Au- and Rh- containing growth solutions with 5-30 min reaction time<br>Other growth solutions with reaction time of >5 min	All reactions, depending on WE and RE pair<br>Pd, Au, Cu, Rh, Ag<br>Fast reactions<br>Au- and Rh-containing solutions with reaction times of >30 min
**Best used for …**	Benchmarking & troubleshooting	Benchmarking & troubleshooting	Benchmarking & troubleshooting<br>Mechanistic investigations
**Manufacturer**	Vernier	Cole-Parmer	Variable
**Full name**	ORP Sensor	Cole-Parmer ORP electrode, double-junction, platinum band, 10-ft cable	N/A
**Catalog #**	ORP-BTA	EW-27006-21	N/A

[a] Saturated calomel electrode (SCE)

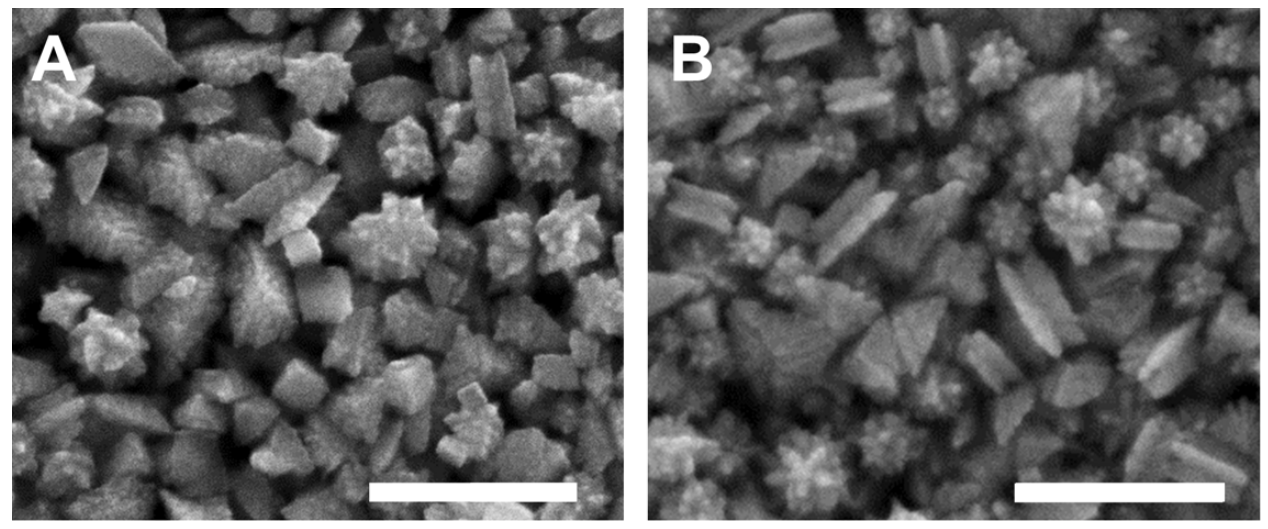

Figure S1. (A-B) Additional representative SEM images of corrugated Pd nanoparticles synthesized in Sigma CTAC, measured during synthesis with a SJ epoxy ORP sensor. Scale bars: 500 nm.

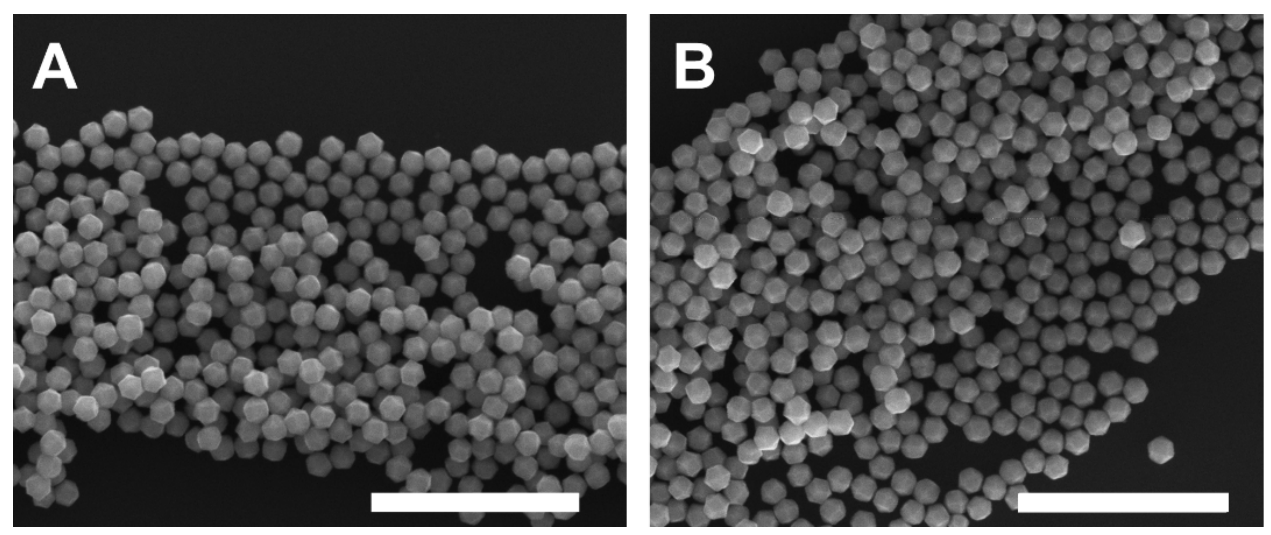

Figure S2. (A-B) SEM images of Pd THH nanoparticles from (A) an unmeasured growth solution and (B) a growth solution measured with a SJ epoxy ORP sensor (Pt band WE vs. Ag/AgCl RE). Scale bars: 2 μm.

Table S2. Size and Shape Distributions of Pd THH Nanoparticles.

Measurement Type	# of Particles Measured	Size (nm) [a]	% THH	% Other Shapes
Unmeasured	907	138 ± 11	99	1
SJ Epoxy ORP	1126	137 ± 13	98	2
DJ Glass ORP	873	142 ± 8	99	1

[a] THH nanoparticles measured diagonally.

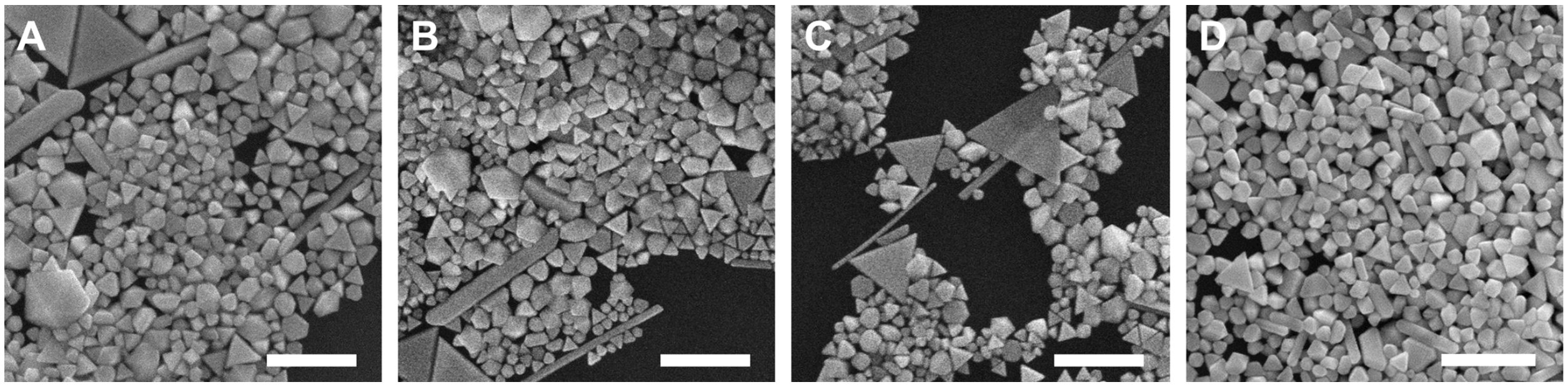

Figure S3. (A-D) SEM images of monometallic Au nanoparticles synthesized in Sigma CTAC from (A) an unmeasured growth solution, (B) a growth solution measured during synthesis with potentiostat OCP using a GC WE vs. a Hg/Hg_2SO_4 RE, (C) a growth solution measured with a SJ epoxy ORP sensor (Pt band WE vs. Ag/AgCl RE), and (D) a growth solution measured with potentiostat OCP using a GC WE vs. a Ag/AgCl RE. Scale bars: 500 nm.

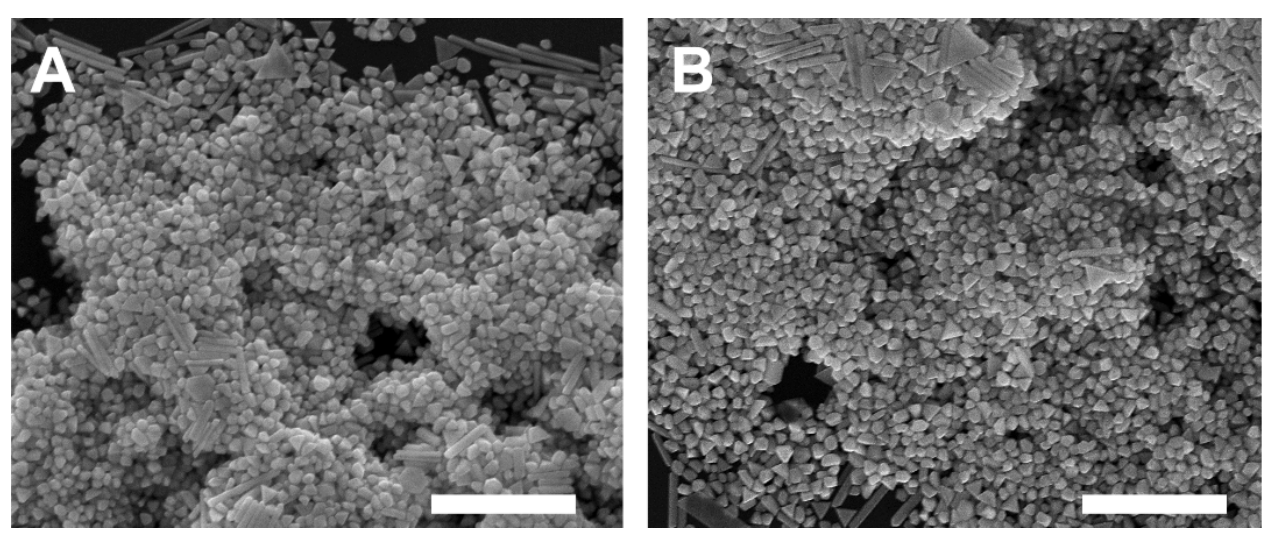

Figure S4. (A-B) Low magnification SEM images of monometallic Au nanoparticles from (A) an unmeasured growth solution and (B) a growth solution measured with a SJ epoxy ORP sensor (Pt band WE vs. Ag/AgCl RE). Scale bars: 1 μm.

Table S3. Shape Distributions of Monometallic Au Nanoparticles.

**Particle Shape**	**Unmeasured** [a]	**SJ Epoxy ORP** [b]	**DJ Glass ORP** [c]
Octahedra	48.4 %	52.0%	47.3%
Truncated Bitetrahedra	30.3%	31.4%	32.5%
Decahedra	3.9%	3.4%	2.7%
Pentatwinned Rod	12.9%	11.3%	12.2%
Triangular/Hexagonal Prism	1.3%	1.4%	2.6%
Unknown	3.2%	0.5%	2.7%

[a] n = 1240 nanoparticles counted.
[b] n = 989 nanoparticles counted.
[c] n = 953 nanoparticles counted.

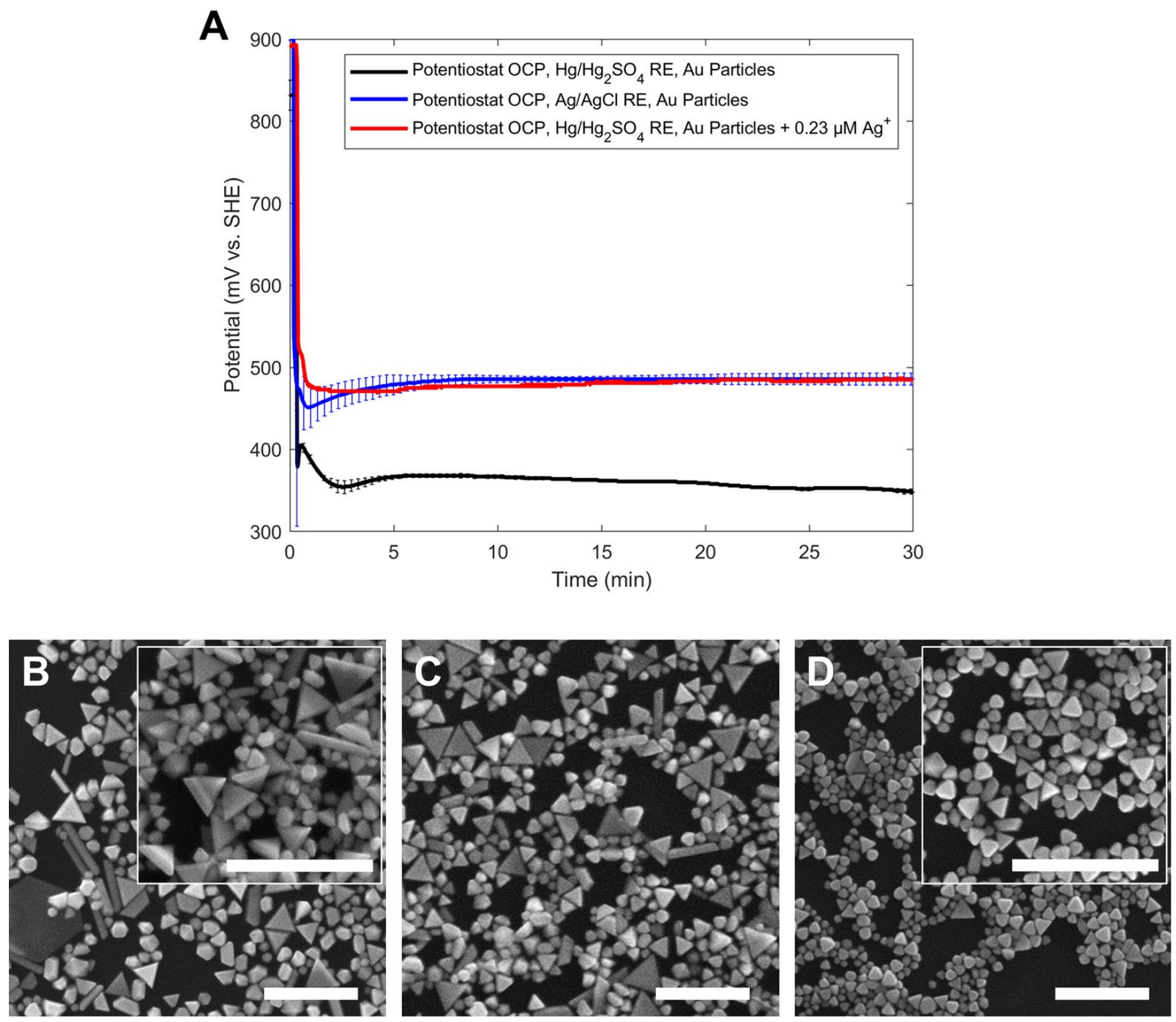

Figure S5. (A) Comparison between potentiostat OCP measurements of monometallic nanoparticle growth taken using a GC WE vs. a Hg/Hg_2SO_4 RE (black), using a GC WE vs. a Ag/AgCl RE (blue), and of an Au nanoparticle growth solution with 0.23 μM added Ag^+ taken using a GC WE vs. a Hg/Hg_2SO_4 RE (red). Error bars represent the standard deviation of three measurements. (B-D) SEM images of Au nanoparticle products with (B) 0.0092 μM (9.2 nM) added Ag^+, (C) 0.23 μM added Ag^+, and (D) 0.46 μM added Ag^+. Scale bars: 500 nm. Black trace in (A) adapted with permission from ref. 8. Available under a Creative Commons CC-BY 4.0 license. Copyright 2026 Halford, Sublett, and Personick.

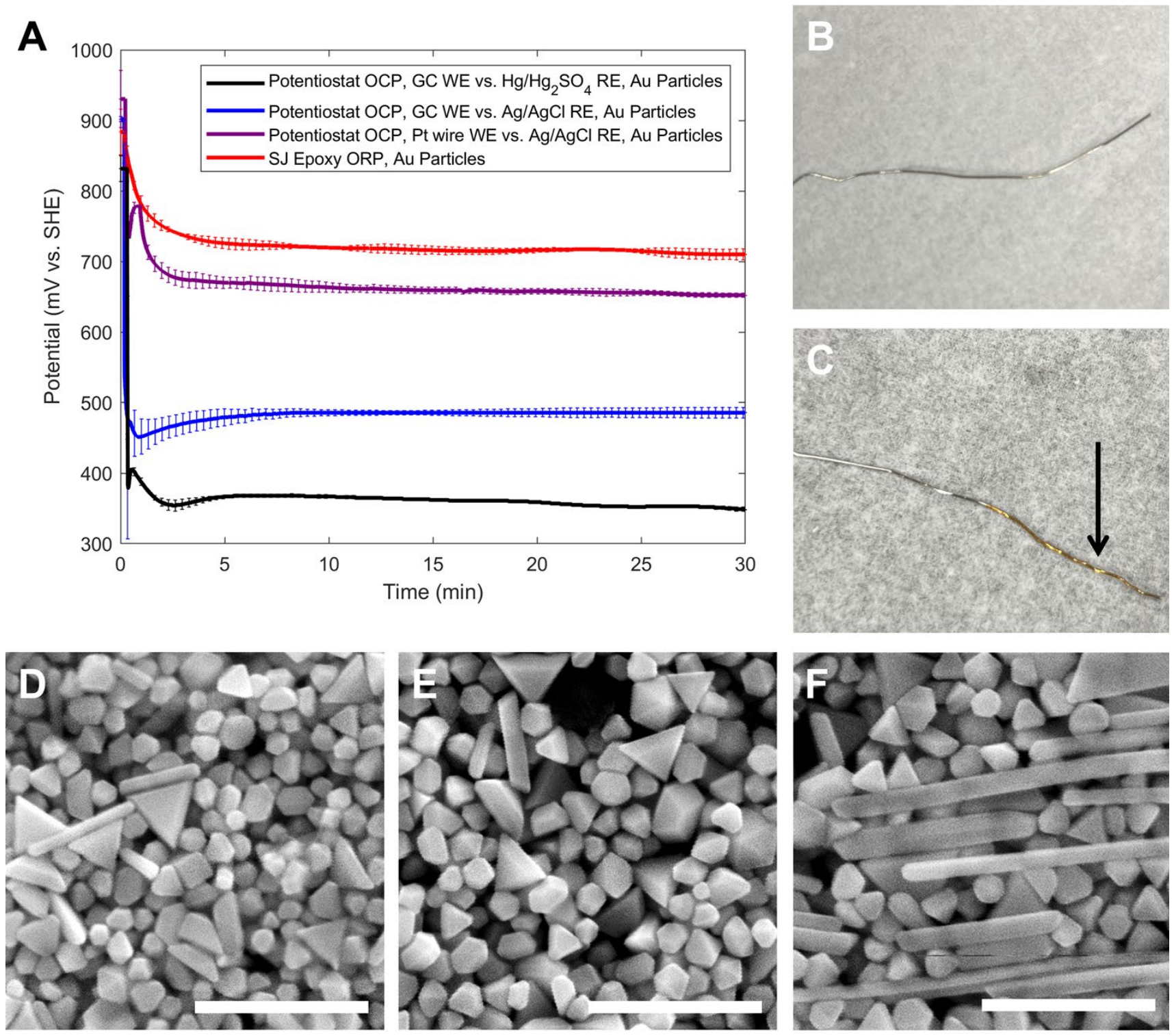

Figure S6. (A) Comparison between OCP measurements of monometallic Au nanoparticle growth taken with potentiostat OCP using a GC WE vs. a Hg/Hg_2SO_4 RE (black), potentiostat OCP using a GC WE vs. a Ag/AgCl RE (blue), potentiostat OCP measurements taken with a Pt wire WE vs. a Ag/AgCl RE (purple), and SJ Epoxy ORP sensor (Pt band WE vs. Ag/AgCl RE; red). Error bars represent the standard deviation of three measurements. (B-C) Photographs of Pt wire WE (B) before and (C) after use in a 30-minute measurement of Au nanoparticle growth. (D-F) SEM images of Au nanoparticle products measured via potentiostat OCP using a Pt wire WE vs. a Ag/AgCl RE. Scale bars: 500 nm. Black trace in (A) adapted with permission from ref. 8. Available under a Creative Commons CC-BY 4.0 license. Copyright 2026 Halford, Sublett, and Personick.

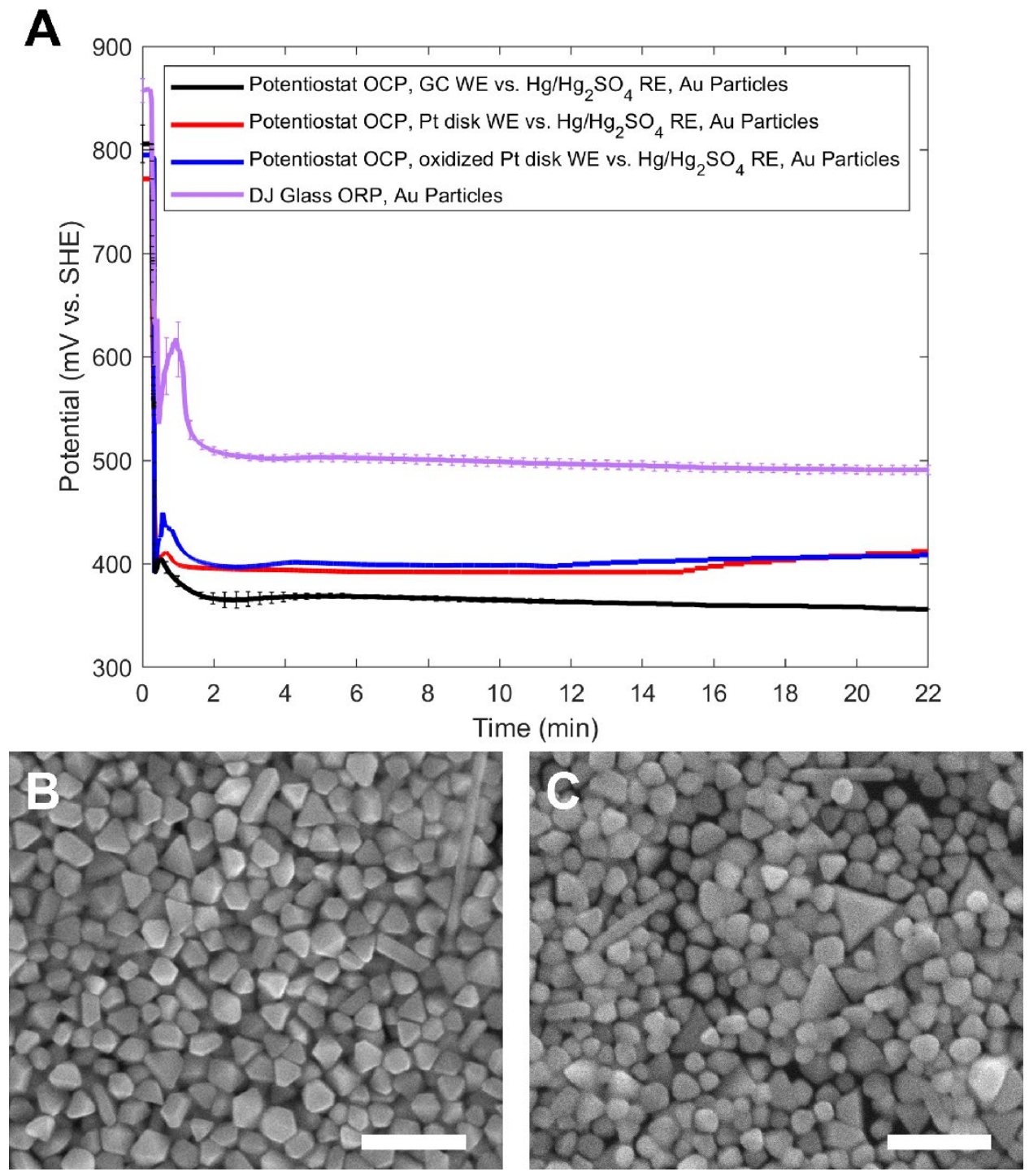

Figure S7. (A) Comparison between potentiostat OCP measurements using a GC WE vs. a Hg/Hg_2SO_4 RE (black), a Pt disk WE vs. a Hg/Hg_2SO_4 RE (red), an oxidized Pt disk WE vs. a Hg/Hg_2SO_4 RE (blue), and a DJ glass ORP measurement (Pt band WE vs. Ag/AgCl RE; purple) of monometallic Au nanoparticle growth. Error bars (purple and black traces) represent the standard deviation of three measurements. (B-C) SEM images of Au nanoparticles, measured during synthesis with (B) potentiostat OCP using a Pt disk WE vs. a Hg/Hg_2SO_4 RE (red trace) and (C) potentiostat OCP using an oxidized Pt disk WE vs. a Hg/Hg_2SO_4 RE (blue trace). Scale bars: 500 nm. Black trace in (A) adapted with permission from ref. 8. Available under a Creative Commons CC-BY 4.0 license. Copyright 2026 Halford, Sublett, and Personick.

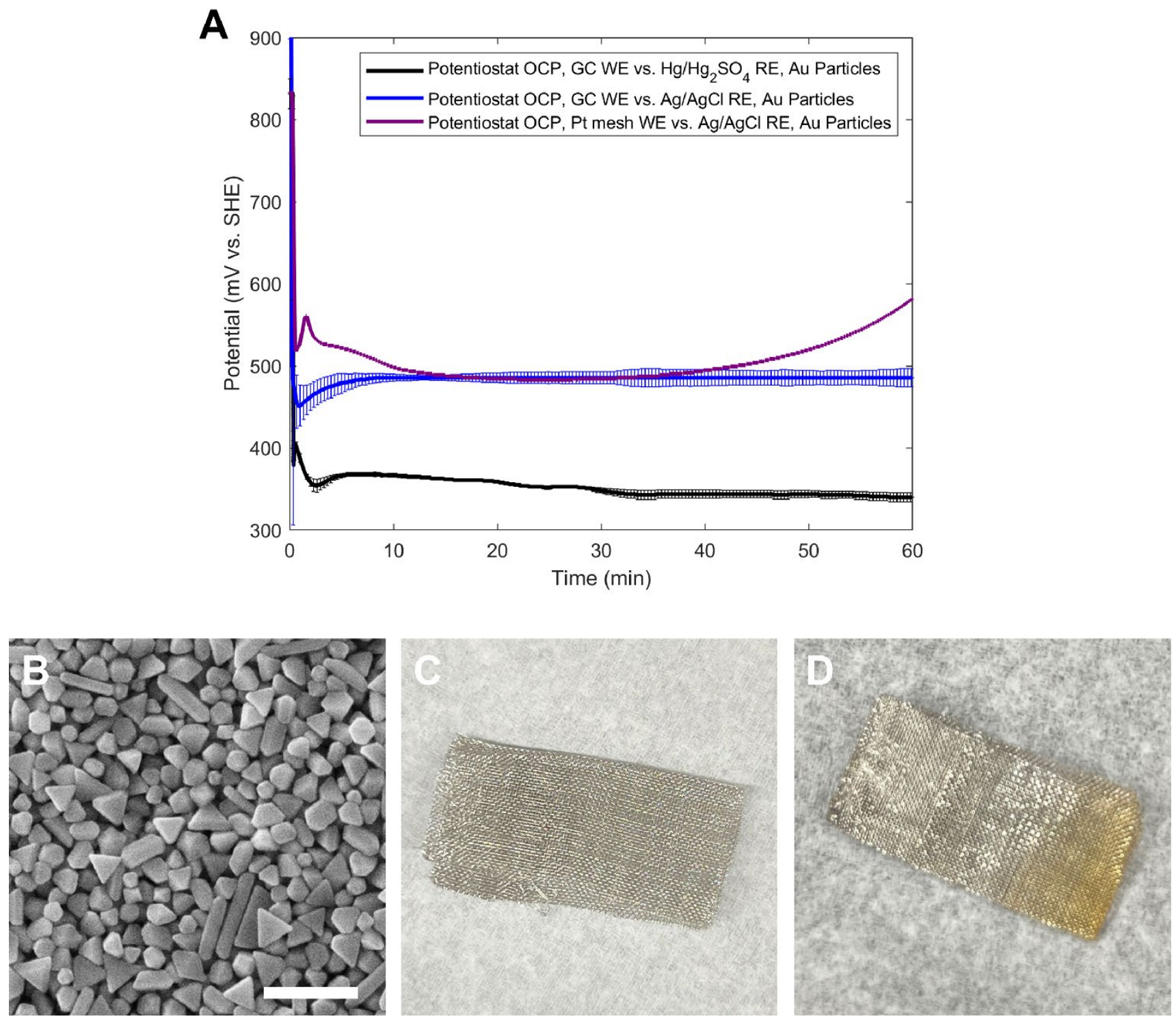

Figure S8. (A) Comparison between OCP measurements of monometallic Au nanoparticle growth taken with potentiostat OCP using a GC WE vs. a Hg/Hg_2SO_4 RE (black), potentiostat OCP using a GC WE vs. a Ag/AgCl RE (blue), and potentiostat OCP using a Pt mesh WE vs. a Ag/AgCl RE (purple). Error bars represent the standard deviation of three measurements. (B) SEM image of Au nanoparticle products measured with potentiostat OCP using a Pt mesh WE vs. a Ag/AgCl RE and Pt mesh WE. (C-D) Photograph of Pt mesh WE taken after (C) 26 minutes and (D) 60 minutes of immersion in Au nanoparticle growth solution during OCP measurement. Scale bars: 500 nm. Black trace in (A) adapted with permission from ref. 8. Available under a Creative Commons CC-BY 4.0 license. Copyright 2026 Halford, Sublett, and Personick.

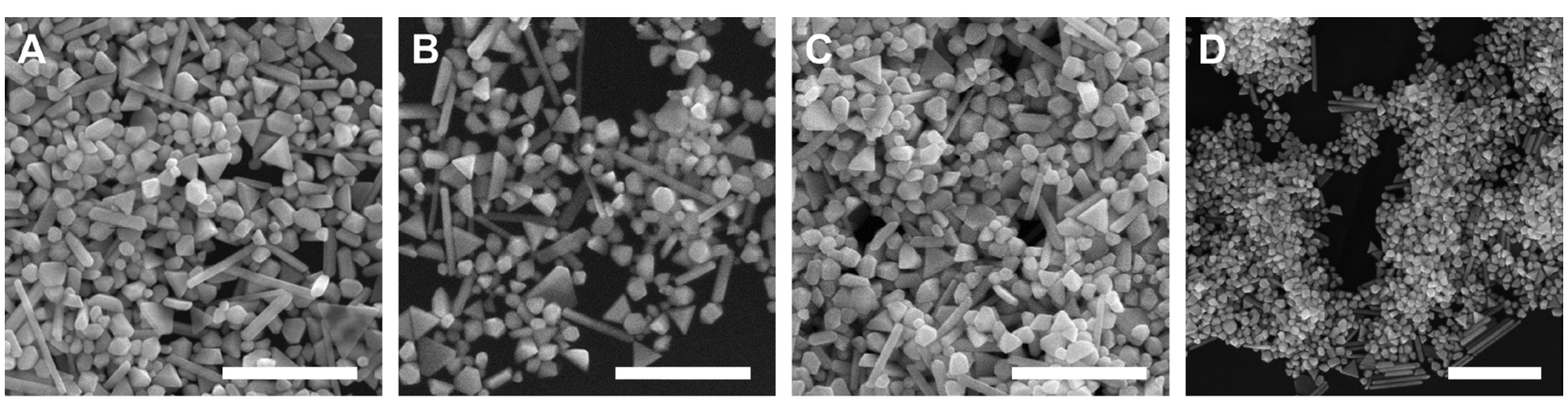

Figure S9. (A-D) SEM images of monometallic Au nanoparticles from growth solutions measured with a DJ glass ORP sensor (Pt band WE vs. Ag/AgCl RE). Scale bars: 500 nm (A-C) and 1 µm (D).

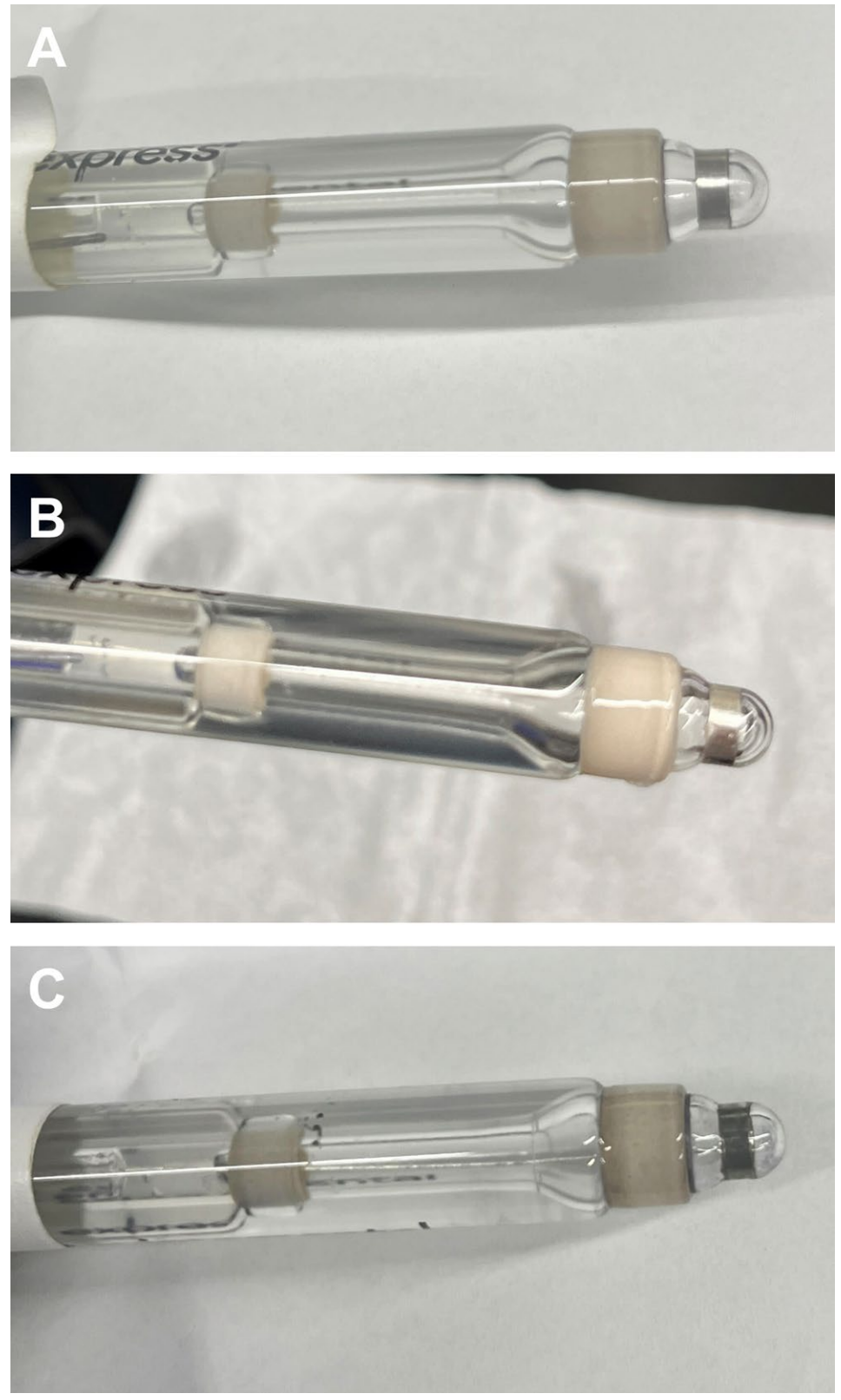

Figure S10. (A-C) Photographs of DJ glass ORP sensor (A) before use; (B) after a 30-miute measurement of monometallic Au nanoparticle growth, showing minimal Au deposition on the Pt band WE; and (C) after a 60-minute measurement of monometallic Au nanoparticle growth, showing more significant Au deposition on the Pt band WE.

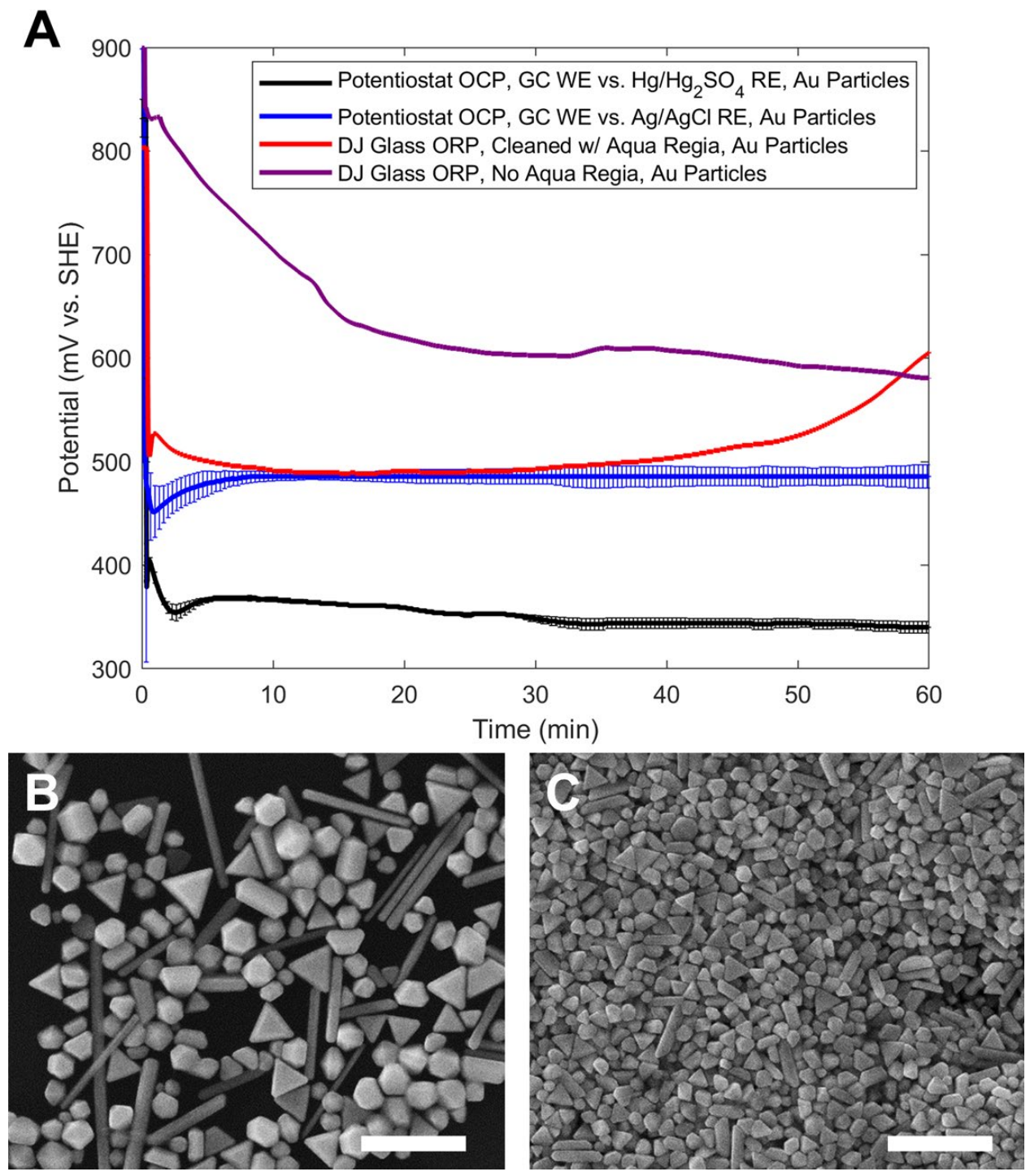

Figure S11. (A) Comparison between 60 minute OCP measurements of monometallic Au particle growth: potentiostat OCP measurement using a GC WE vs. a Hg/Hg_2SO_4 RE (black), potentiostat OCP measurement using a GC WE vs. a Ag/AgCl RE (blue), DJ glass ORP sensor measurement (Pt band WE vs. Ag/AgCl RE; red), and DJ glass ORP sensor without prior aqua regia cleaning (Pt band WE vs. Ag/AgCl RE; previously used for 60 minute measurement of monometallic Au nanoparticle growth; purple). The clean DJ glass ORP sensor measurement begins to deviate from the potentiostat OCP at ~30 minutes due to plating of Au on the Pt band WE. Error bars represent the standard deviation of three measurements. (B-C) SEM images of the monometallic Au nanoparticle products from growth solutions measured for 60 minutes with a DJ glass ORP sensor that was (B) cleaned or (C) not cleaned with aqua regia between measurements. Scale bars: 500 nm. Black trace in (A) adapted with permission from ref. 8. Available under a Creative Commons CC-BY 4.0 license. Copyright 2026 Halford, Sublett, and Personick.

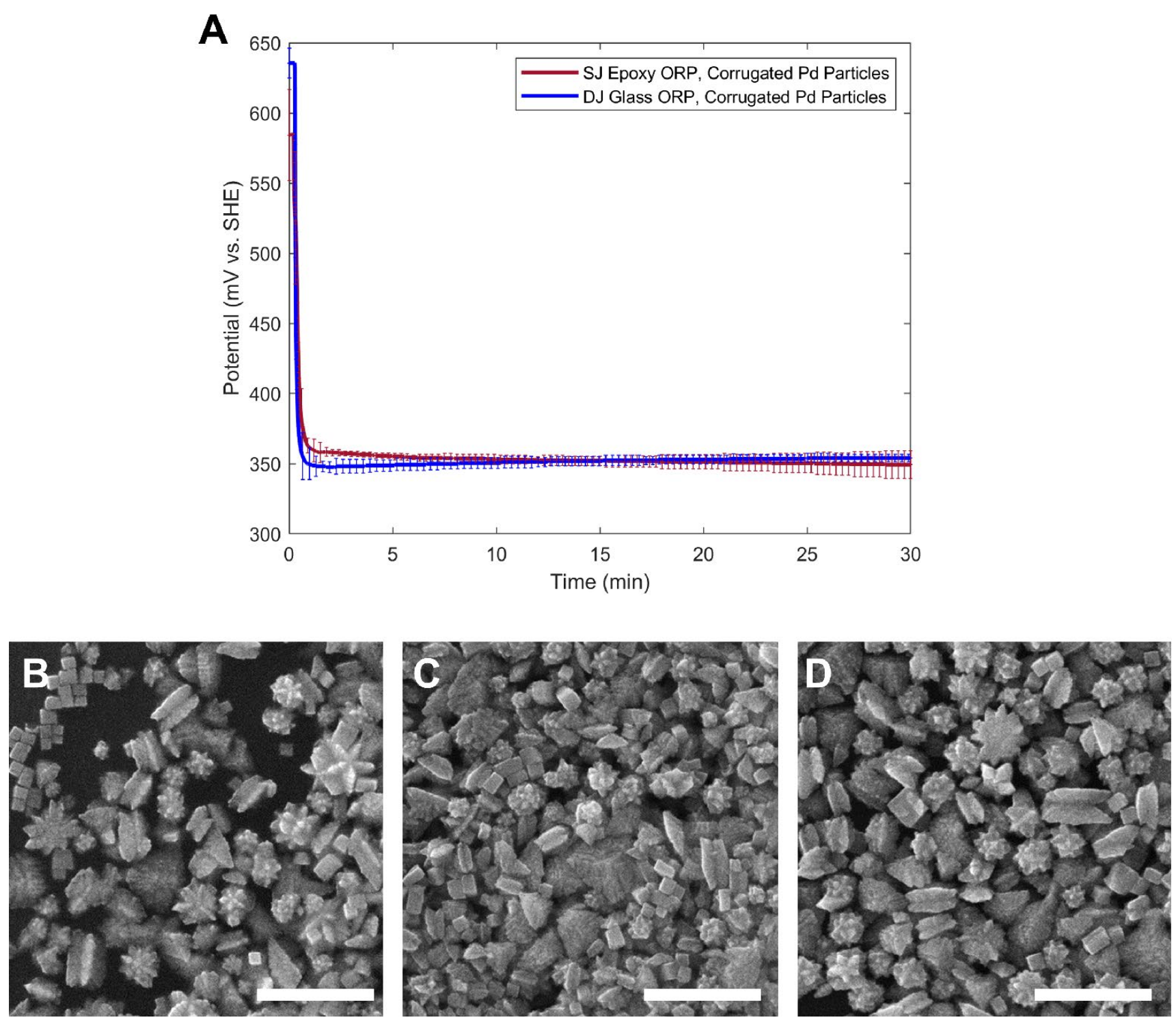

Figure S12. (A) Comparison between SJ epoxy ORP measurement (Pt band WE vs. Ag/AgCl RE; red) and DJ glass ORP measurement (Pt band WE vs. Ag/AgCl RE; blue) of Pd corrugated particle growth. Error bars represent the standard deviation of three measurements. (B-D) SEM images of the Pd corrugated particle products from each growth solution measured with a DJ glass ORP sensor. Scale bars: 500 nm.

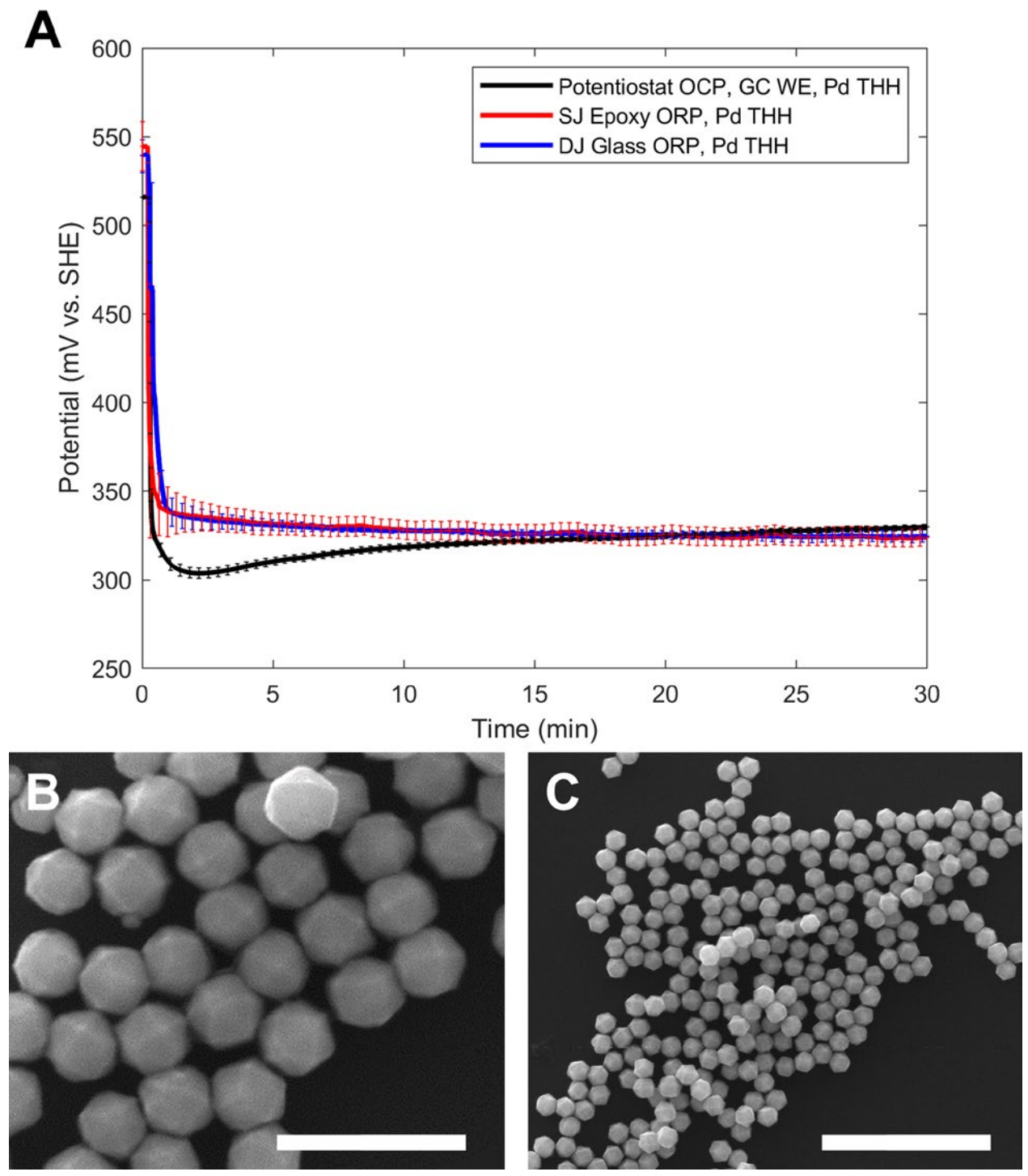

Figure S13. (A) Comparison between potentiostat OCP measurements using a GC WE vs. a Hg/Hg_2SO_4 RE (black), SJ epoxy ORP measurements (Pt band WE vs. Ag/AgCl RE; red), and DJ glass ORP measurements (Pt band WE vs. Ag/AgCl RE; blue) of the first thirty minutes of Pd THH growth. Error bars represent the standard deviation of three measurements. (B-C) SEM images of Pd THH, measured during synthesis with a DJ glass ORP sensor. Scale bars: 500 nm (B) and 2 µm (C). Black trace in (A) adapted with permission from ref. 8. Available under a Creative Commons CC-BY 4.0 license. Copyright 2026 Halford, Sublett, and Personick.

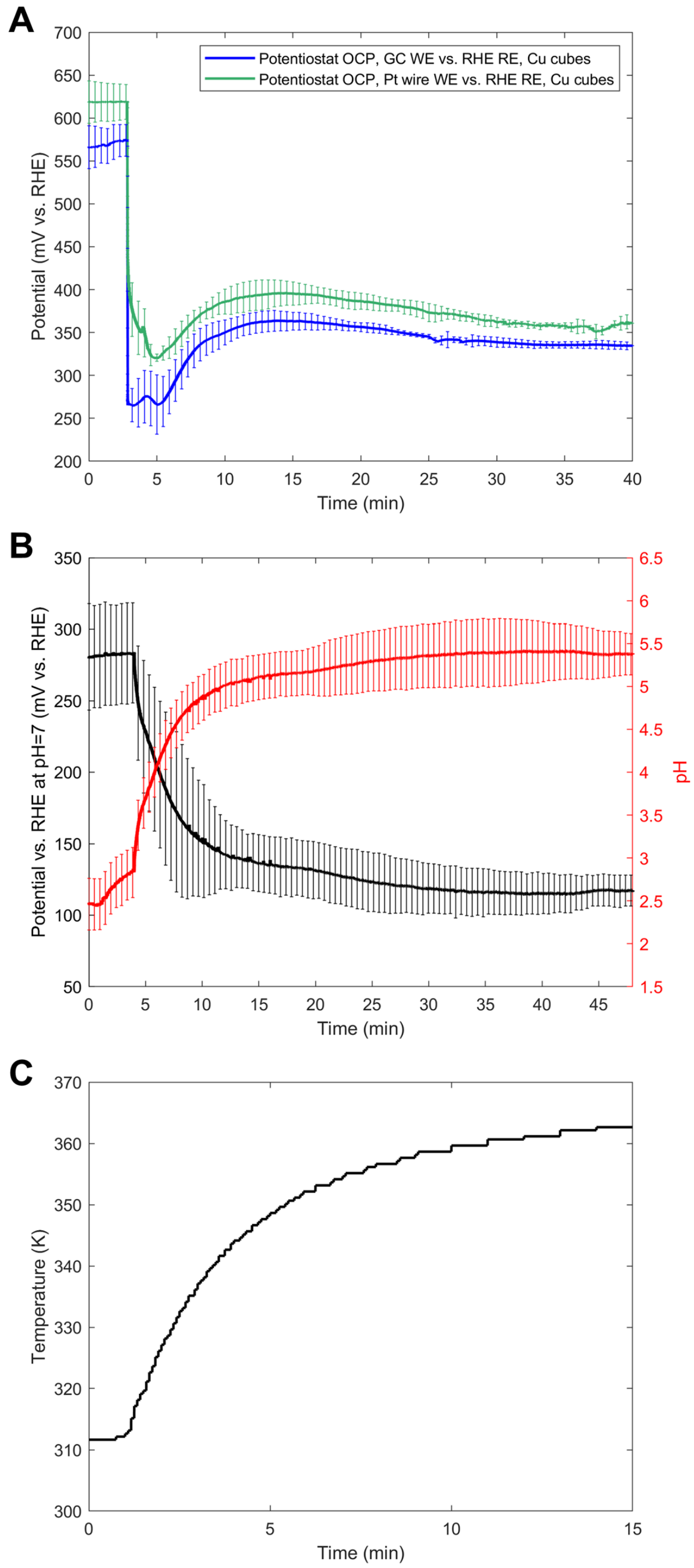

Figure S14. (A) Potentiostat OCP measurements of CTAC/NaAsc Cu nanocube synthesis using a GC WE vs. RHE RE (blue) and a Pt wire WE vs. RHE RE (green). (B) Potential of the pH probe (red) and pH profile (black) over time of Cu nanocube synthesis. Error bars represent the standard deviation of three measurements. (C) Temperature profile of CTAC/SA Cu nanocube synthesis. Temperature assumed constant after 15 minutes.

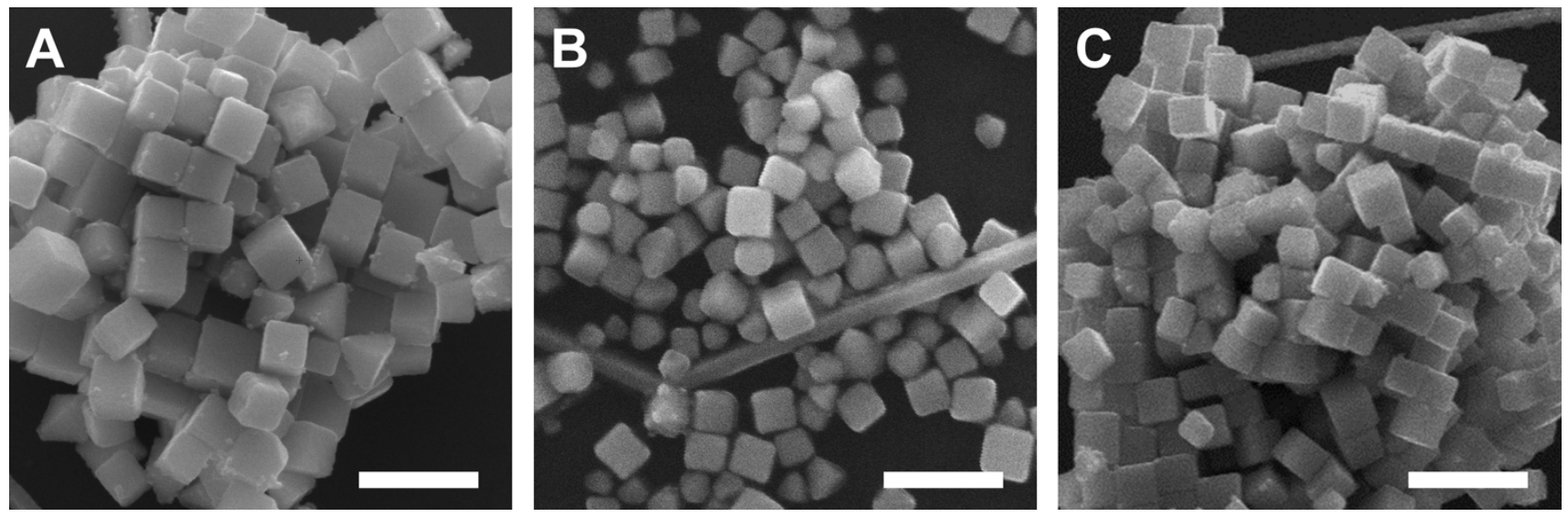

Figure S15. (A-C) SEM images of products of unmeasured CTAC/NaAsc Cu nanocube synthesis. Scale bars: 500 nm.

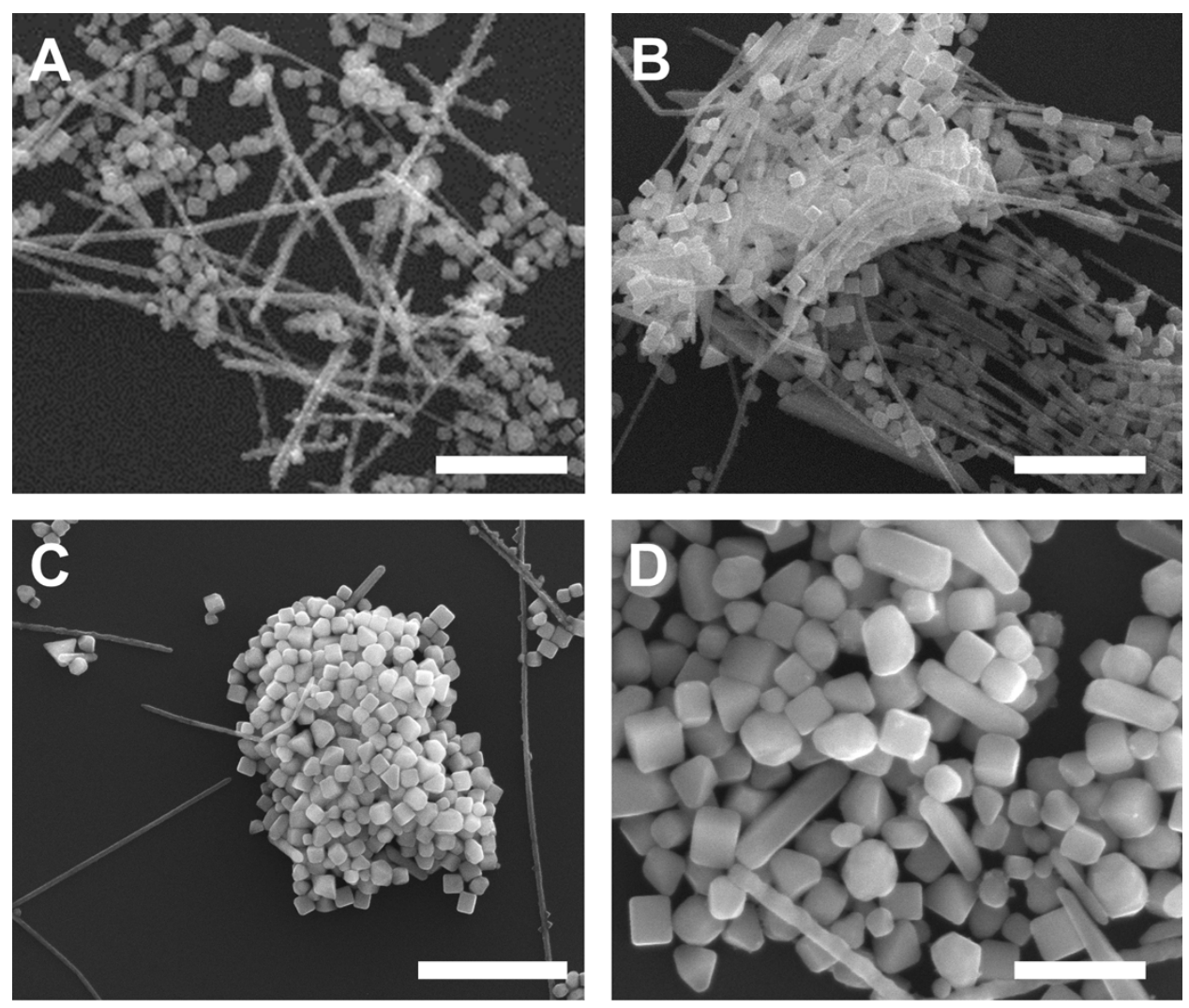

Figure S16. (A-D) SEM images of CTAC/SA Cu nanocubes with nanowire side products, measured during synthesis with potentiostat OCP using a GC WE vs. RHE RE (A, B) or Pt wire WE vs. RHE RE (C, D). Scale bars for (A-C): 1 μm. Scale bar for (D): 500 nm.

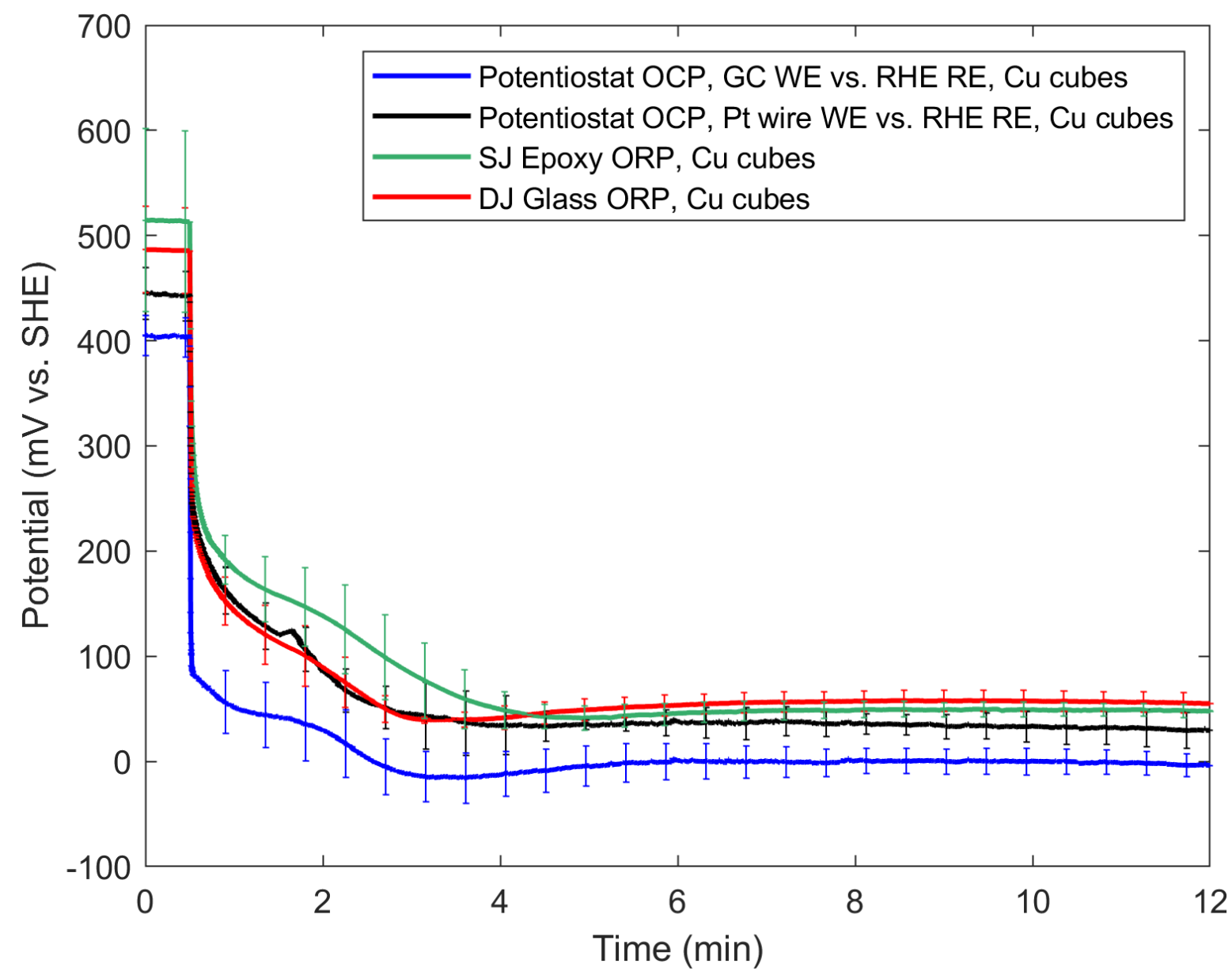

Figure S17. Zoomed-in view of the first twelve minutes of the OCP traces shown in Figure 6A. Comparison between OCP measurements of the CTAC/NaAsc Cu nanocube synthesis taken with potentiostat OCP using a GC WE vs. a RHE RE (blue), potentiostat OCP using a Pt wire WE vs. a RHE RE (black), a SJ epoxy ORP sensor (green; Pt band WE vs. a Ag/AgCl RE), and a DJ glass ORP sensor (red; Pt band WE vs. a Ag/AgCl RE). Error bars represent the standard deviation of three measurements.

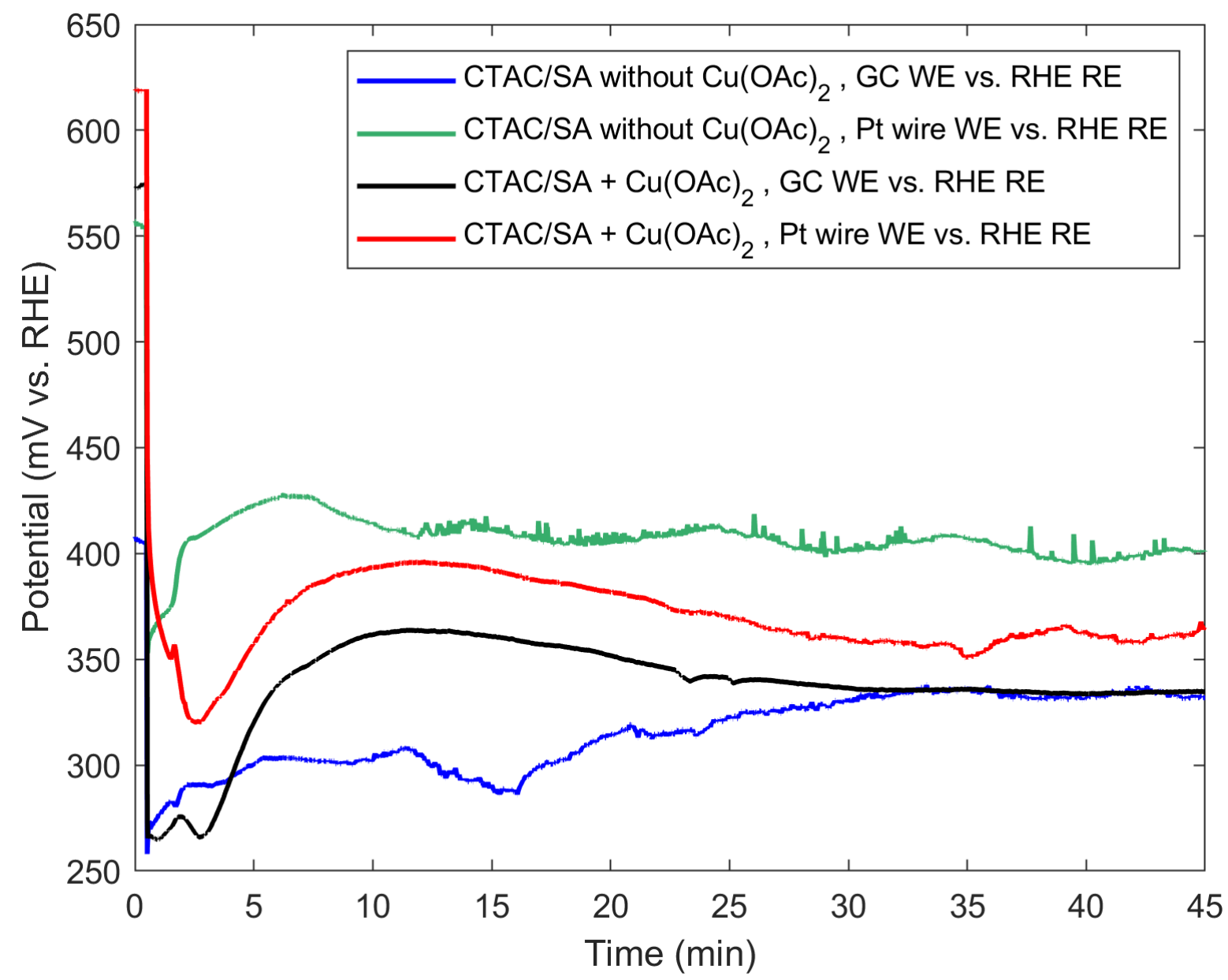

Figure S18. Potentiostat OCP measurements of CTAC/NaAsc Cu nanocube synthesis growth solutions containing (blue, green) and without (black, red) the $Cu(OAc)_2$ metal precursor, taken using a GC WE vs. RHE RE (blue, black) or a Pt wire vs. RHE RE (green, red). Growth solutions containing $Cu(OAc)_2$ are nanoparticle-forming, while solutions without $Cu(OAc)_2$ do not form metal nanoparticles. The potentiostat OCP measurements shown for the nanoparticle-forming conditions (blue, green) are the same as in Figure 6 and Figure S17, plotted vs. RHE instead of vs. SHE.

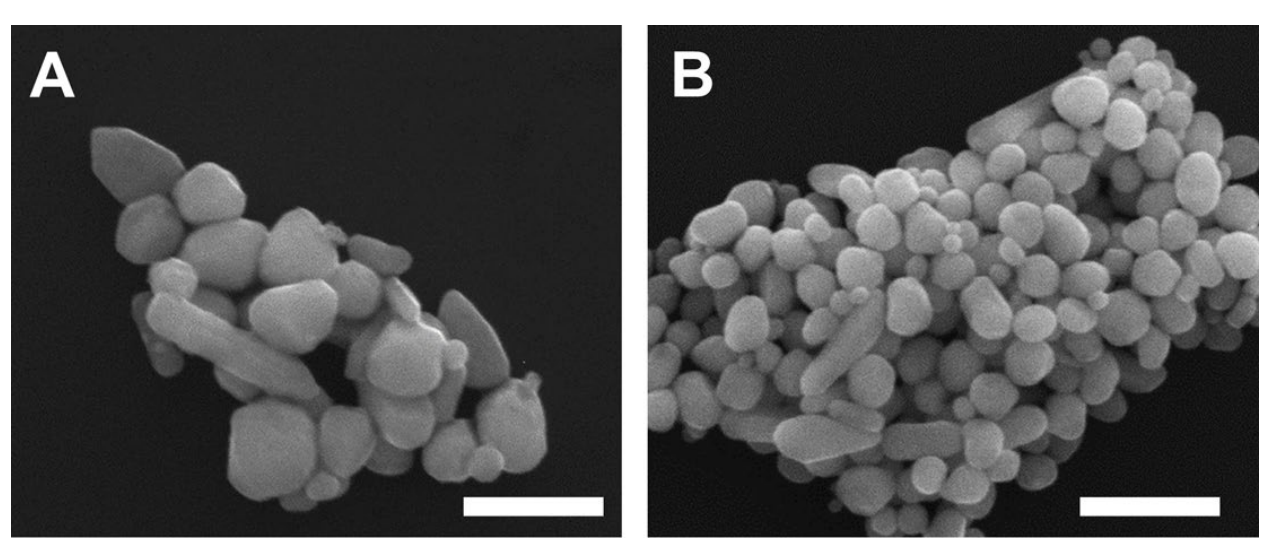

Figure S19. SEM images of Cu nanoparticles generated with (A) 500 nM $AgNO_3$ and (B) 100 nM $AgNO_3$ added to the CTAC growth solution prior to the addition of NaAsc. Scale bars: 500 nm.

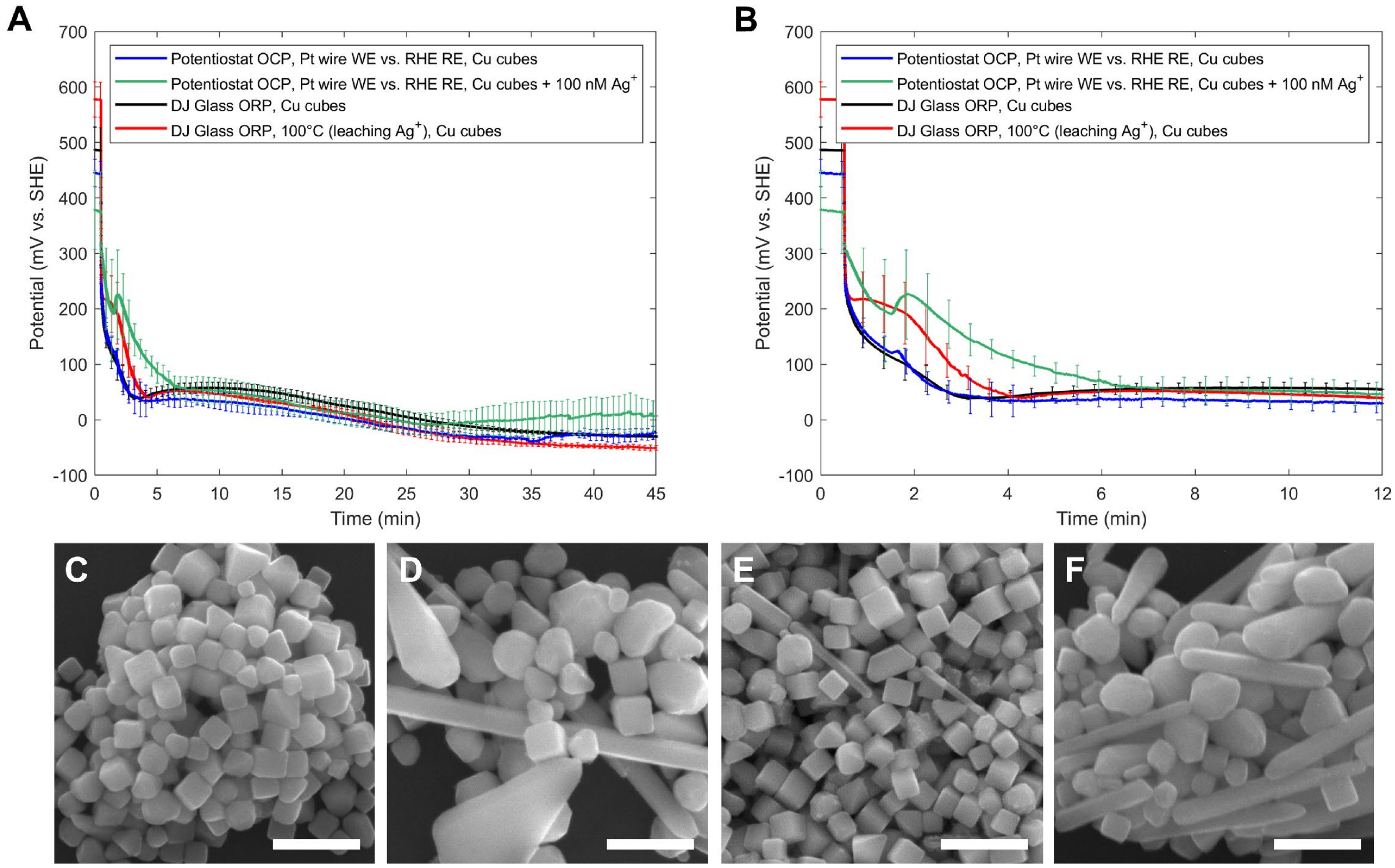

Figure S20. (A) OCP measurements of CTAC/NaAsc Cu nanocube synthesis taken with: potentiostat OCP using a Pt wire WE vs. a RHE RE at 100 °C (blue); potentiostat OCP using a Pt wire WE vs. a RHE RE at 100 °C, with 100 nM Ag^+ added to the growth solution (green); a DJ glass ORP sensor at 90 °C (Pt band WE vs. Ag/AgCl RE; black); and a well-used DJ glass ORP sensor at 100 °C (a condition that leads to increased Ag^+ leaching; Pt band WE vs. a Ag/AgCl RE; red). (B) Zoomed-in view of the first twelve minutes of the OCP traces shown in Figure S20A. Error bars represent the standard deviation of three measurements. (C-E) SEM images of Cu nanoparticles measured with (C) potentiostat OCP using a Pt wire WE vs. a RHE RE at 100 °C, (D) potentiostat OCP using a Pt wire WE vs. a RHE RE at 100 °C, with 100 nM Ag^+ added to the growth solution, (E) a DJ glass ORP sensor at 90 °C, and (F) a well-used DJ glass ORP sensor at 100 °C. Scale bars: 500 nm.

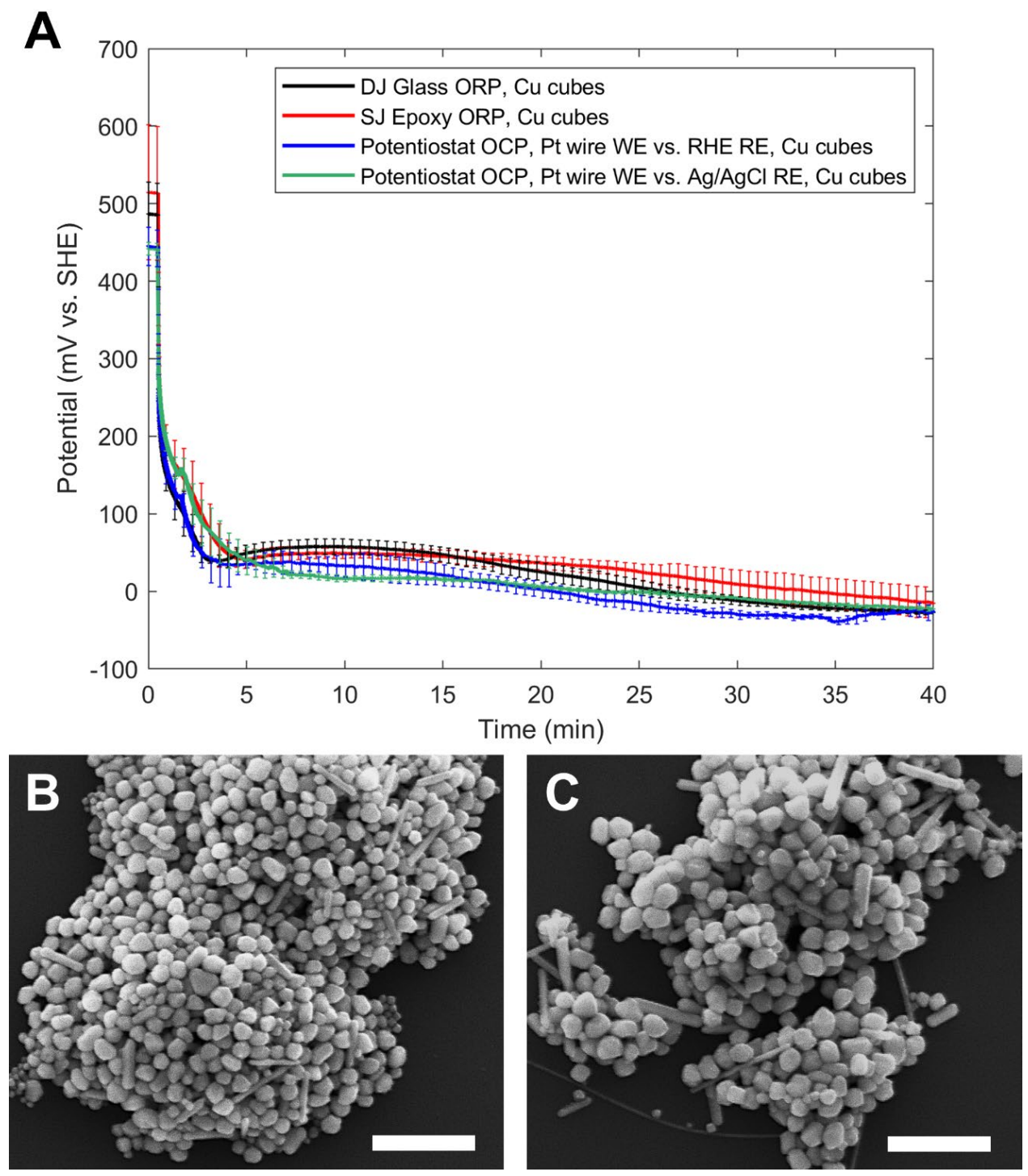

Figure S21. (A) OCP measurements of the CTAC/NaAsc Cu nanocube synthesis taken with a DJ glass ORP sensor (Pt band WE vs. Ag/AgCl RE; black), a SJ epoxy ORP sensor (Pt band WE vs. Ag/AgCl RE; red), potentiostat OCP using a Pt wire vs. a RHE RE (blue), and potentiostat OCP using a Pt wire vs. a Ag/AgCl RE (green). Error bars represent the standard deviation of three measurements. (B-C) SEM images of CTAC/NaAsc Cu nanocubes synthesized during OCP measurement with potentiostat OCP using a Pt wire WE vs. a Ag/AgCl RE. Scale bars: 1 μm.

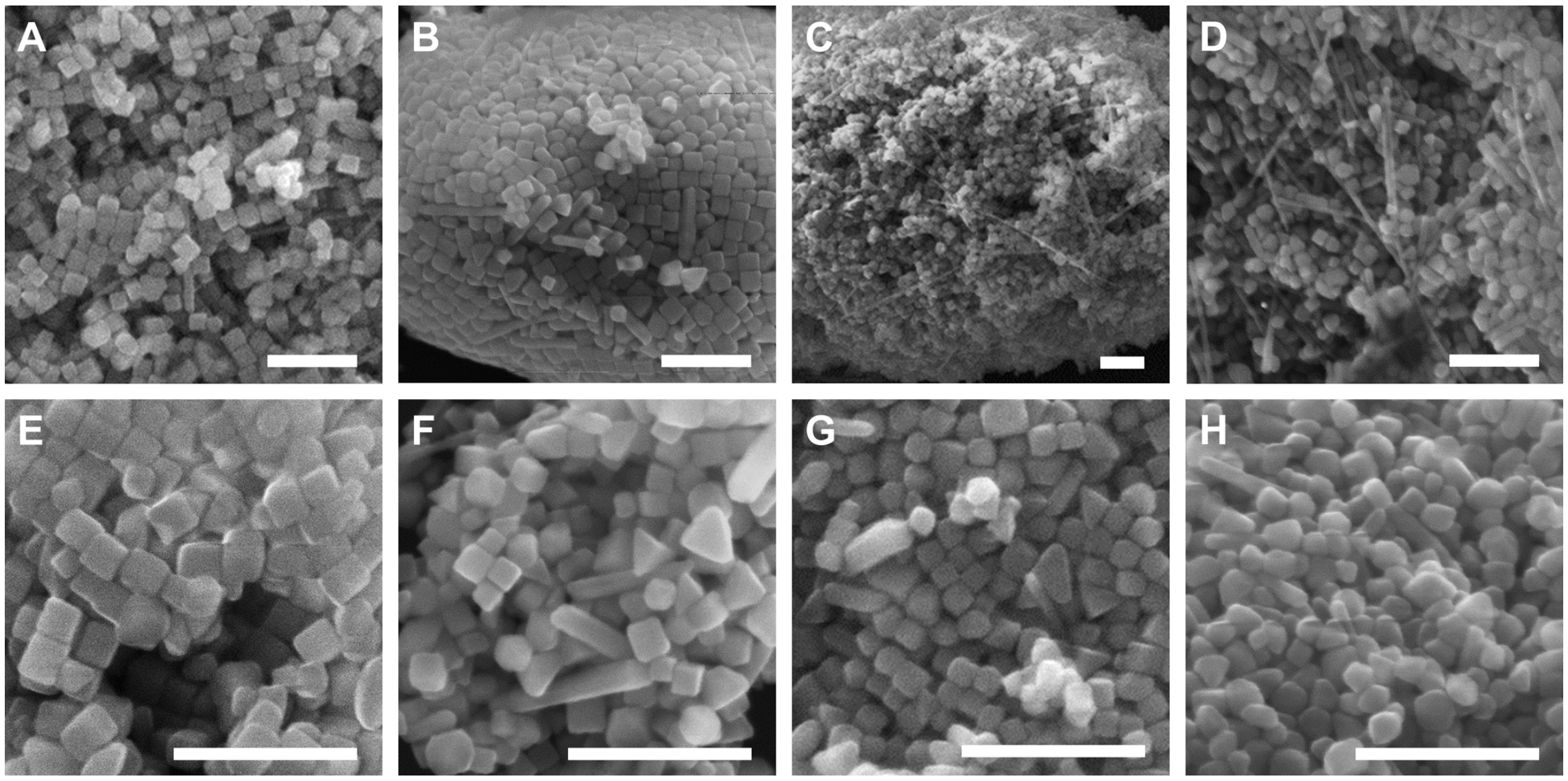

Figure S22. SEM images of HDA/AA Cu nanocube synthesis with increasing concentrations of added $AgNO_3$. (A, E) 1 µM $AgNO_3$, (B, F) 5 µM $AgNO_3$, (C, G) 10 µM $AgNO_3$, and (D, H) 50 µM $AgNO_3$. Scale bars: 500 nm.

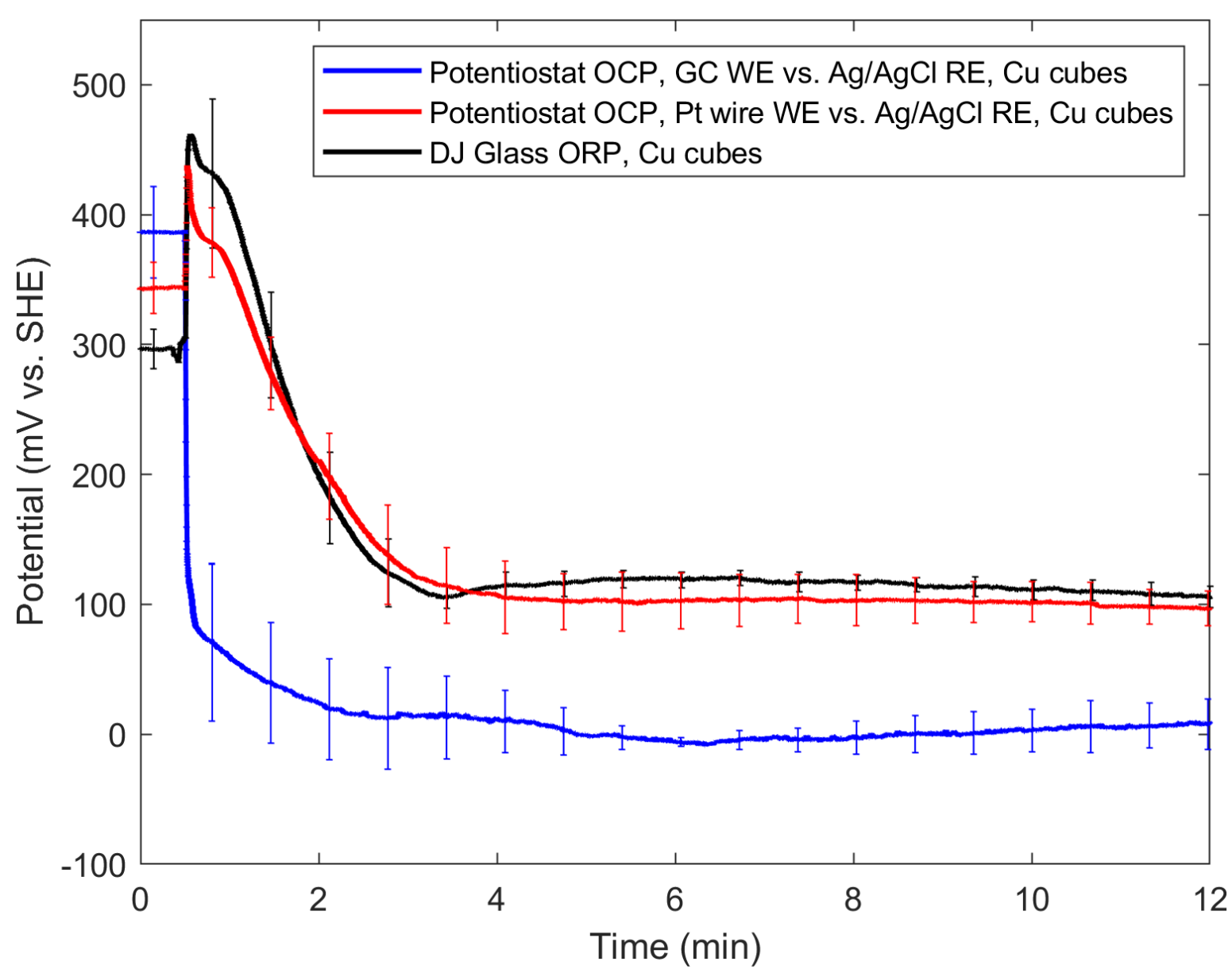

Figure S23. Zoomed-in view of the first twelve minutes of the OCP traces shown in Figure 7A. Comparison between OCP measurements of the Pd-seeded HDA/AA Cu nanocube synthesis taken with potentiostat OCP using a GC WE vs. a Ag/AgCl RE (blue), potentiostat OCP using a Pt wire WE vs. a Ag/AgCl RE (red), and a DJ glass ORP sensor (black; Pt band WE vs. Ag/AgCl RE). Error bars represent the standard deviation of three measurements.

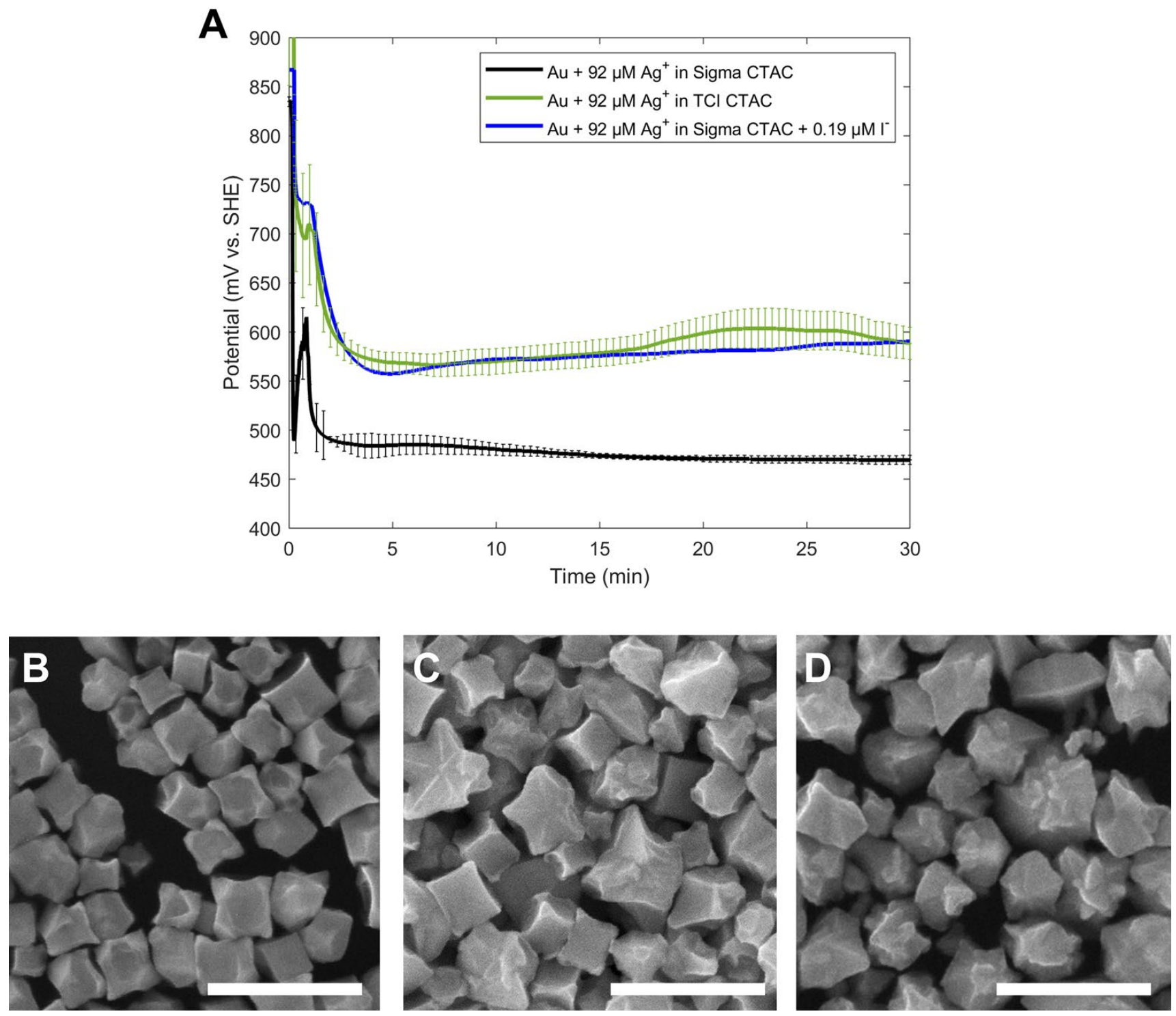

Figure S24. (A) OCP measurements taken with a DJ glass ORP sensor (Pt band WE vs. Ag/AgCl RE) of Au nanoparticle growth with 92 μM added Ag^{+}, in growth solutions made with as-received Sigma CTAC (black), as-received TCI CTAC (green), and as-received Sigma CTAC with 0.19 μM added iodide (blue). Error bars represent the standard deviation of three measurements. (B-D) SEM images of (Ag)Au nanoparticles synthesized with 92 μM Ag^{+} in as-received Sigma CTAC with (B) 0.15 μM, (C) 0.19 μM, and (D) 0.29 μM added iodide. Scale bars: 500 nm.

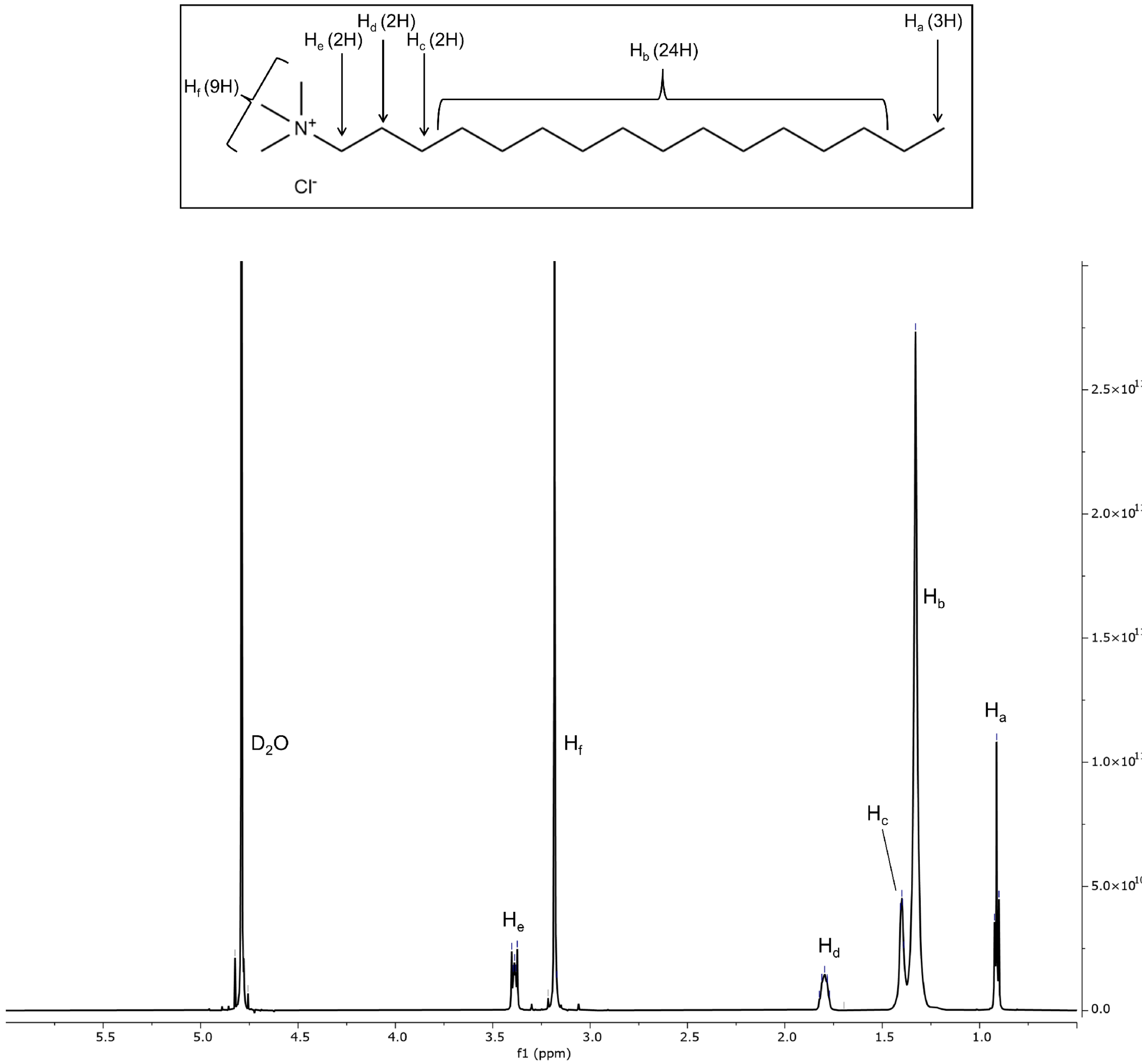

Figure S25. ^{1}H-NMR of as-received TCI CTAC in D_2O.

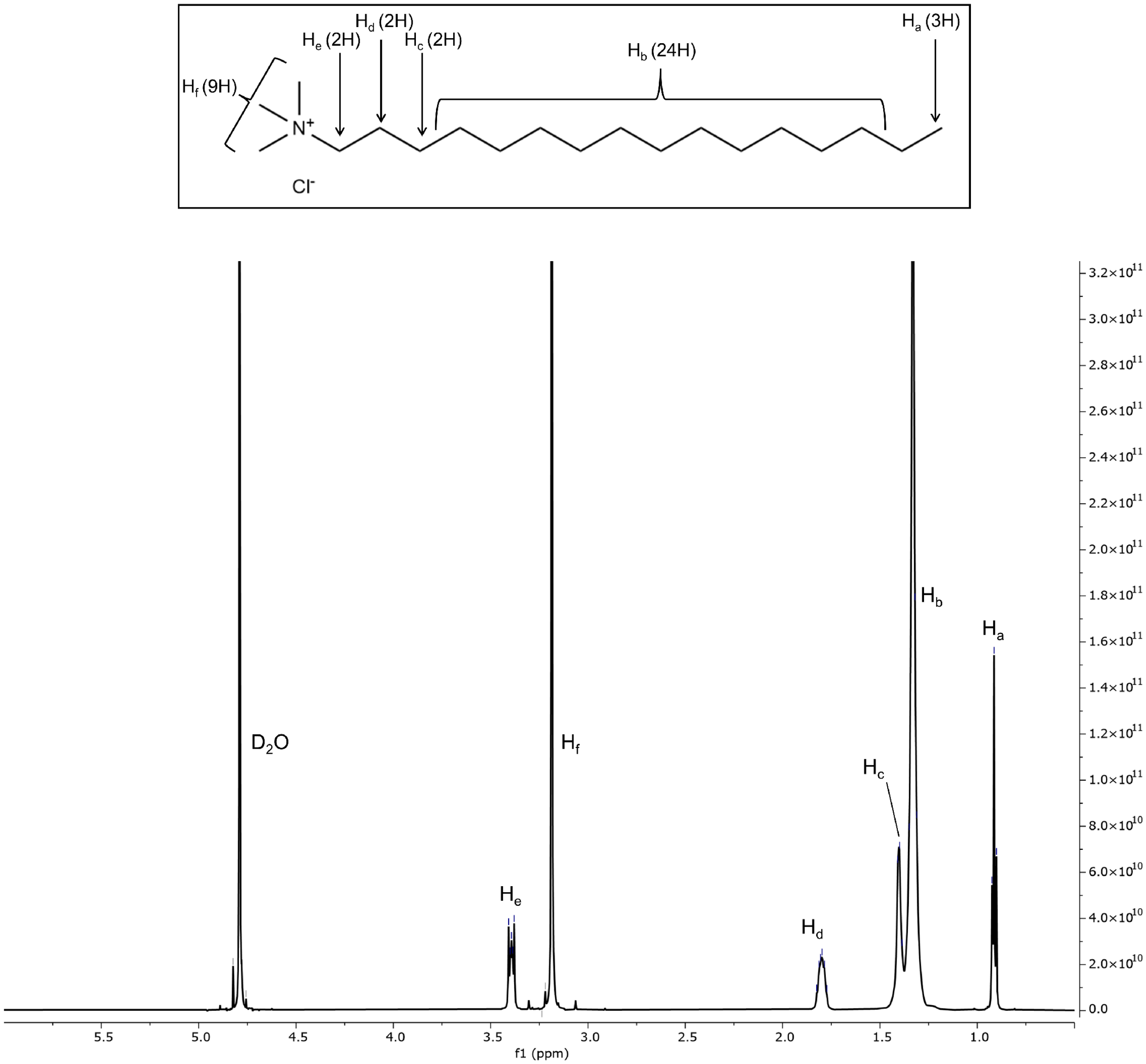

Figure S26. ^{1}H-NMR of recrystallized, oven-dried TCI CTAC in D_2O. The as-received (see Figure S25) and recrystallized TCI CTAB spectra show good agreement, with the absence of chemical shifts associated with recrystallization solvents: chloroform (~7.24 to 7.9 ppm in D_2O—poorly tabulated due to solubility issues), the acetyl signal from ethyl acetate (2.07 ppm in D_2O), the two sets of ethyl signals from ethyl acetate (1.24 ppm and 4.14 ppm in D_2O) or the methyl proton signal of acetone, which was used to wash CTAC solids during filtration (2.23 ppm in D_2O).[21]

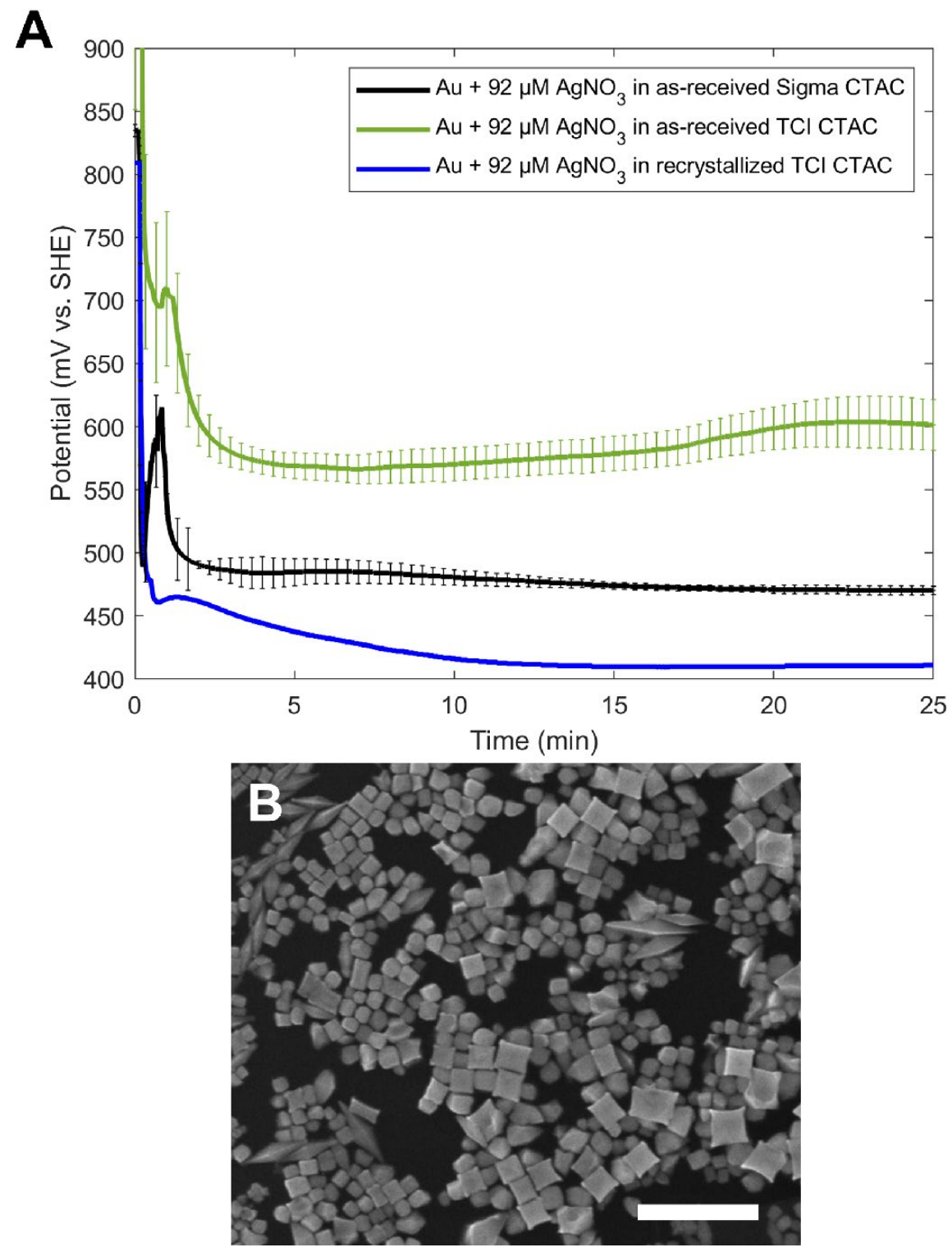

Figure S27. (A) OCP measurements taken with a DJ glass ORP sensor of (Ag)Au nanoparticle growth with 92 μM added Ag^+, in growth solutions made with as-received Sigma CTAC (black), as-received TCI CTAC (green), and recrystallized, oven-dried TCI CTAC (blue). Error bars represent the standard deviation of three measurements. (B) SEM image of (Ag)Au nanoparticles synthesized with 92 μM Ag^+ in a recrystallized, oven-dried TCI CTAC growth solution. The products are a mixture of {720} concave cubes and {310} truncated ditetragonal prisms and twinned bipyramidal particles, indicative of an unusually low Ag surface coverage for this input amount of Ag^+. Scale bar: 500 nm.

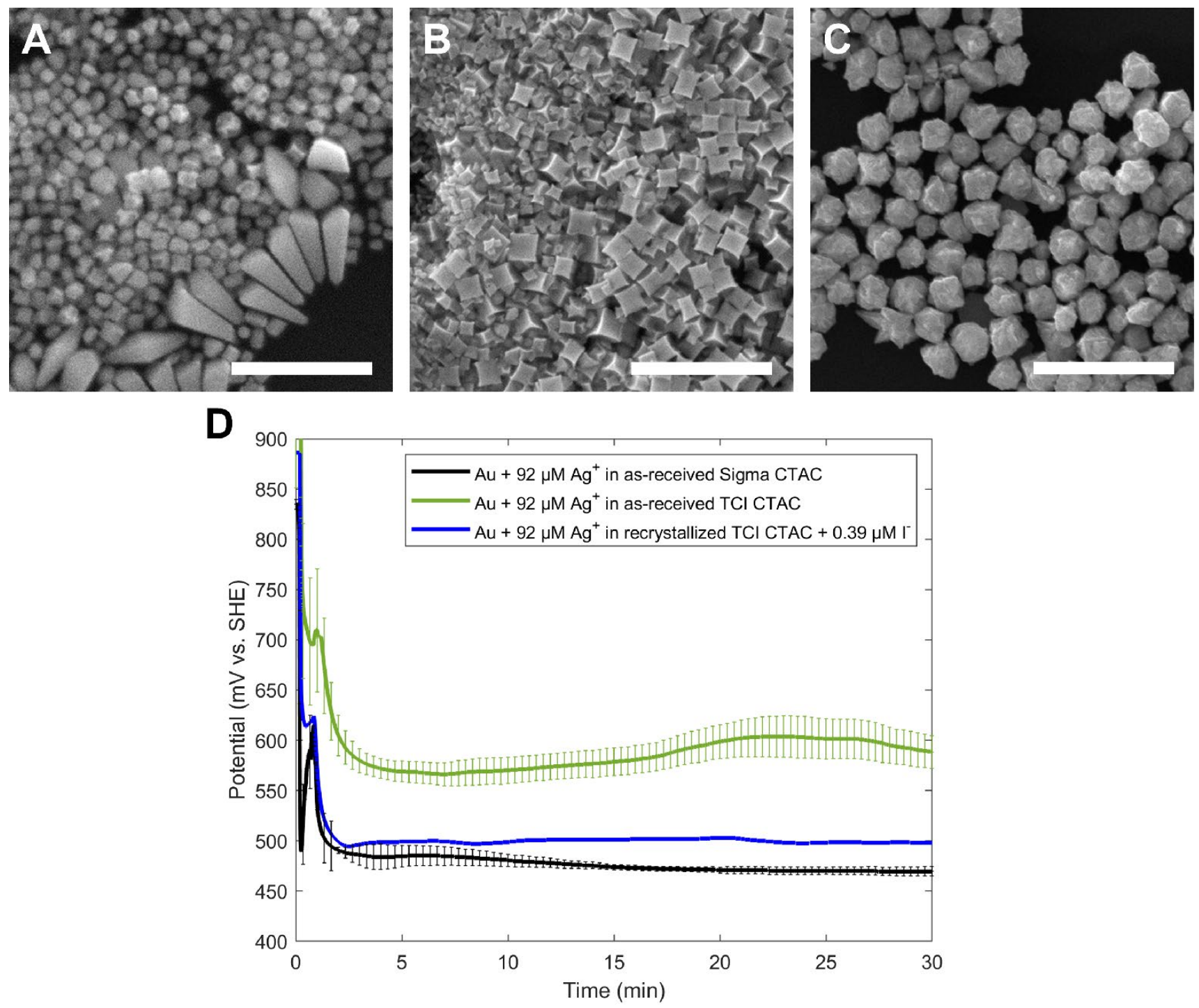

Figure S28. (A-C) SEM images of (Ag)Au nanoparticles grown in recrystallized, dried TCI CTAC with 92 μM Ag^+ and (A) 0.19 μM, (B) 0.39 μM, or (C) 0.49 μM of iodide added to the growth solution. (D) OCP measurements taken with a DJ glass ORP sensor of (Ag)Au nanoparticle growth with 92 μM added Ag^+, in growth solutions made with as-received Sigma CTAC (black), as-received TCI CTAC (green), and recrystallized, oven-dried TCI CTAC with 0.39 μM iodide added to the growth solution (blue). Error bars represent the standard deviation of three measurements. Scale bars: 500 nm.

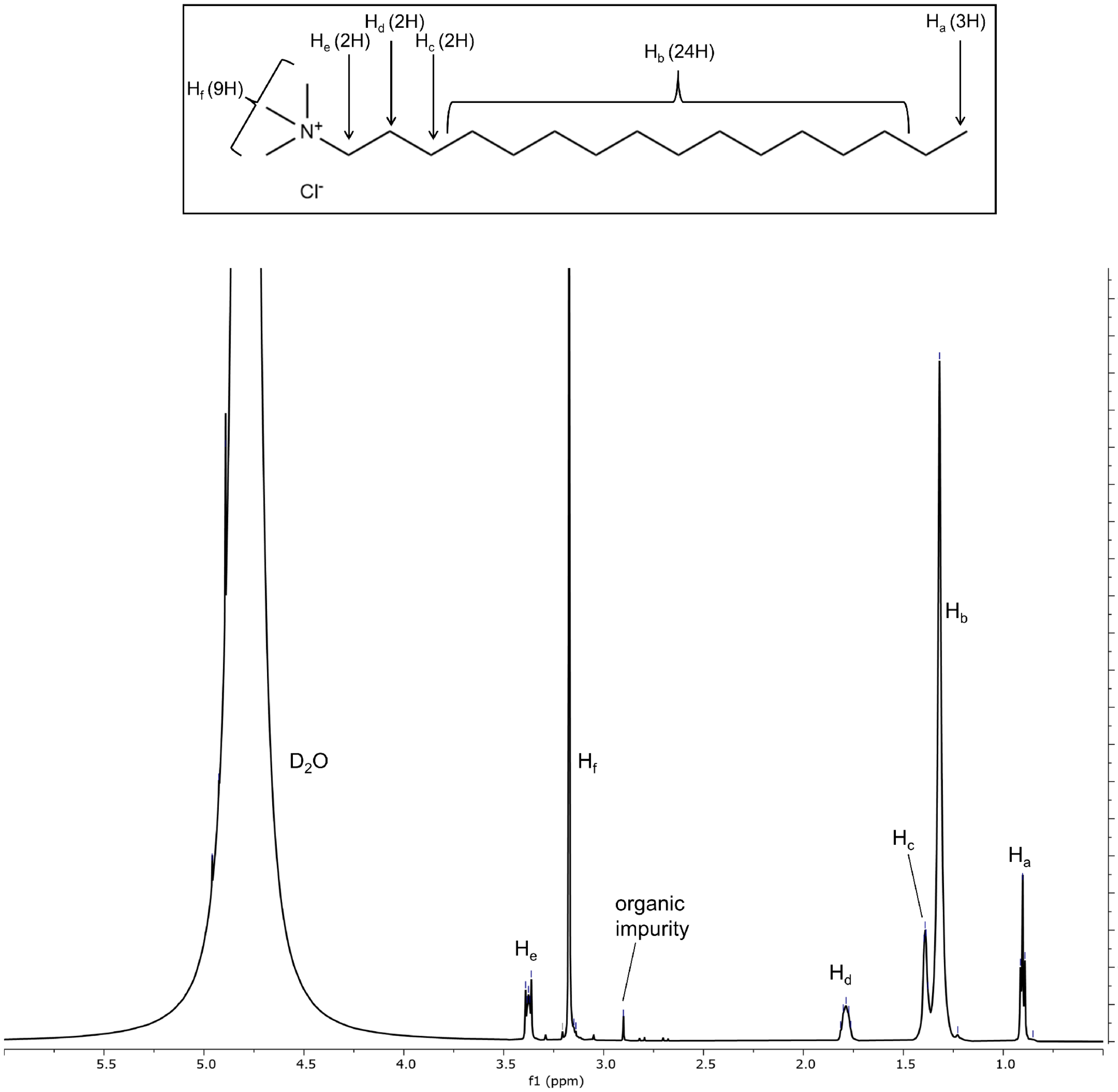

Figure S29. ^{1}H-NMR of as-received Sigma CTAC (25 wt. % aqueous solution) in 90% H_2O : 10% D_2O mixture. The labeled organic impurity peak at 2.90 ppm likely represents organic solvent contamination and could not be conclusively assigned. The following solvents, which either are used in industrial syntheses of CTAC and/or are known to have ~2.90 ppm singlet peaks in D_2O, were ruled out as the source of this impurity peak: acetone, methanol, ethanol, isopropanol, ethyl acetate, chloroform, 1,2-dichloroethane, dimethylacetamide, dimethylformamide, and pyrrolidone.[21]

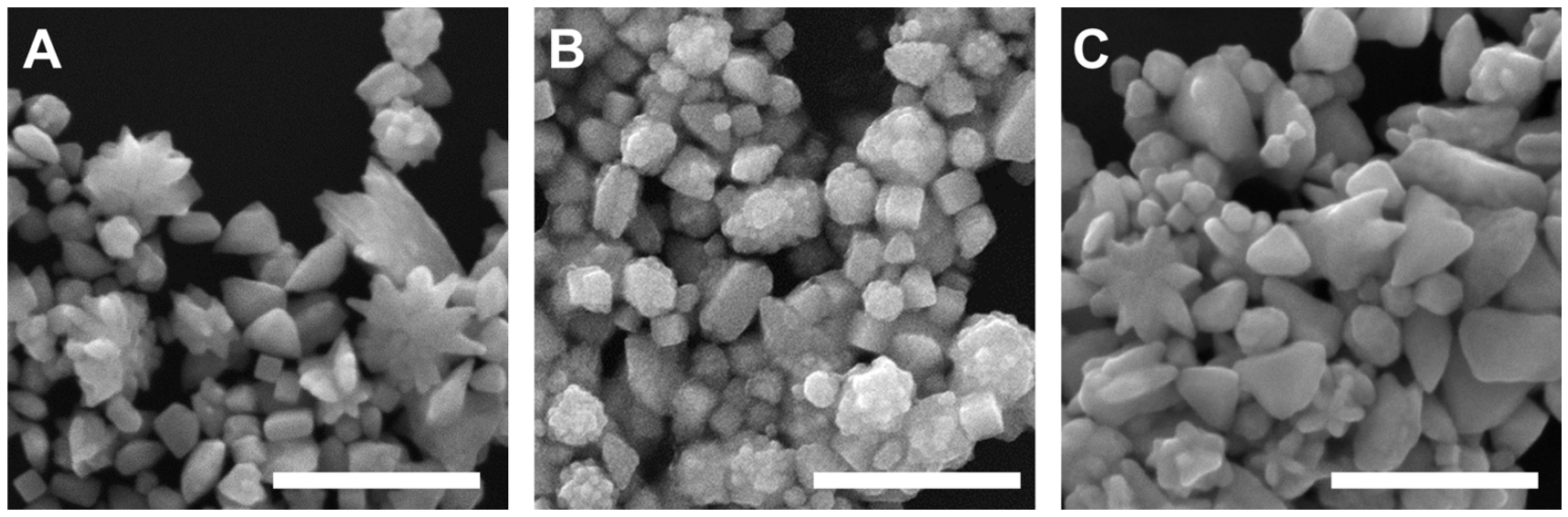

Figure S30. (A-C) Additional representative SEM images of Pd corrugated particles synthesized in as-received TCI CTAC (A) without OCP measurement and (B-C) with SJ epoxy ORP sensor measurement (Pt band WE vs. Ag/AgCl RE). Scale bars: 500 nm.

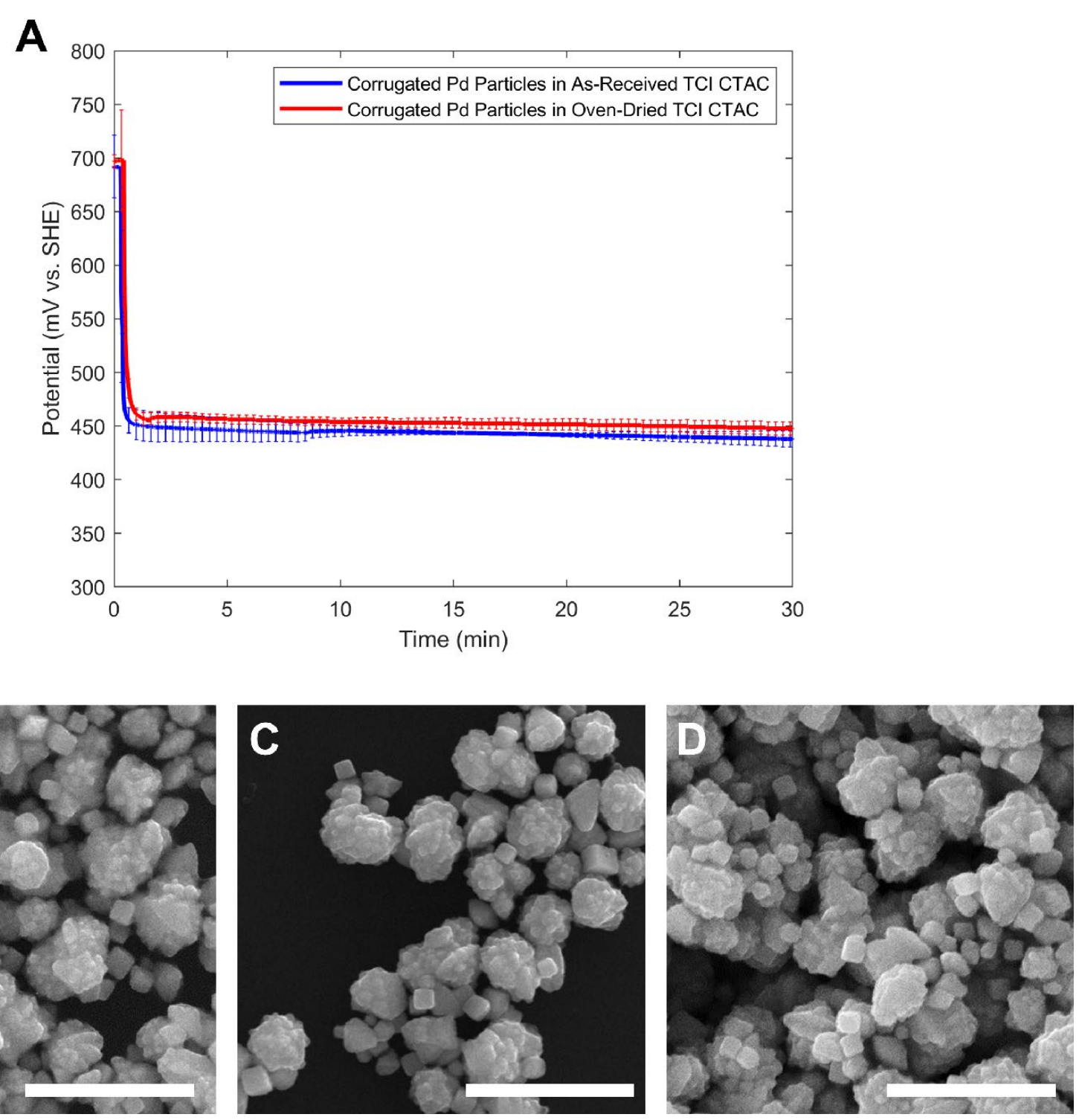

Figure S31. (A) SJ epoxy ORP measurements (Pt band WE vs. Ag/AgCl RE) of Pd corrugated particles in synthesized in as-received TCI CTAC (blue), and oven-dried TCI CTAC (red). Error bars represent the standard deviation of three measurements. (B-D) SEM images of Pd corrugated particles synthesized in oven-dried TCI CTAC. Scale bars: 500 nm.

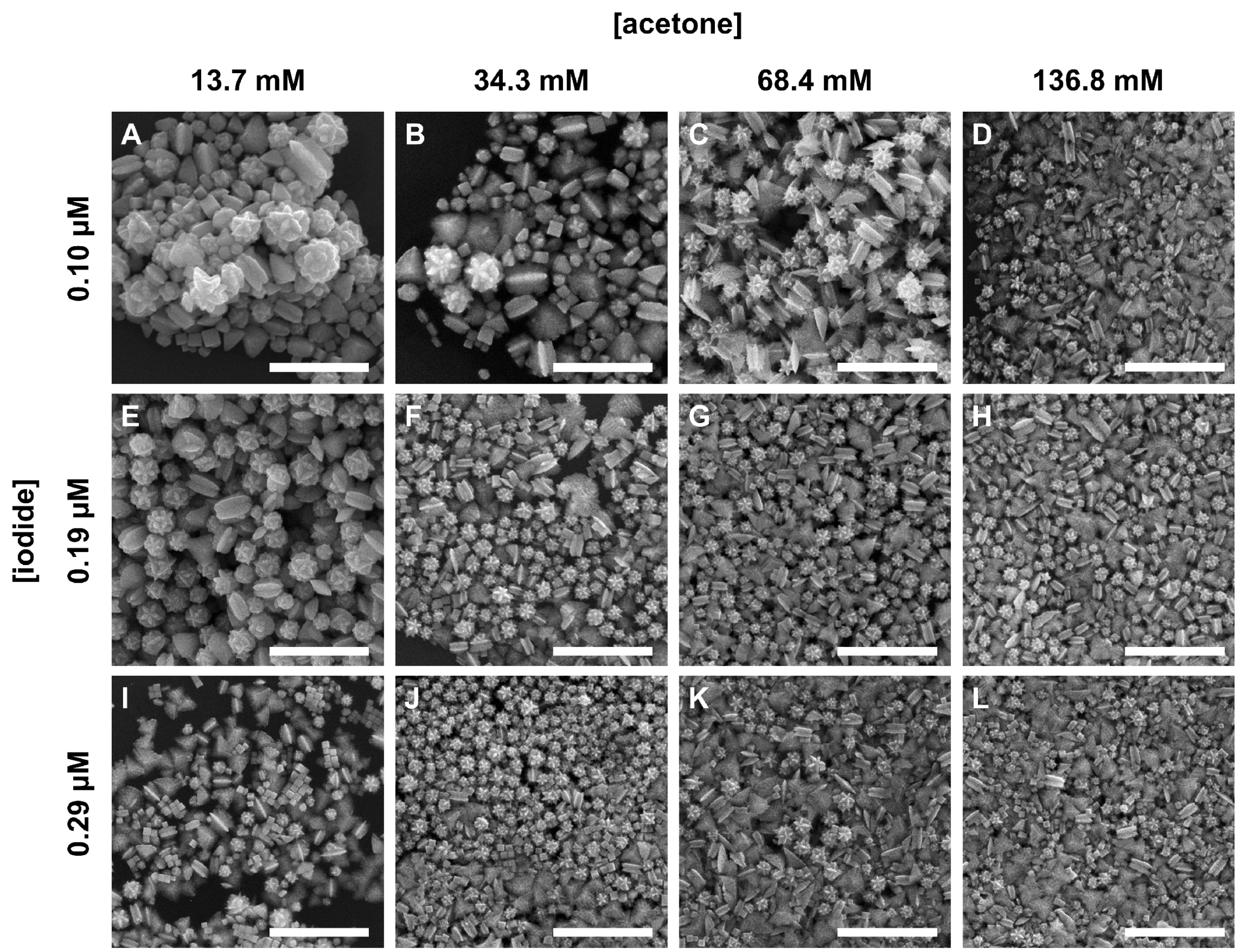

Figure S32. (A-L) SEM images of Pd corrugated particles synthesized in recrystallized, oven-dried TCI CTAC with a gradient of iodide and acetone additive concentrations. Volume of neat acetone in growth solution shown in parentheses. (A) 0.10 µM NaI and 13.7 mM acetone (10 µL), (B) 0.10 µM NaI and 34.3 mM acetone (25 µL), (C) 0.10 µM NaI and 68.4 mM acetone (50 µL), (D) 0.10 µM NaI and 136.8 mM acetone (100 µL), (E) 0.19 µM NaI and 13.7 mM acetone (10 µL), (F) 0.19 µM NaI and 34.3 mM acetone (25 µL), (G) 0.19 µM NaI and 68.4 mM acetone (50 µL), (H) 0.19 µM NaI and 136.8 mM acetone (100 µL) (I) 0.29 µM NaI and 13.7 mM acetone (10 µL), (J) 0.29 µM NaI and 34.3 mM acetone (25 µL), (K) 0.29 µM NaI and 68.4 mM acetone (50 µL), and (L) 0.29 µM NaI and 136.8 mM acetone (100 µL). As the concentration of either of both additives is increased, the corrugation of the particles increases and their size decreases. The particles in image (C) represent the optimal size and morphology, comparable to results in as-received Sigma CTAC. Note that all samples were synthesized using the same batch of Pd seeds. Scale bars: 1 µm.

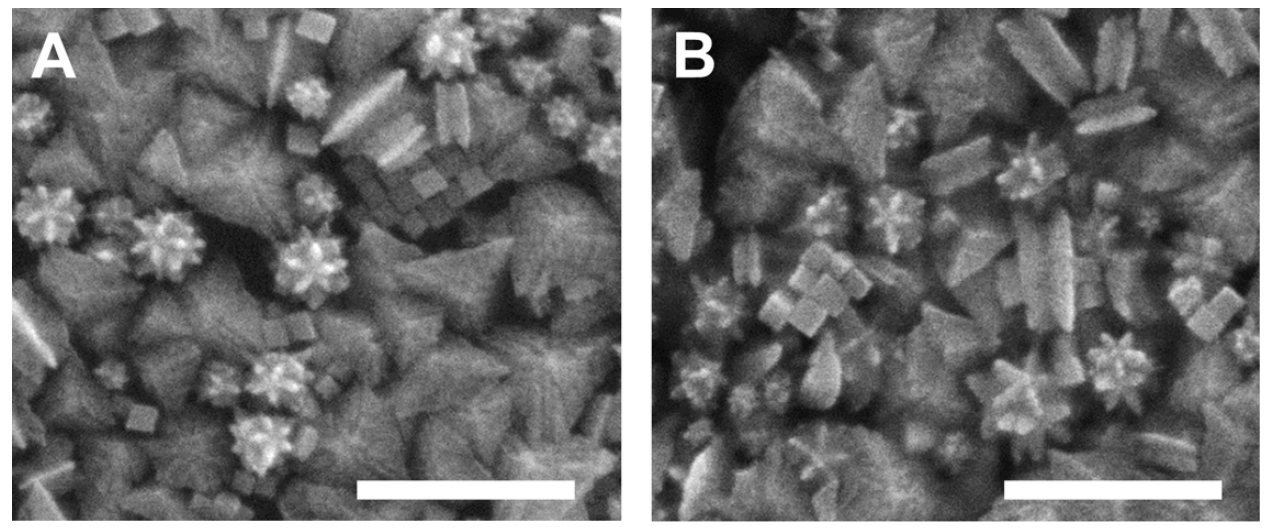

Figure S33. (A-B) Additional representative SEM images of Pd corrugated particles synthesized in recrystallized, oven dried TCI CTAC with 0.10 μM NaI and 50 μL neat acetone (equal to 68.4 mM acetone in the growth solution). Scale bars: 500 nm.

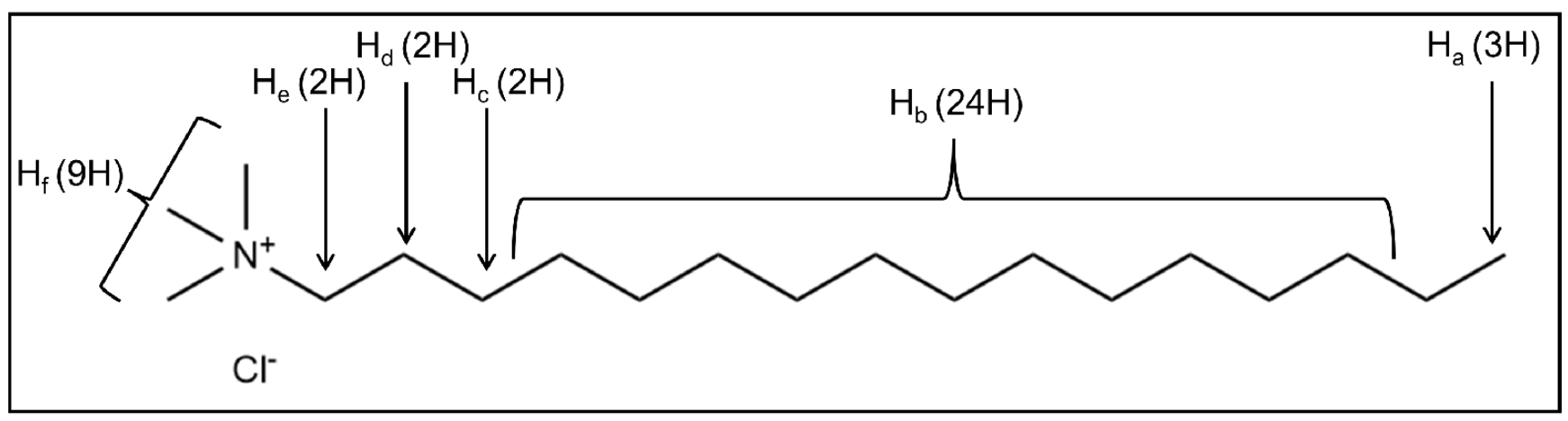

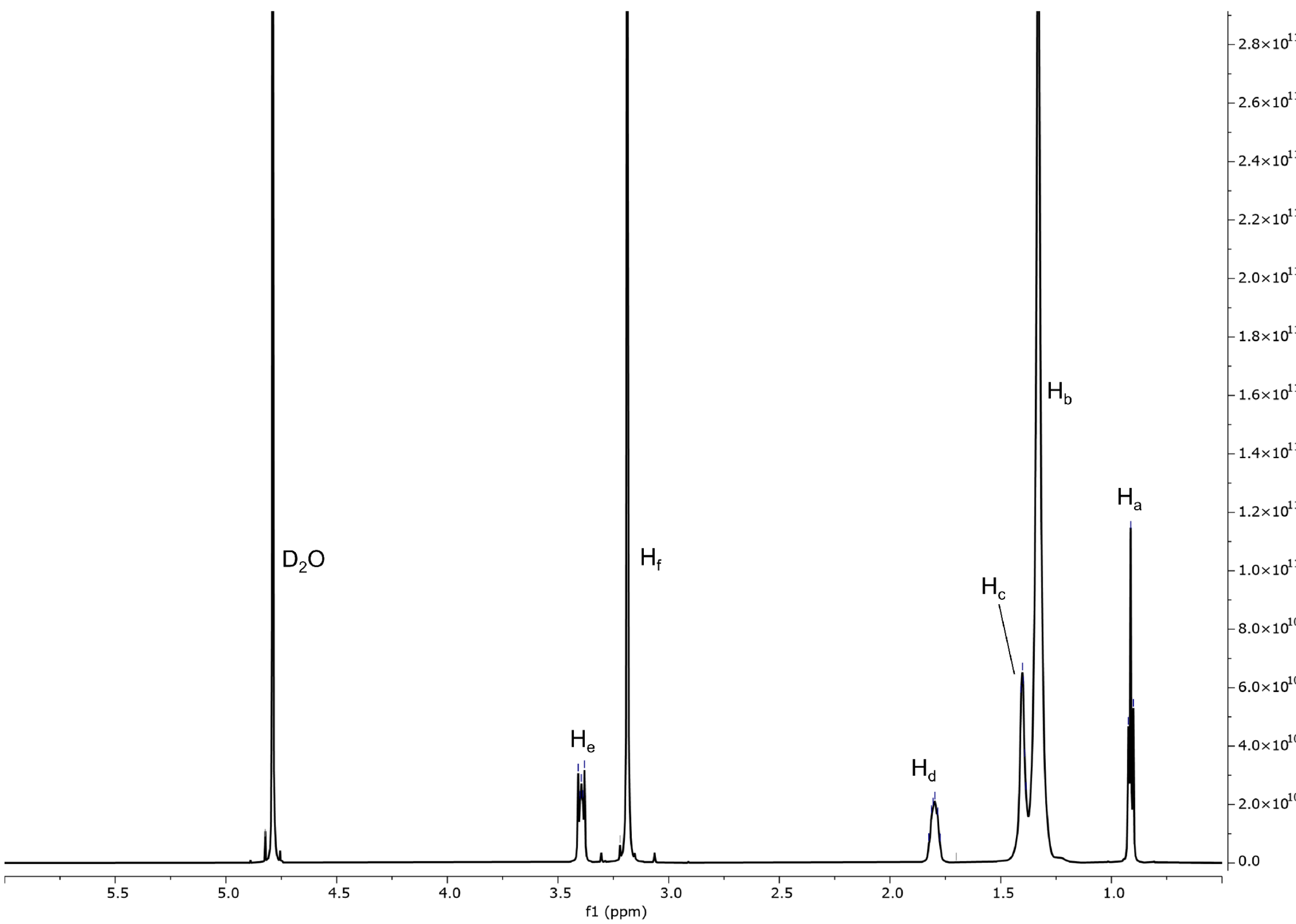

Figure S34. ^{1}H-NMR of lyophilized Sigma CTAC in D_2O. The 2.90 ppm impurity peak seen in as-received Sigma CTAC is absent.

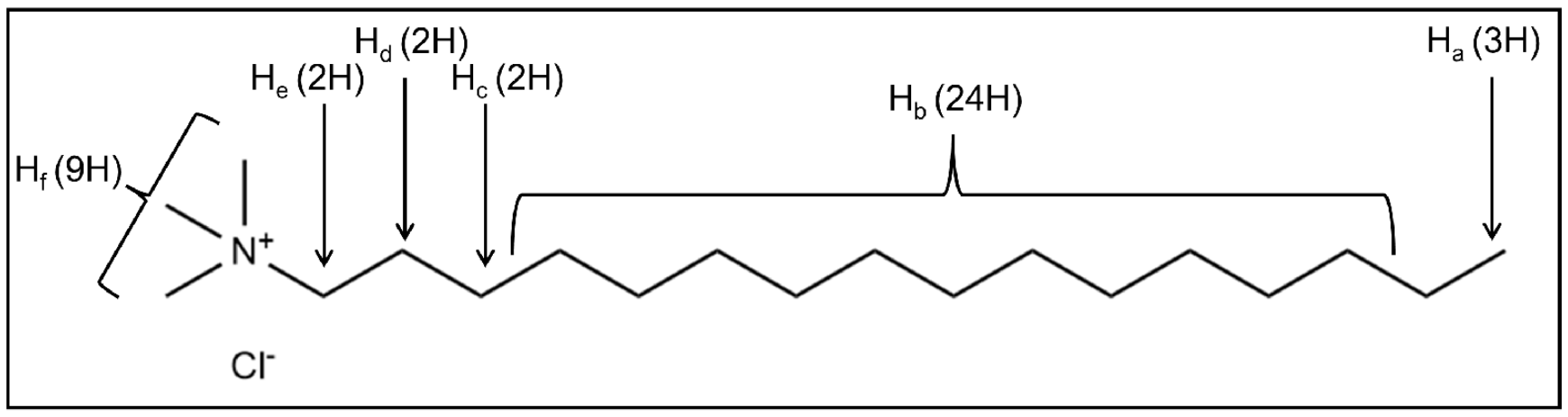

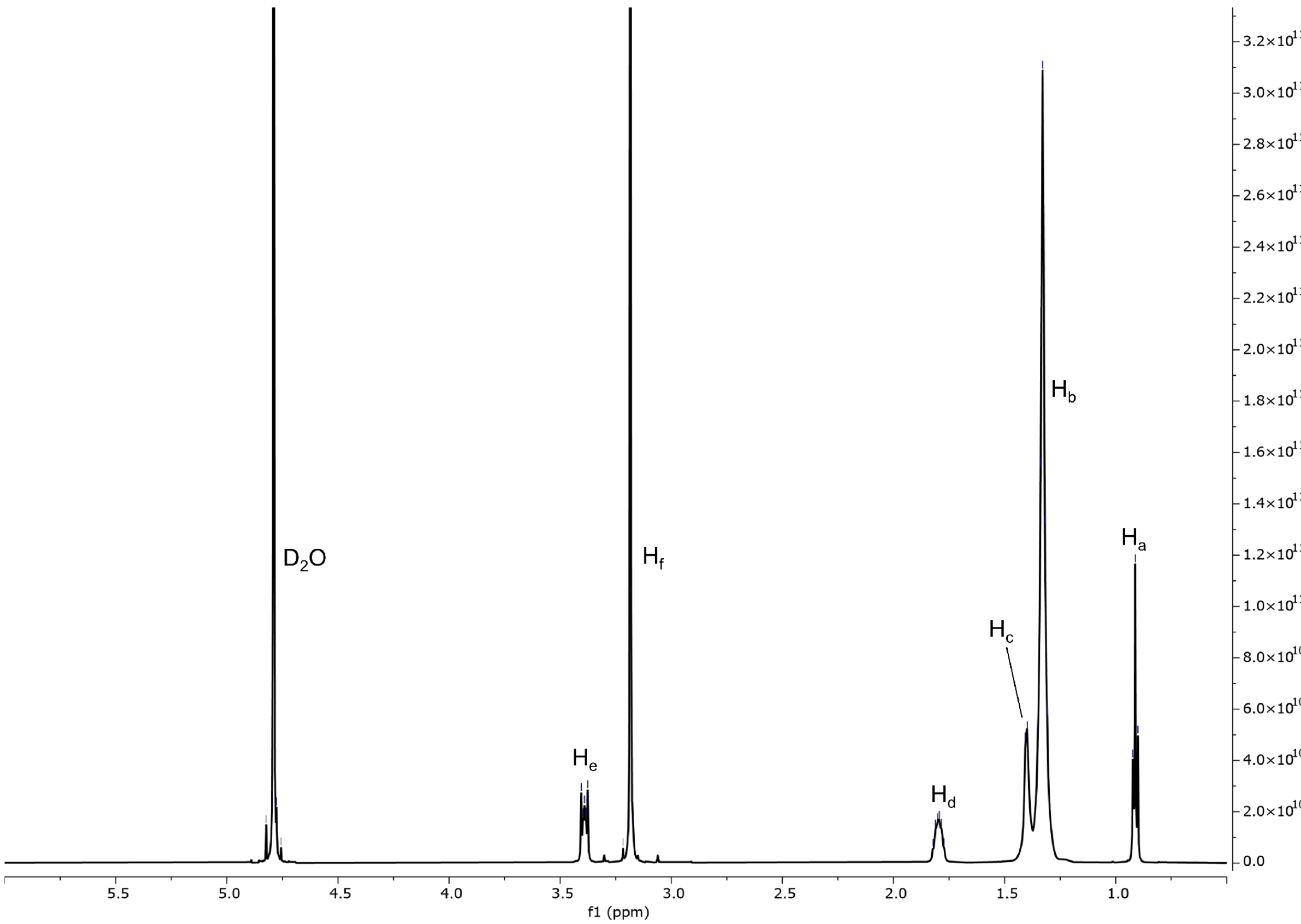

Figure S35. ^{1}H-NMR of lyophilized, recrystallized, oven-dried Sigma CTAC in D_2O. The 2.90 ppm impurity peak seen in as-received Sigma CTAC is absent, as are impurity peaks associated with recrystallization solvents.

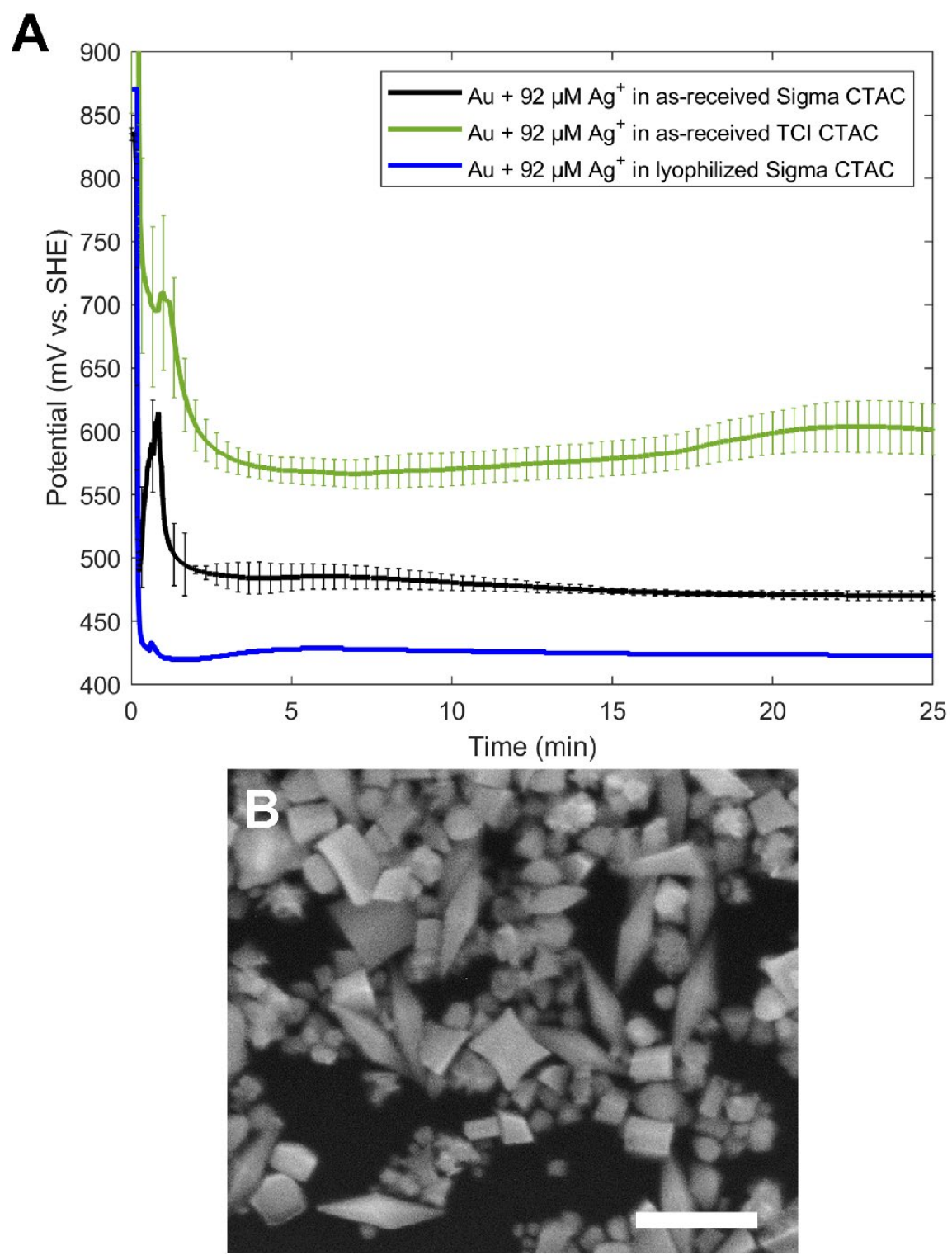

Figure S36. (A) DJ glass ORP measurements (Pt band WE vs. Ag/AgCl RE) of (Ag)Au nanoparticle growth with 92 μM added Ag^+, in growth solutions made with as-received Sigma CTAC (black), as-received TCI CTAC (green), and lyophilized Sigma CTAC (blue). Error bars represent the standard deviation of three measurements. (B) SEM image of (Ag)Au nanoparticles synthesized with 92 μM Ag^+ in a lyophilized Sigma CTAC growth solution. The products are a mixture of {720} concave cubes and {310} truncated ditetragonal prisms and twinned particles, indicative of an unusually low Ag surface coverage for this input concentration of Ag^+. Scale bar: 500 nm.

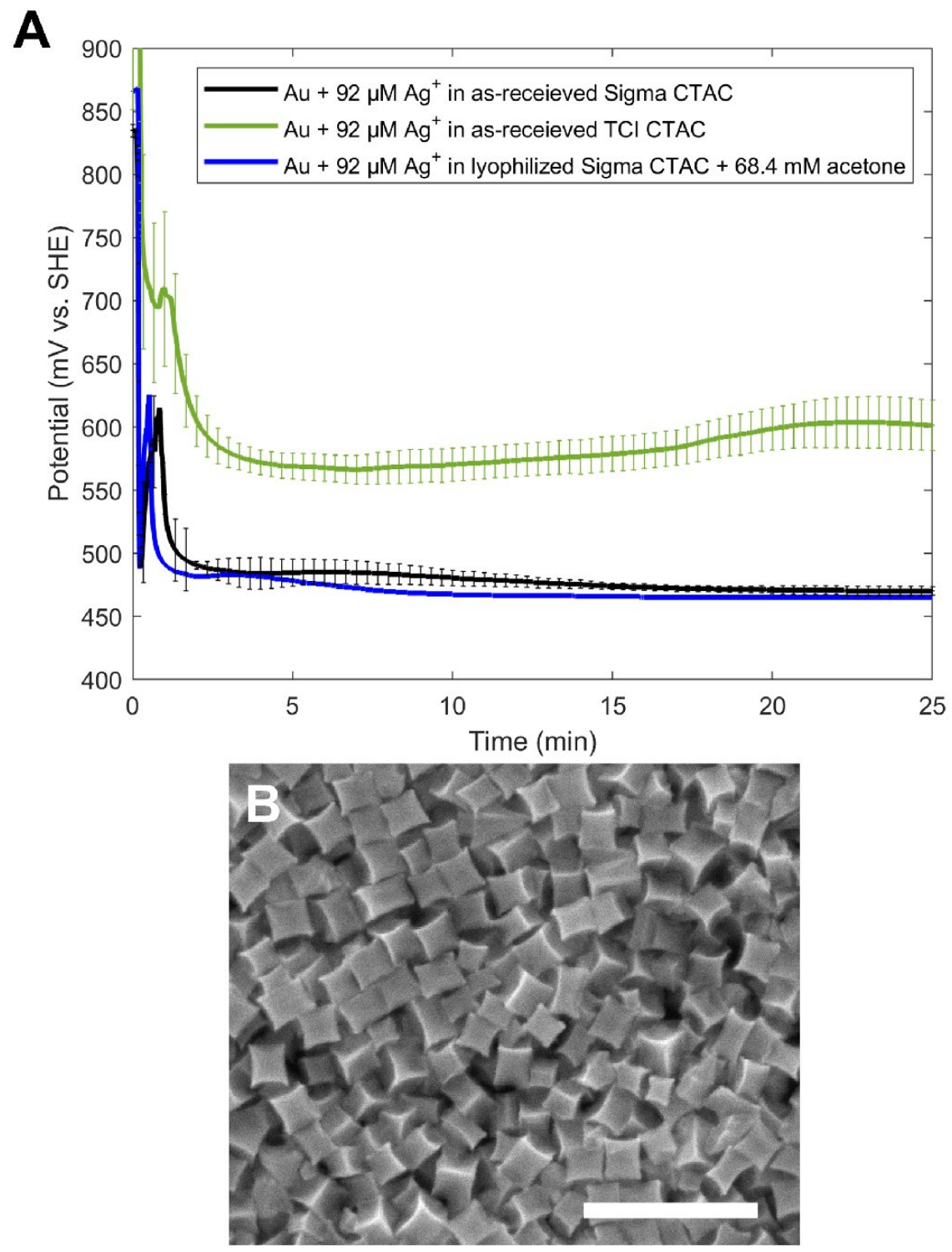

Figure S37. (A) DJ glass ORP measurements (Pt band WE vs. Ag/AgCl RE) of (Ag)Au nanoparticle growth with 92 µM added Ag^{+}, in growth solutions made with as-received Sigma CTAC (black), as-received TCI CTAC (green), and lyophilized Sigma CTAC with 50 µL of neat acetone (68.4 mM in the growth solution, blue). Error bars represent the standard deviation of three measurements. (B) SEM image of (Ag)Au concave cubic nanoparticles synthesized with 92 µM Ag^{+} in a lyophilized Sigma CTAC growth solution with 50 µL of neat acetone (68.4 mM). Scale bar: 500 nm.

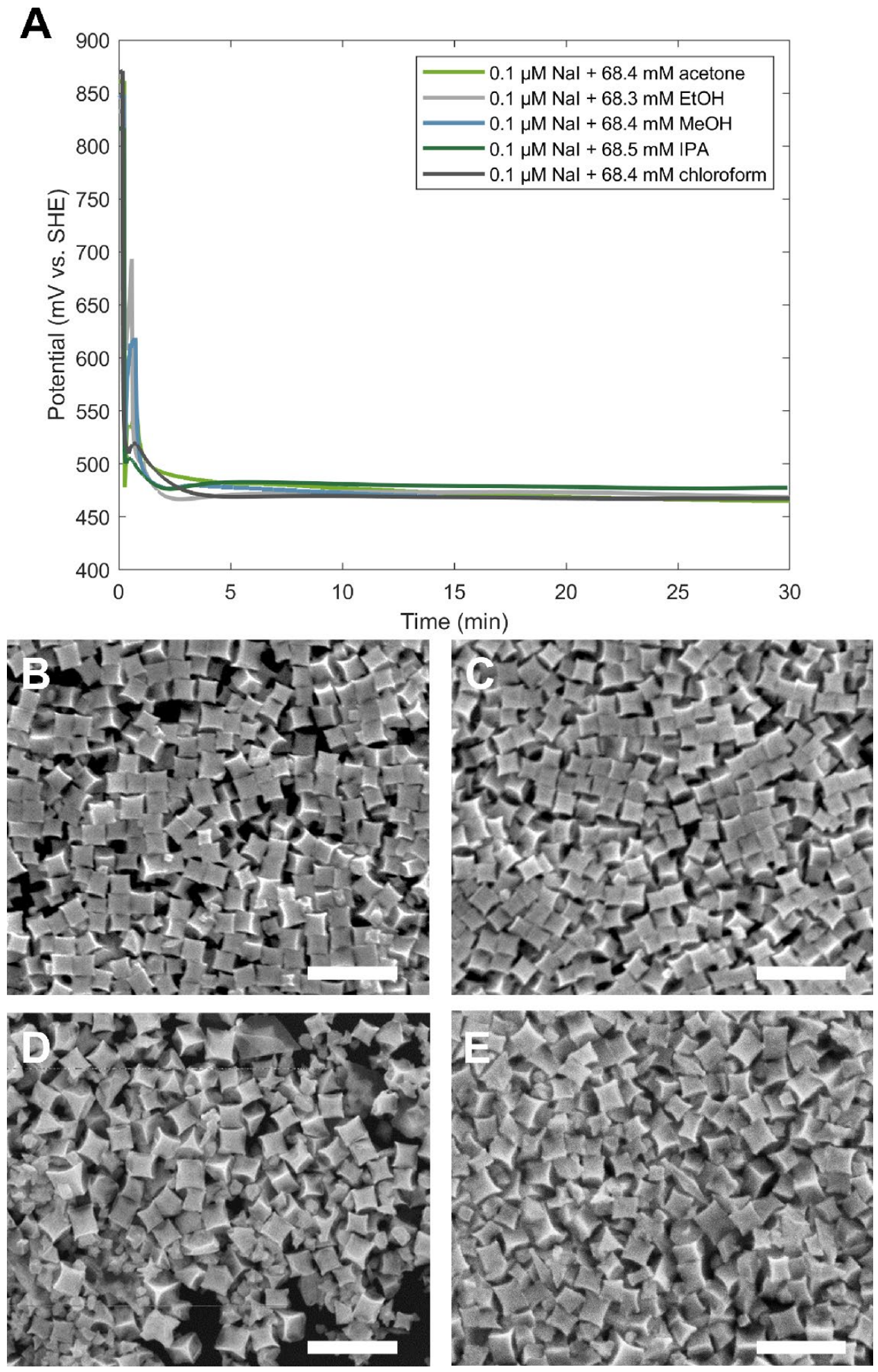

Figure S38. (A) DJ glass ORP measurements (Pt band WE vs. Ag/AgCl RE) of Au nanoparticle growth with 92 µM added Ag^+, in growth solutions made with recrystallized, oven-dried TCI CTAC, 0.10 µM added iodide, and ~68.4 mM concentrations of various organic solvent additives in the growth solution. (B-E) SEM images of (Ag)Au concave cubic nanoparticles synthesized with 92 µM Ag^+ in growth solutions made with recrystallized, oven-dried TCI CTAC, 0.10 µM added iodide, and (B) 68.3 mM ethanol (EtOH), (C) 68.4 mM methanol (MeOH), (D) 68.5 mM isopropanol (IPA), or (E) 68.4 mM chloroform. Scale bars: 500 nm.

References

(1) Halford, G. C.; McDarby, S. P.; Hertle, S.; Kiely, A. F.; Luu, J. T.; Wang, C. J.; Personick, M. L. Troubleshooting the Influence of Trace Chemical Impurities on Nanoparticle Growth Kinetics *via* Electrochemical Measurements. *Nanoscale* **2024**, *16*, 11038–11051. https://doi.org/10.1039/D4NR00070F.

(2) Zana, R.; Benrraou, M.; Rueff, R. Alkanediyl-α,ω-Bis(Dimethylalkylammonium Bromide) Surfactants. 1. Effect of the Spacer Chain Length on the Critical Micelle Concentration and Micelle Ionization Degree. *Langmuir* **1991**, *7*, 1072–1075. https://doi.org/10.1021/la00054a008.

(3) K. M. Koczkur and S. E. Skrabalak, personal communication.

(4) King, M. E.; Personick, M. L. Defects by Design: Synthesis of Palladium Nanoparticles with Extended Twin Defects and Corrugated Surfaces. *Nanoscale* **2017**, *9*, 17914–17921. https://doi.org/10.1039/C7NR06969C.

(5) Reddy Satyavolu, N. S.; Peinetti, A. S.; Wang, Y.; Ali, A. S.; Lin, J. W.; Lu, Y. Silver-Assisted Synthesis of High-Indexed Palladium Tetrahexahedral Nanoparticles and Their Morphological Variants. *Chem. Mater.* **2019**, *31*, 2923–2929. https://doi.org/10.1021/acs.chemmater.9b00275.

(6) Zhang, G.; Feng, J.; Wang, S.; Xu, B.; Ding, W.; Wang, W.; Fu, Z. Stable Pd@Cu Core–Shell Nanocubes with Finely Tuned Sizes for the Reduction of Nitroaromatics. *ACS Appl. Nano Mater.* **2019**, *2*, 4584–4593. https://doi.org/10.1021/acsanm.9b00946.

(7) Zhang, J.; Langille, M. R.; Personick, M. L.; Zhang, K.; Li, S.; Mirkin, C. A. Concave Cubic Gold Nanocrystals with High-Index Facets. *J. Am. Chem. Soc.* **2010**, *132*, 14012–14014. https://doi.org/10.1021/ja106394k.

(8) Halford, G. C.; Sublett, A. M.; Personick, M. L. Real-Time Electroanalytical Measurements of Dynamic Reaction Chemistry Provide Generalizable Mechanistic Considerations for Metal Nanoparticle Synthesis. *J. Am. Chem. Soc.* **2026**, *148*, 7280–7299. https://doi.org/10.1021/jacs.5c19321.

(9) Personick, M. L.; Langille, M. R.; Zhang, J.; Mirkin, C. A. Shape Control of Gold Nanoparticles by Silver Underpotential Deposition. *Nano Lett.* **2011**, *11*, 3394–3398. https://doi.org/10.1021/nl201796s.

(10) Thoka, S.; Madasu, M.; Hsia, C.; Liu, S.; Huang, M. H. Aqueous-Phase Synthesis of Size-Tunable Copper Nanocubes for Efficient Aryl Alkyne Hydroboration. *Chem. Asian J.* **2017**, *12*, 2318–2322. https://doi.org/10.1002/asia.201700856.

(11) Roger, I.; Symes, M. D. Silver Leakage from Ag/AgCl Reference Electrodes as a Potential Cause of Interference in the Electrocatalytic Hydrogen Evolution Reaction. *ACS Appl. Mater. Interfaces* **2017**, *9* (1), 472–478. https://doi.org/10.1021/acsami.6b13438.

(12) Bard, A. J.; Parsons, R.; Jordan, J. Copper, Silver, and Gold. In *Standard Potentials in Aqueous Solution*; Routledge, 1985. https://doi.org/10.1201/9780203738764.

(13) Bard, A. J.; Faulkner, L. R.; White, H. S. *Electrochemical Methods: Fundamentals and Applications*, Third edition.; Wiley: Hoboken, NJ, 2022.

(14) O'Mullane, A. P.; Bhargava, S. K. Reduction of Au^{3+} Ions by Activated Surface Atoms of Platinum. *Electrochem. Commun.* **2011**, *13*, 852–855. https://doi.org/10.1016/j.elecom.2011.05.020.

(15) Ambrozik, S.; Mitchell, C.; Dimitrov, N. The Spontaneous Deposition of Au on Pt (111) and Polycrystalline Pt. *J. Electrochem. Soc.* **2016**, *163*, D3001–D3007. https://doi.org/10.1149/2.0011612jes.

(16) Chrzanowski, W.; Wieckowski, A. Ultrathin Films of Ruthenium on Low Index Platinum Single Crystal Surfaces: An Electrochemical Study. *Langmuir* **1997**, *13*, 5974–5978. https://doi.org/10.1021/la970193c.

(17) Chrzanowski, W.; Kim, H.; Wieckowski, A. Enhancement in Methanol Oxidation by Spontaneously Deposited Ruthenium on Low-Index Platinum Electrodes. *Catal. Lett.* **1998**, *50*, 69–75. https://doi.org/10.1023/A:1019042329841.

(18) Vigier, F.; Gloaguen, F.; Léger, J.-M.; Lamy, C. Electrochemical and Spontaneous Deposition of Ruthenium at Platinum Electrodes for Methanol Oxidation: An Electrochemical Quartz Crystal Microbalance Study. *Electrochim. Acta* **2001**, *46*, 4331–4337. https://doi.org/10.1016/S0013-4686(01)00680-6.

(19) Crown, A.; Johnston, C.; Wieckowski, A. Growth of Ruthenium Islands on Pt(*hkl*) Electrodes Obtained via Repetitive Spontaneous Deposition. *Surf. Sci.* **2002**, *506*, L268–L274. https://doi.org/10.1016/S0039-6028(02)01317-1.

(20) Strbac, S.; Behm, R. J.; Crown, A.; Wieckowski, A. In Situ STM Imaging of Spontaneously Deposited Ruthenium on Au(111). *Surf. Sci.* **2002**, *517*, 207–218. https://doi.org/10.1016/S0039-6028(02)02062-9.

(21) Fulmer, G. R.; Miller, A. J. M.; Sherden, N. H.; Gottlieb, H. E.; Nudelman, A.; Stoltz, B. M.; Bercaw, J. E.; Goldberg, K. I. NMR Chemical Shifts of Trace Impurities: Common Laboratory Solvents, Organics, and Gases in Deuterated Solvents Relevant to the Organometallic Chemist. *Organometallics* **2010**, *29*, 2176–2179. https://doi.org/10.1021/om100106e.
